# Socioeconomic Status, Alcohol Use, and Mortality: Results From a Prospective Cohort Study

**DOI:** 10.1111/acer.70402

**Published:** 2026-09-05

**Authors:** Ida Butovetsky, Sophie Baumann, Hans J. Grabe, Carsten Oliver Schmidt, Astrid Petersmann, Matthias Nauck, Lars Schwettmann

**Affiliations:** ^1^ Division of Health Economics, Department of Health Services Research, School VI ‐ School of Medicine and Health Sciences Carl von Ossietzky Universität Oldenburg Oldenburg Germany; ^2^ Department of Methods in Community Medicine, Institute for Community Medicine University Medicine Greifswald Greifswald Germany; ^3^ Department of Psychiatry and Psychotherapy University Medicine Greifswald Greifswald Germany; ^4^ Institute for Community Medicine, Section Study of Health in Pomerania—Clinical‐Epidemiological Research University Medicine Greifswald Greifswald Germany; ^5^ Institute for Clinical Chemistry and Laboratory Medicine University Medicine Oldenburg Oldenburg Germany; ^6^ Institute for Clinical Chemistry and Laboratory Medicine University Medicine Greifswald Greifswald Germany; ^7^ DZHK (German Centre for Cardiovascular Research), Partner Site Greifswald University Medicine Greifswald Greifswald Germany

**Keywords:** alcohol drinking, mortality, socioeconomic status, survival analysis

## Abstract

**Background:**

Both all‐cause and alcohol mortality follow a social gradient. Studies have provided evidence of interaction between socioeconomic status (SES) and alcohol use in predicting alcohol‐attributable mortality. It is unclear whether this holds true for all‐cause mortality. This study examines interaction effects and investigates the role of biomarkers in all‐cause mortality.

**Methods:**

We included data from 4307 participants of the baseline survey (1997–2001) of SHIP (Study of Health in Pomerania)‐START and a mortality follow‐up from baseline until January 2023. All‐cause mortality was the outcome; exposure variables were income or education as SES proxies, alcohol use, and biomarkers related to chronic alcohol consumption. Confounders were age, sex, marital status, smoking, and Body Mass Index (BMI). We used Cox proportional hazards regression (CPHR) in main analyses and CPHR and Aalen additive hazards regression (AAHR) in interaction analyses.

**Results:**

Our final samples (either with income or education) consisted of 3771/3939 individuals of whom 981 (26.0%) and 1047 (28.6%) died during the follow‐up period. Adjusting for confounders, individuals with low income (HR = 1.31, CI = 1.11, 1.56; compared to high income) and both current abstainers that formerly consumed alcohol (HR = 1.56, CI = 1.27, 1.92) and individuals with high alcohol intake (HR = 1.31, CI = 1.03, 1.66; compared to individuals with low alcohol intake) showed increased mortality. Biomarkers were positively associated with mortality. There was evidence of statistical interaction effects, for example, increased risks in low‐income current abstainers as well as individuals with moderate or high alcohol use.

**Conclusions:**

We found a social gradient in all‐cause mortality that was attenuated, but not fully explained, by risk factors. There was some evidence of statistical interaction effects between alcohol use and SES, but small subgroup counts limit the certainty of related conclusions.

## Graphical abstract

This study examines associations of income and education, self‐reported alcohol use and alcohol biomarkers with all‐cause mortality using data from the Study of Health in Pomerania (SHIP). We observed a social gradient that was attenuated, but not fully explained by alcohol use, biomarkers, and further risk factors. There was some evidence of statistical interaction effects of alcohol use by income or education, but a comparatively small sample size limits the certainty of related conclusions.

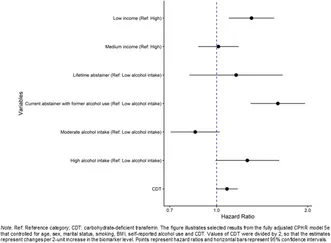

## 1 Introduction

According to the WHO's most recent global status report, alcohol accounted for 2.6 million deaths (i.e., 4.7% of all deaths) in 2019 (World Health Organization 2024). Furthermore, both all‐cause and alcohol‐attributable mortality seem to follow a social gradient, that is, the lower the socioeconomic status (SES), the higher the respective risk (Marmot 2005; Probst et al. 2021). Socioeconomic inequalities in health outcomes might be explained by *differential exposure to* or *differential effect of* risk factors in different socioeconomic groups (Diderichsen et al. 2019).

Differential exposure to alcohol consumption has widely been studied as an explanation for social gradients in mortality. Several studies investigated what proportion of social disparities in all‐cause and alcohol‐attributable mortality can be attributed to differences in alcohol use (Boyd et al. 2023; Peña, Mäkelä, Laatikainen, et al. 2021; Probst et al. 2020; Puka et al. 2022; Zhu et al. 2024). Estimates ranged from −6% to 17% in eight studies identified by a systematic review (Probst et al. 2020) and from −69.3% to 16% in studies published since then (Boyd et al. 2023; Peña, Mäkelä, Laatikainen, et al. 2021; Puka et al. 2022; Zhu et al. 2024). Accounting for heavy episodic drinking (HED) instead of alcohol quantity measures, estimates reach 27% at most, that is, 27% of educational differences in all‐cause mortality can be explained by HED (Probst et al. 2020; Sydén and Landberg 2017). Thus, social gradients in all‐cause and alcohol‐attributable mortality are, if at all, attenuated but not fully explained by differential alcohol exposure.

In contrast, a differential effect would mean that individuals with low SES suffer more severe consequences from the same amount of alcohol consumption. This might be measured as a statistical interaction of SES and alcohol use and has been studied less extensively. Some works, however, show statistical interaction effects in alcohol‐attributable hospitalization and mortality (Christensen et al. 2017; Peña, Mäkelä, Laatikainen, et al. 2021; Probst et al. 2025; Smith et al. 2024) as well as in all‐cause mortality (Puka et al. 2022; Zhu et al. 2026). However, Puka et al. (2022) showed a statistical interaction effect of low education and high alcohol use on all‐cause mortality only for women, while Zhu et al. (2026) detected a three‐way interaction effect of low education, drinking above 60 g per day and the time period after 2010 only for men.

As many studies use self‐reported measures of alcohol use, *measurement error* has been discussed as another explanation (Boyd et al. 2022) since these measures come with risks of bias, for example, under‐reporting due to social desirability (Davis et al. 2010) or recall bias (Cherpitel et al. 2018). There is some evidence that measurement error differs between socioeconomic groups. However, after correcting for self‐report bias, higher rates of hazardous drinking were found in individuals with high education, that is, the positive association between education and hazardous drinking even increased (Devaux and Sassi 2016), which contrasts with the idea of differential exposure.

Nevertheless, to account for differential measurement error in self‐reported alcohol use, Peña, Mäkelä, Härkänen, et al. (2021) investigated the association between alcohol use and alcohol‐attributable mortality while adjusting for biomarkers related to chronic alcohol consumption, that is, gamma‐glutamyltransferase (GGT), alanine transaminase (ALT), aspartate transaminase (AST) and carbohydrate‐deficient transferrin (CDT). Using these biomarkers beyond self‐reported alcohol use improved the predictive ability of their models but only slightly attenuated the social gradient in alcohol‐attributable mortality. This means that social inequalities in alcohol‐attributable mortality could neither be explained by differences in self‐reported alcohol use nor by differences in objective alcohol biomarkers. At the same time, the paper by Peña, Mäkelä, Härkänen, et al. (2021) remains the only study to test for the explanatory value of alcohol biomarkers beyond self‐reported alcohol use in mortality, that is, their findings still need to be replicated.

Germany has high levels of alcohol consumption and ranks above the Organization for Economic Co‐operation and Development (OECD) average (OECD 2023) with certain federal states, for example, Mecklenburg‐Western Pomerania and Saxony‐Anhalt, showing particularly high numbers of alcohol‐attributable deaths (Schaller et al. 2022). However, none of the works on statistical interaction effects of SES and alcohol use in alcohol‐attributable mortality used data from individuals living in Germany. Moreover, some studies on differential effect did not differentiate between lifetime abstainers and current abstainers that used alcohol in the past (Christensen et al. 2017; Peña, Mäkelä, Laatikainen, et al. 2021), which might have led to bias (Stockwell et al. 2024). Finally, there are only two studies that investigated statistical interaction effects of SES and alcohol use on all‐cause mortality (Puka et al. 2022; Zhu et al. 2026) and only one study that examined the role of alcohol biomarkers (Peña, Mäkelä, Härkänen, et al. 2021).

In the present study, we thus investigate all‐cause mortality in participants of a cohort study from Western Pomerania, northeast Germany. We hypothesize that mortality in our sample is predicted by SES, self‐reported alcohol consumption, and alcohol biomarkers. Furthermore, we hypothesize that socioeconomic differences in mortality are attenuated but not fully explained by demographics, confounding risk factors, self‐reported alcohol use, and alcohol biomarkers. We assess whether biomarkers improve the predictive ability of our models beyond the effect of self‐reported alcohol use. Finally, we examine statistical interactions of SES and self‐reported alcohol consumption to investigate whether there is a differential effect.

## 2 Materials and Methods

### 2.1 Procedure and Participants

We used data of the baseline survey (1997–2001) of SHIP (Study of Health in Pomerania)‐START and information on participants' vital status from population registries. The study design of SHIP has been described in detail in Völzke et al. (2011, 2022). SHIP‐START was designed to assess prevalence and incidence of common risk factors, subclinical disorders, and clinical diseases, as well as to investigate associations between those. The study adhered to the ethical principles for medical research involving human participants outlined in the Declaration of Helsinki. The medical ethics committee of the University of Greifswald approved the study protocol, and oral and written informed consents were obtained from each of the study participants.

The study population for SHIP‐START was selected to be representative of the adult population (20–89 years) of Western Pomerania, a region in the northeast of Germany (Völzke et al. 2011). The two‐staged stratified cluster sample comprised 6265 eligible subjects. Of those, 4307 subjects (2192 female) participated in the baseline survey (response rate 68.6%), which was also used in this study. Participation included computer‐assisted personal interviews, self‐report questionnaires, and medical examinations.

### 2.2 Measures

#### 2.2.1 Outcome

All‐cause mortality from baseline until January 2023 was our outcome variable. In SHIP‐START, a mortality follow‐up is implemented annually using information on vital status from population registries (Völzke et al. 2011). For deceased persons, death certificates are requested from local health authorities.

#### 2.2.2 Exposures

Exposure variables in this study were SES, alcohol use, and alcohol biomarkers. For SES, net equivalent household income and years of education were used as proxy variables (Völzke et al. 2011). Net equivalent income was assessed through a self‐report questionnaire, divided by the square root of the household size (OECD 2013), and, similar to Probst et al. (2025), transformed into terciles, with the first tercile containing the lowest and the third tercile the highest incomes. Within the computer‐assisted interview, participants were asked to state their highest educational degree. The information on the highest educational degree was converted into the corresponding years of schooling and separated into three categories (less than 10, 10, or more than 10 years of schooling).

Participants completed a self‐report questionnaire on alcohol consumption. First, they were asked whether they had ever drunk an alcoholic drink. Participants who confirmed were asked whether they had drunk an alcoholic drink within the past 12 months. Participants who confirmed then answered a 3‐item quantity‐frequency questionnaire, covering the use of beer, wine/sparkling wine, and spirits in the last 30 days (Baumeister et al. 2005).

From this information, the number of drinks consumed in the past month was determined. By multiplying the number of drinks with the beverage‐specific average alcohol content, alcohol consumption in grams was calculated (Baumeister et al. 2005). In addition, drinking status (i.e., no alcoholic drink lifetime, no alcoholic drink in the past 12 months, and alcohol use in the past 12 months) was determined. Integrating these two variables, alcohol consumption was defined as either being a lifetime abstainer (no alcoholic drink lifetime), current abstainer (no alcoholic drink in the past 12 months, but before), or a participant that currently consumes alcohol. Following the classification of Peña, Mäkelä, Härkänen, et al. (2021), the final category is further divided into having low (0 to 83 g of ethanol/week), moderate (women: 84 to 168 g/week; men: 84 to 252 g/week), or high intake (women: more than 168 g/week; men: more than 252 g/week). Moreover, we defined HED as drinking more than five alcoholic drinks at least once in the past 30 days.

We included the set of biomarkers used in a recent study (Peña, Mäkelä, Härkänen, et al. 2021) and added mean corpuscular volume (MCV) based on relevant reviews (Gonzalo et al. 2014; Niemelä 2016; Tavakoli et al. 2011). GGT, ALT, AST, and CDT were determined from serum; MCV was determined from EDTA whole blood. Blood samples were taken from the arm vein with participants lying down. Vacutainers were used, and measurements were carried out in routine clinical laboratories in Greifswald and Stralsund.

#### 2.2.3 Confounders

In our main analyses, we controlled for age, sex, marital status, smoking, and BMI. Marital status and cigarette smoking were assessed within the computer‐assisted interview and defined as either being married, unmarried, divorced, or widowed and being a non‐smoker, former smoker, or current smoker, respectively. Weight and height were assessed by study personnel, and BMI was calculated as weight divided by height in meters squared (kg/m^2^).

In the sensitivity analyses, we controlled for health‐related quality of life and depression. Health‐related quality of life was operationalized using the short‐form health survey (SF‐12) including the physical component summary (pcs, range 0–100) and mental component summary (mcs, range 0–100) (Ware et al. 1996). For both summaries, higher values indicate better health. Possible depression was assessed using the two CID‐S screening questions on depression (Wittchen et al. 1999). Possible answers for both screening questions are “yes” and “no”. If either question was answered “yes,” the derived variable for possible depression was also coded “yes.”

A report on data quality for all variables included in our analyses was created using Stata (Schmidt and Schulze Kalthoff 2025), using the package dqrep (Schmidt 2025) for automated data quality assessments. dqrep is in routine use in SHIP for data quality monitoring.

### 2.3 Statistical Analyses

Prior to statistical analyses, values of GGT, ALT and AST were divided by 10 and values of MCV and CDT were divided by two, so that the estimates now represent changes per 10‐unit or 2‐unit increase in the biomarker level. The main and sensitivity analyses were carried out using Cox proportional hazards regression (CPHR). For the main analyses and similar to Peña, Mäkelä, Härkänen, et al. (2021), we computed five models to estimate the amount to which a possible social gradient in all‐cause mortality can be explained by the confounding risk factors, alcohol use and alcohol biomarkers. Model 1 included age, sex, income or education. Model 2 additionally controlled for marital status, smoking and BMI. In model 3, we added self‐reported alcohol consumption, and in models 4a–e GGT, MCV, ALT, AST, and CDT, respectively. Models 5a–e included both self‐reported alcohol use and one of the biomarkers. Descriptively comparing hazard ratios for participants with low income/education (compared to participants with high income/education) in model 1 with those in the following models allows one to determine the proportion of the social gradient in mortality that is attenuated by risk factors, alcohol use and biomarkers. We computed Harrel's *C* and tested whether including biomarkers in models 4a–e and 5a–e improved the predictive ability compared to model 3. Following Probst et al. (2025), we calculated the proportion of all‐cause mortality that can be explained by risk factors (model 1 versus model 2), self‐reported alcohol use (model 2 versus model 3) and alcohol biomarkers (model 3 versus models 5a–e) as $1-\frac{\ln\left({\mathrm{HR}}_{\text{adjusted model}}\right)}{\ln\left({\mathrm{HR}}_{\text{non}-\text{adjusted model}}\right)}$.

**FIGURE 1:**
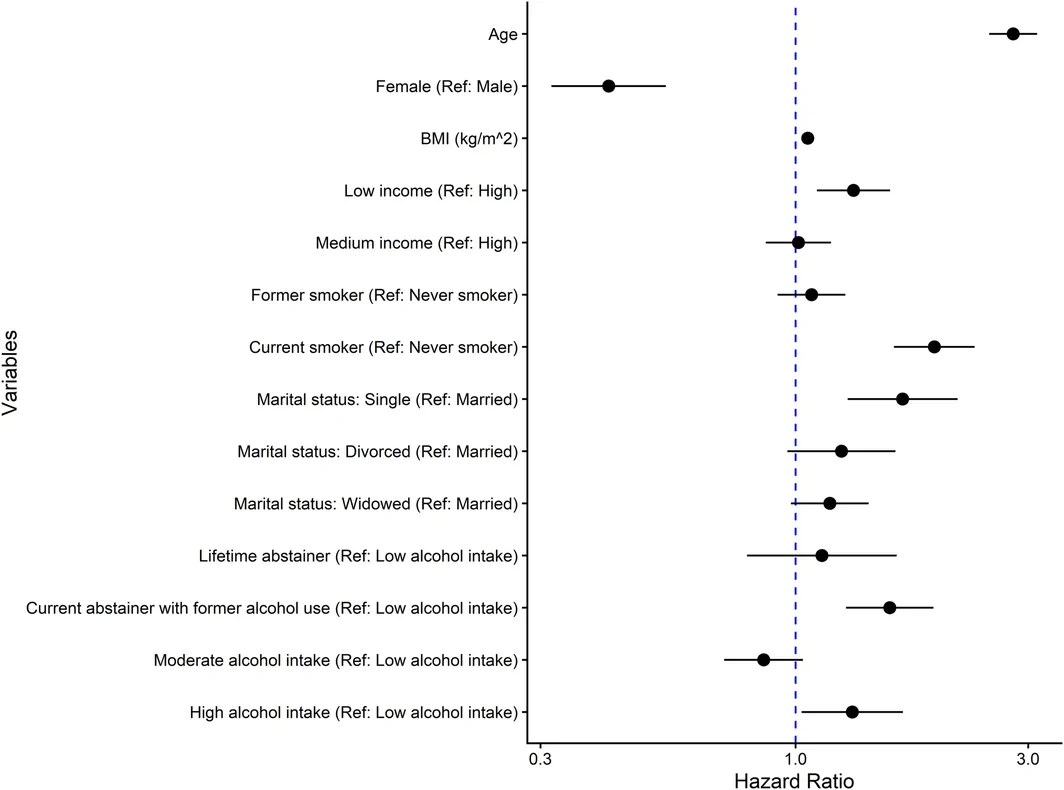
Hazard ratios for all‐cause mortality by income. Ref: Reference category. The figure illustrates the results from model 3. Points represent hazard ratios (HR) and horizontal bars represent 95% confidence intervals (CI). For age and sex, HR and CI for the first time interval are shown. Age was divided by 10 and BMI was divided by 2, so that the estimates now represent changes per 10‐ or 2‐unit increase.

**FIGURE 2:**
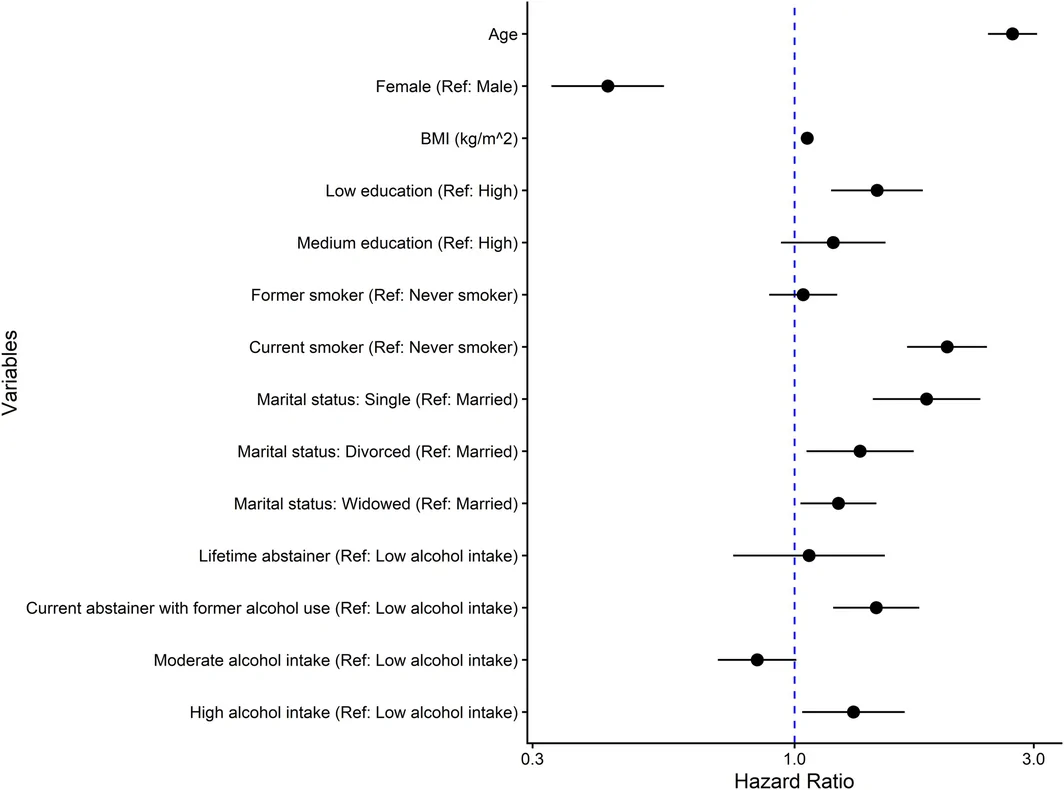
Hazard ratios for all‐cause mortality by education. Ref: Reference category. The figure illustrates the results from model 3. Points represent hazard ratios (HR) and horizontal bars represent 95% confidence intervals (CI). For age and sex, HR and CI for the first time interval are shown. Age was divided by 10 and BMI was divided by 2, so that the estimates now represent changes per 10‐ or 2‐unit increase.

We checked whether assumptions of CPHR were fulfilled in the final models, 5a–e. First, we tested for violations of the proportional hazards assumption by using both a test (Supplemental Material A, Table A) and the visual inspection of graphs of the scaled Schoenfeld residuals against transformed time for each exposure variable and confounder (Fox and Weisberg 2023; Harrell 2015). The variables age, sex and marital status did not meet the proportional hazards assumption (Supplemental Material A, Figures 1–5), that is, the effects of age, sex and marital status changed with time. To account for violations, as in a previous study (Peña, Mäkelä, Härkänen, et al. 2021), we split the data into three time intervals based on visual inspection of the graphs. The effects of the variables age and sex were modeled as time varying using these time intervals. In the model with time‐varying effects of age and sex, marital status met the proportional hazards assumption and its effect was therefore retained as time invariant.

Second, to test for nonlinearity in the relationship between the log hazard and the exposure variables and confounders, we used visual inspection of graphs of the martingale residuals from the final models against the continuous exposure variables and confounders (Fox and Weisberg 2023; Harrell 2015). Following visual inspection of the graphs, the variable BMI met the assumption of linearity, while the biomarker variables showed slight nonlinearity. We therefore repeated analyses with penalized splines for biomarker variables, with the degrees of freedom (df) specified as df = 0 to let the algorithm find the optimal degrees of freedom using the AIC criterion.

It has been shown that associations between alcohol consumption and all‐cause mortality differ between women and men (Zhao et al. 2023). We thus repeated both our descriptive and main analyses, stratified by sex, as a first set of sensitivity analyses.

As another set of sensitivity analyses, we reran the main analyses, but with net equivalent income as a non‐categorized variable. Prior to these analyses, we removed extreme values, that is, values that are located more than three times below the first or above the third quartile (Tukey's method). Again, to test for nonlinearity in the relationship between the log hazard and net equivalent income, we used visual inspection of graphs of the martingale residuals from the final model. The variable net equivalent income appeared to satisfy the assumption of linearity. Nevertheless, we assessed a potential nonlinear association between net equivalent income and all‐cause mortality using penalized splines.

In a recent review (Stockwell et al. 2024), typical sources of bias in alcohol research were identified, that is, usage of older cohorts, presence of participants with former and/or occasional alcohol use in abstainer reference groups, poor‐quality alcohol use measures, inclusion of individuals with pre‐existing health conditions and controlling for smoking status or SES. To address potential sources of these biases, we carried out the following additional sensitivity analyses using the final model 5a: (a) repetition of the analysis for the younger part of the cohort (aged < 55 at baseline), (b) exclusion of individuals with pre‐existing health conditions (pcs and/or mcs scores in SF12 < 40), (c) no control of smoking status, (d) no control of income/education. Additionally, we carried out further sensitivity analyses: (e) analyses using HED instead of alcohol use, (f) control of health‐related quality of life as pcs/mcs scores and (g) control of depressive symptoms.

In our statistical interaction analyses, we computed a model following Peña, Mäkelä, Laatikainen, et al. (2021), where we included age, sex, marital status and a product term of income/education with alcohol use to test for a statistical interaction effect. Firstly, as in previous studies (Peña, Mäkelä, Laatikainen, et al. 2021; Probst et al. 2025; Puka et al. 2022), we carried out the interaction analyses using Aalen's additive hazards regression (AAHR). AAHR allows one to directly include product terms to assess deviation from the additivity of effects (Rod et al. 2012). However, Kolmogorov–Smirnov and Cramer von Mises tests for time‐invariant effects revealed that except for age, all variables in our models were time invariant. Thus, by using constraints, all variables except for age were included as time invariant.

Secondly, as in a recent study (Probst et al. 2025), we repeated the interaction analyses using CPHR. In CPHR, product terms are used to evaluate deviations from multiplicativity or differences in relative effects in different subgroups (Rod et al. 2012). As in AAHR, we included a product term of income/education and alcohol use. We used likelihood ratio tests to compare the CPHR interaction model to a model including the same variables but no product term. Finally, for individuals who used alcohol in the past 12 months only, we carried out a sensitivity analysis where we included a product term of income and alcohol use in grams/week, both as z‐standardized continuous variables.

To simplify the interpretation of the results from the statistical interaction analyses, we reparametrized both hazard ratios (CPHR) and coefficients (AAHR) as differences to the reference group (i.e., individuals with low alcohol intake and high SES) by summing coefficients and multiplying hazard ratios for main and interaction effects from the model output. In AAHR, we used coefficients to calculate additional cases per 10,000 person‐years as suggested by Rod et al. (2012). As the underlying time scale were days (not years) in our study, coefficients were additionally multiplied by the average number of days per year.

For all statistical analysis, we computed 95% confidence intervals and *p*‐values where appropriate. *p*‐values below 5% were considered to indicate statistical significance. We used R version 4.3.1 for all statistical analyses.

## 3 Results

### 3.1 Sample Characteristics

Two different complete‐case datasets were built. Of the 4307 participants, 536 (12.4%) and 368 (8.5%) were excluded due to missing data for income or education, respectively, and any other study variable, resulting in final study populations of 3771 (dataset with income as exposure) and 3939 individuals (education dataset). For detailed information on missing data, see Supplemental Material A, Table B. We observed a total of 981 (26.0% of final sample) and 1047 (26.6%) deaths during the observation period. Sample characteristics of the final study populations are displayed in Table 1 (income dataset) and Supplemental Material A, Table C (education dataset). For information on missing data and descriptive statistics, stratified by sex, see Supplemental Material B, Tables A–C.

**TABLE 1:** Descriptive statistics on 3771 participants by income tercile.

	Full sample	Income (terciles)		
		First (lowest)	Second	Third (highest)
Total participants (*N*)	3771	1236	1309	1226
Deaths (% died)	26.0	22.9	29.3	25.7
Sex (% female)	51.0	53.6	54.7	44.6
Age (in years)	49.14 (16.34)	44.03 (16.32)	51.94 (16.66)	51.30 (14.77)
SF‐12				
PCS	48.17 (8.89)	48.58 (8.85)	47.30 (9.10)	48.68 (8.63)
MCS	51.89 (8.28)	50.72 (8.72)	52.31 (8.14)	52.60 (7.84)
BMI (kg/m^2^)	27.22 (4.76)	27.04 (5.07)	27.57 (4.82)	27.01 (4.34)
Smoking				
Never smoker (%)	36.0	31.7	39.3	36.7
Former smoker (%)	33.5	25.6	34.1	40.9
Current smoker (%)	30.5	42.7	26.7	22.4
Alcohol use				
Lifetime abstainers (%)	2.6	3.7	2.9	1.1
Current abstainers (%)	5.5	7.3	5.7	3.6
Low intake (%)	61.3	61.5	63.4	58.9
Moderate intake (%)	21.3	17.3	20.9	25.8
High intake (%)	9.3	10.2	7.1	10.6
Biomarker				
GGT (U/L)	35.38 (78.03)	38.13 (95.62)	31.96 (58.78)	36.27 (76.24)
ALT (U/L)	28.54 (20.48)	29.34 (20.77)	27.32 (19.65)	29.03 (21.00)
AST (U/L)	22.21 (13.71)	22.70 (13.69)	21.77 (15.05)	22.18 (12.14)
MCV (fl)	90.10 (4.51)	90.10 (4.83)	90.05 (4.25)	90.14 (4.45)
CDT (%)	4.81 (1.62)	4.91 (1.85)	4.76 (1.56)	4.76 (1.41)

*Note:* Numbers display absolute values (*N*), percentages (*%*) or mean (standard deviation in brackets).

Abbreviations: ALT, alanine transaminase; AST, aspartate transaminase; BMI, body mass index; CDT, carbohydrate‐deficient transferrin; GGT, gamma‐glutamyltransferase; MCS, mental component summary; MCV, mean corpuscular volume; PCS, physical component summary.

### 3.2 Main Analyses

#### 3.2.1 Using Income as an Exposure

The results of our main analyses are presented in Table 2. In model 1, age, sex and income were associated with all‐cause mortality. Compared with men, women had a lower hazard ratio for mortality in the first time interval (HR = 0.43, CI = 0.34, 0.56), but higher hazard ratios in the second (HR = 1.15, CI = 0.84, 1.58) and third (HR = 1.58, CI = 1.10, 2.26) time interval. Age was associated with a 10% increased hazard per year (HR = 1.10, CI = 1.09, 1.11) in the first interval. In the second (HR = 1.01, CI = 1.00, 1.03) and third interval (HR = 1.02, CI = 1.00, 1.04), 1% and 2% increased hazards per year were found. Individuals in the lowest (HR = 1.59, CI = 1.35, 1.87) and in the middle income tertile (HR = 1.11, CI = 0.95, 1.29) had higher hazard ratios than individuals in the highest income tertile. In model 2, marital status, smoking and BMI were associated with mortality. Compared with married individuals, divorced (HR = 1.23, CI = 0.96, 1.59), single (HR = 1.70, CI = 1.31, 2.21) and widowed (HR = 1.20, CI = 1.00, 1.45) participants had higher hazard ratios. Former (HR = 1.06, CI = 0.91, 1.25) and current smokers (HR = 1.93, CI = 1.60, 2.33) had higher hazard ratios than non‐smokers. Higher BMI was associated with a 3% increased hazard per unit (HR = 1.03, CI = 1.02, 1.05). In model 3, lifetime abstainers (HR = 1.13, CI = 0.80, 1.61), current abstainers (HR = 1.56, CI = 1.27, 1.92) and participants with high alcohol intake (HR = 1.31, CI = 1.03, 1.66) had higher hazard ratios for mortality compared to participants with low alcohol intake. Participants with moderate alcohol intake (HR = 0.86, CI = 0.71, 1.04) had lower hazard ratios. The hazard ratios from model 3 are also presented in Figure 1.

**TABLE 2:** Risk of death according to income (tercile) in cox regression models.

	Model 1		Model 2		Model 3		Model 5a	
	Harrel's *C*: 0.855		Harrel's *C*: 0.861		Harrel's *C*: 0.863		Harrel's *C*: 0.865	
	HR	95% CI	HR	95% CI	HR	95% CI	HR	95% CI
Age (*t* = 1)	1.10	1.09, 1.11	1.11	1.10, 1.12	1.11	1.10, 1.12	1.11	1.10, 1.12
Age (*t* = 2)	1.01	1.00, 1.03	1.02	1.00, 1.03	1.02	1.00, 1.03	1.02	1.00, 1.03
Age (*t* = 3)	1.02	1.00, 1.04	1.02	1.00, 1.04	1.02	1.00, 1.04	1.02	1.00, 1.04
Sex								
Male	Reference		Reference		Reference		Reference	
Female (*t* = 1)	0.43	0.34, 0.56	0.41	0.32, 0.54	0.41	0.32, 0.54	0.43	0.33, 0.57
Female (*t* = 2)	1.15	0.84, 1.58	1.14	0.83, 1.56	1.14	0.83, 1.55	1.12	0.82, 1.54
Female (*t* = 3)	1.58	1.10, 2.26	1.57	1.09, 2.25	1.56	1.09, 2.24	1.53	1.07, 2.19
Income (tercile)								
1 (lowest)	1.59	1.35, 1.87	1.37	1.15, 1.62	1.31	1.11, 1.56	1.32	1.11, 1.57
2	1.11	0.95, 1.29	1.03	0.89, 1.20	1.01	0.87, 1.18	1.03	0.88, 1.20
3 (highest)	Reference		Reference		Reference		Reference	
Marital status								
Married			Reference		Reference		Reference	
Divorced			1.23	0.96, 1.59	1.24	0.96, 1.60	1.24	0.96, 1.60
Single			1.70	1.31, 2.21	1.66	1.28, 2.15	1.60	1.23, 2.08
Widowed			1.20	1.00, 1.45	1.18	0.98, 1.41	1.15	0.95, 1.38
Smoking								
Never smoker			Reference		Reference		Reference	
Former smoker			1.06	0.91, 1.25	1.08	0.92, 1.27	1.09	0.93, 1.28
Current smoker			1.93	1.60, 2.33	1.93	1.59, 2.33	1.90	1.57, 2.30
BMI (kg/m^2^)			1.03	1.02, 1.05	1.03	1.01, 1.04	1.03	1.01, 1.04
Alcohol use								
Lifetime abstainers					1.13	0.80, 1.61	1.14	0.80, 1.62
Current abstainers					1.56	1.27, 1.92	1.57	1.28, 1.93
Low intake					Reference		Reference	
Moderate intake					0.86	0.71, 1.04	0.85	0.70, 1.02
High intake					1.31	1.03, 1.66	1.22	0.95, 1.55
GGT							1.01	1.01, 1.02

*Note:* Models with income include 3771 participants. Model 1 is adjusted for age, sex, and income; model 2 is model 1 + adjustment for marital status, smoking, and BMI; model 3 is model 2 + adjustment for alcohol use; model 5a is model 3 + adjustment for GGT. Values of GGT were divided by 10, so that the estimates now represent changes per 10‐unit increase.

Abbreviations: BMI, body mass index; CI, confidence interval; GGT, gamma‐glutamyltransferase; HR, hazard ratio.

When adding alcohol biomarkers instead of self‐reported alcohol use in models 4a–e (see Supplemental Material A, Table D), we observed positive associations of GGT (HR = 1.01, CI = 1.01, 1.02), MCV (HR = 1.04, CI = 1.01, 1.08), ALAT (HR = 1.03, CI = 0.99, 1.07), AST (HR = 1.09, CI = 1.05, 1.13), and CDT (HR = 1.07, CI = 0.98, 1.16) with mortality.

In our final models, 5a–e, with both self‐reported alcohol use and alcohol biomarkers, the direction of associations between self‐reported alcohol use and mortality remained unchanged (see Table 2 and Supplemental Material A, Table E).

Compared to model 3 with self‐reported alcohol use, the predictive ability measured by Harrel's *C* was statistically significantly reduced in models with ALT and CDT (4c, 4e). Moreover, the predictive ability was statistically significantly improved in model 5a with both self‐reported alcohol use and GGT compared to model 3. For models 4a, b, d and 5b–e, there were no significant differences in Harrel's *C* compared to model 3 (see Supplemental Material A, Table I).

To evaluate nonlinear effects of the biomarkers, we included penalized splines into models 4a–e and 5a–e (Supplemental Material A, Tables J and K). For GGT, MCV, AST, ALT, and CDT, models 4a–e and 5a–e revealed significant linear and nonlinear associations. The spline functions from these models are displayed in the Supplemental Material A, Figures 7 and 8.

#### 3.2.2 Using Education as an Exposure

The Spearman's rank correlation between income and education was *ρ* = 0.19 (*p* < 0.001) in our full sample, that is, a significant but rather weak correlation, suggesting that different dimensions of SES were recorded. We thus repeated our main analyses using education instead of income as a secondary exposure. The results from these analyses are reported in Supplemental Material A, Tables F–H.

In brief, the results were similar to those from models using income as a proxy for SES. More precisely, in model 1, individuals with less than 10 years of education (HR = 1.62, CI = 1.32, 2.00) or with exactly 10 years of education (HR = 1.26, CI = 0.99, 1.59) had higher hazard ratios for mortality than those with more than 10 years of education. We also found associations of age and sex (model 1) and marital status, smoking and BMI (model 2) with mortality. Compared to men, women had a lower hazard ratio (HR = 0.46, CI = 0.36, 0.59) in the first time interval, but higher hazard ratios in the second (HR = 1.08, CI = 0.80, 1.46) and third (HR = 1.45, CI = 1.02, 2.05) time interval. Age was associated with 10%, 1% and 2% increased hazards per year in the first (HR = 1.10, CI = 1.08, 1.11), second (HR = 1.01, CI = 1.00, 1.03) and third (HR = 1.02, CI = 1.00, 1.03) time interval.

Compared to married individuals, divorced (HR = 1.35, CI = 1.06, 1.73), single (HR = 1.89, CI = 1.48, 2.42), and widowed (HR = 1.23, CI = 1.04, 1.48) participants had higher hazard ratios. Both former (HR = 1.03, CI = 0.88, 1.20) and current smokers (HR = 2.02, CI = 1.68, 2.42) had higher hazard ratios than non‐smokers. Higher BMI was associated with a 3% increased hazard per unit (HR = 1.03, CI = 1.02, 1.05). Lifetime abstainers (HR = 1.07, CI = 0.75, 1.51), current abstainers (HR = 1.46, CI = 1.19, 1.77), and individuals with high alcohol intake (HR = 1.31, CI = 1.04, 1.66) had higher hazard ratios for mortality compared to individuals with low alcohol intake (model 3). Individuals with moderate intake had a lower hazard ratio (HR = 0.84, CI = 0.70, 1.01). The hazard ratios from model 3 are displayed in Figure 2.

When adding alcohol biomarkers instead of self‐reported alcohol use in models 4a–e (see Supplemental Material A, Table G), we observed positive associations of GGT (HR = 1.01, CI = 1.01, 1.02), MCV (HR = 1.04, CI = 1.01, 1.07), ALAT (HR = 1.02, CI = 0.98, 1.06), AST (HR = 1.09, CI = 1.05, 1.13), and CDT (HR = 1.07, CI = 0.99, 1.16) with mortality.

In the models with both self‐reported alcohol use and alcohol biomarkers (model 5a–e), the direction of associations between self‐reported alcohol use and mortality remained unchanged.

### 3.3 Sex‐Stratified Analyses

The full results of the sex‐stratified analyses using income as a predictor variable are displayed in Supplemental Material B, Tables D–I. Among women, we found the following associations with mortality: In model 1, age was associated with a 13% increased hazard per year (HR = 1.13, CI = 1.11, 1.16) in the first time interval. In the second (HR = 1.00, CI = 0.97, 1.03) and third time interval (HR = 1.00, CI = 0.97, 1.03), changes in the hazard per year were below < 1%. Women in the lowest (HR = 1.17, CI = 0.88, 1.54) and in the middle income tercile (HR = 1.08, CI = 0.84, 1.39) had higher hazard ratios than women in the highest income tercile.

In model 2, compared to married female individuals, divorced (HR = 0.84, CI = 0.58, 1.23) and widowed female individuals (HR = 0.96, CI = 0.74, 1.25) displayed lower hazard ratios for mortality. Single female participants, however, displayed a higher hazard ratio for mortality (HR = 1.14, CI = 0.77, 1.68). Moreover, compared to non‐smokers, both former (HR = 1.18, CI = 0.92, 1.52) and current smokers (HR = 1.56, CI = 1.14, 2.14) had higher hazard ratios, and higher BMI was associated with a 3% increased hazard per unit (HR = 1.03, CI = 1.01, 1.05).

In model 3, lifetime abstainers (HR = 1.26, CI = 0.84, 1.91), current abstainers (HR = 1.57, CI = 1.14, 2.16), and women with high alcohol intake (HR = 1.30, CI = 0.66, 2.55) had higher hazard ratios compared to individuals with low intake. Women with moderate intake had a lower hazard (HR = 0.74, CI = 0.43, 1.28). In models 4a, c, and d, GGT (HR = 1.04, CI = 1.02, 1.06), ALAT (HR = 1.02, CI = 0.96, 1.10), and AST (HR = 1.03, CI = 0.93, 1.13) were associated with a 4%, 2%, and 3% increased hazard per 10‐unit increase. In models 4b and e, MCV (HR = 0.99, CI = 0.94, 1.04) and CDT (HR = 0.93, CI = 0.80, 1.09) were associated with a 1% and 7% decreased hazard per 2‐unit increase. In the models with both self‐reported alcohol use and alcohol biomarkers (model 5a–e), the direction of associations with mortality remained unchanged.

The following associations with mortality were observed among men: In model 1, age was associated with 9%, 2% and 2% increased hazards per year in the first (HR = 1.09, CI = 1.08, 1.11), second (HR = 1.02, CI = 1.00, 1.04) and third (HR = 1.02, CI = 1.00, 1.05) time interval. Men in the lowest (HR = 1.87, CI = 1.53, 2.28) and in the middle income tercile (HR = 1.07, CI = 0.89, 1.30) had higher hazard ratios than men in the highest income tercile.

In model 2, compared to married male individuals, divorced (HR = 1.83, CI = 1.29, 2.60), single (HR = 2.31, CI = 1.59, 3.36) and widowed male individuals (HR = 1.25, CI = 0.95, 1.66) displayed higher hazard ratios for mortality. As with women, both former (HR = 1.16, CI = 0.93, 1.45) and current smokers (HR = 2.30, CI = 1.77, 2.99) exhibited higher hazard ratios compared to non‐smokers and higher BMI was associated with a 4% increased hazard per unit (HR = 1.04, CI = 1.01, 1.06) among men. In model 3, both current abstainers that used alcohol in the past (HR = 1.54, CI = 1.17, 2.02) and men with high alcohol intake (HR = 1.26, CI = 0.97, 1.63) had higher hazard ratios compared to men with low alcohol intake. Lifetime abstainers (HR = 0.95, CI = 0.47, 1.93) and individuals with moderate intake (HR = 0.89, CI = 0.73, 1.09) had lower hazard ratios. In models 4a ‐e, GGT (HR = 1.01, CI = 1.01, 1.02), ALAT (HR = 1.03, CI = 0.98, 1.08) and AST (HR = 1.11, CI = 1.07, 1.15) were associated with a 1%, 3% and 11% increased hazard per 10‐unit increase. MCV (HR = 1.09, CI = 1.04, 1.13) and CDT (HR = 1.11, CI = 1.01, 1.22) were associated with a 9% and 11% increased hazard per 2‐unit increase. As among women, the direction of associations with mortality remained unchanged in the models with both self‐reported alcohol use and alcohol biomarkers (models 5a–e).

The full results of the sex‐stratified analyses using education instead of income as a predictor are displayed in Supplemental Material B, Tables J–O.

### 3.4 Sensitivity Analyses

The results of our sensitivity analyses are displayed in Tables L‐P, Supplemental Material A. In model 1, net equivalent income was associated with a 5% decreased hazard per 100€ (HR = 0.95, 95% CI = 0.94, 0.97). Controlling for marital status, BMI, smoking, alcohol use, and GGT in model 5a, net equivalent income was still associated with a 2% decreased hazard per 100€ (HR = 0.97, 95% CI = 0.95, 0.99).

When we reran model 5a with penalized splines for net equivalent income, the analysis revealed a significant linear association and a non‐significant nonlinear association. The spline function from model 5a with penalized splines for net equivalent income is displayed in the Supplemental Material A, Figure 6.

A comparison of the results from our final models 5a both with and without penalized splines for net equivalent income is displayed in Supplemental Material A, Table O. The likelihood ratio test suggests a significant difference in fit between models (*χ* ^2^(0.01) = 0.02, *p* = 0.022).

The results of the further sensitivity analyses are reported in Table P, Supplemental Material A. To sum up, in the analysis restricted to individuals under the age of 55, lifetime (HR = 0.35, CI = 0.05, 2.54) and current abstainers (HR = 0.84, CI = 0.38, 1.85) exhibited a 65% and 16% lower hazard compared to participants with low alcohol intake. However, in all other sensitivity analyses, both groups displayed higher mortality. Participants with moderate alcohol intake had lower (up to 25%), while participants with high alcohol intake had higher (up to 41%) hazard ratios for mortality. In our analysis using HED instead of alcohol use (e), HED (HR = 0.94, CI = 0.80, 1.11) was associated with a 6% reduced mortality hazard.

### 3.5 Interaction Analyses

Firstly, we carried out interaction analysis using AAHR. Using income as an exposure variable, compared to the reference group (i.e., individuals with low alcohol intake from the high‐income group), we found lower mortality in participants with moderate and high intake from the medium‐income group (see Table 3). All other groups displayed higher mortality.

**TABLE 3:** Risk of death according to interactions of income (tercile) and alcohol use in Cox regression models and Aalen additive models.

	*N*	Cox proportional hazards regression				Aalen additive hazards regression					
		Model output		Reparametrization		Model output			Reparametrization		
		HR	95% CI	HR	95% CI	Coefficient	95% CI	Additional cases per 10,000‐person‐years	Coefficient	95% CI	Additional cases per 10,000‐person‐years
Income (terciles)											
1 (lowest)	1236	1.27	1.02, 1.58			1.09e‐05	3.45e‐06, 1.83e‐05	39.81			
2	1309	1.19	0.98, 1.44			8.33e‐06	4.71e‐07, 1.62e‐05	30.43			
3 (highest)	1226	Reference									
Alcohol use											
Lifetime abstainers	98	0.46	0.11, 1.84			‐2.82e‐05	‐5.68e‐05, 4.15e‐07	−103.00			
Current abstainers	209	2.22	1.51, 3.28			7.71e‐05	2.57e‐05, 1.28e‐04	281.62			
Low intake	2312	Reference									
Moderate intake	803	0.84	0.62, 1.13			−1.70e‐05	−2.58e‐05, −8.16e‐06	−62.10			
High intake	349	1.53	1.05, 2.23			1.59e‐06	−1.19e‐05, 1.51e‐05	5.81			
Interactions											
1, lifetime abstainer	46	3.07	0.69, 13.65	1.78	1.05, 3.02	2.70e‐05	−1.14e‐05, 6.54e‐05	98.62	9.73e‐06	−2.214e‐05, 4.161e‐05	35.56
2, lifetime abstainer	38	2.46	0.56, 10.86	1.33	0.80, 2.23	2.33e‐05	−2.06e‐04, 6.72e‐05	85.10	3.48e‐06	−2.078e‐05, 2.774e‐05	12.71
1, current abstainer	90	0.97	0.57, 1.64	2.73	1.92, 3.90	−5.03e‐05	−1.07e‐04, 5.95e‐06	−183.72	3.774e‐05	−3.134e‐05, 0.00010682	137.84
2, current abstainer	75	0.51	0.31, 0.84	1.34	0.96, 1.88	−4.38e‐05	−1.05e‐04, 1.75e‐05	−159.98	4.167e‐05	−2.322e‐05, 0.0001065719	152.22
1, moderate intake	214	1.71	1.09, 2.68	1.83	1.30, 2.58	1.50e‐05	2.10e‐06, 2.79e‐05	54.79	8.91e‐06	‐5e‐08, 1.787e‐05	32.55
2, moderate intake	273	0.83	0.55, 1.25	0.82	0.61, 1.12	2.51e‐06	−1.01e‐05, 1.52e‐05	9.17	−6.2e‐06	−1.73e‐05, 4.89e‐06	−22.65
1, high intake	126	1.17	0.69, 1.98	2.28	1.55, 3.33	−4.27e‐07	−1.97e‐05, 1.89e‐05	−1.56	1.21e‐05	−1.87e‐06, 2.608e‐05	44.20
2, high intake	93	0.60	0.33, 1.10	1.09	0.67, 1.78	−1.65e‐05	−3.58e‐05, 2.81e‐06	−60.27	−6.62e‐06	−2.119e‐05, 7.95e‐06	−24.18
3, low intake	722	Reference									

*Note:* Models are adjusted for age, sex, and marital status. To simplify the interpretation of interaction effects, we reparametrized HR and coefficients from the model outputs, that is, products of HR of respective main and interaction effects (CPHR) and sums of coefficients of respective main and interaction effects (AAHR) were computed.

Abbreviations: CI, confidence interval; HR, hazard ratio.

Secondly, we repeated interaction analyses with CPHR. Using income as an exposure variable, compared to the reference group, we found lower mortality in participants with moderate alcohol intake from the medium‐income group (see Table 3). All other groups displayed higher mortality. The likelihood ratio test suggests that the CPHR model including a product term fits the data better (*χ* ^2^(8) = 22.80, *p* = 0.004). Our sensitivity analyses for individuals who used alcohol in the past 12 months revealed a statistically significant interaction effect of income and alcohol use in grams/day (HR = 0.93, CI = 0.87, 1.00, *p* = 0.049). The results from interaction analyses using education as an exposure variable are reported in Supplemental Material A, Table L.

## 4 Discussion

We found a social gradient in all‐cause mortality of individuals from Western Pomerania, northeast Germany. Higher mortality in low compared to high SES individuals was reduced but remained substantial in most analyses when controlling for demographics, confounding risk factors, self‐reported alcohol use, and alcohol biomarkers. Current abstainers with past alcohol use and individuals with current high alcohol intake displayed 56% and 31% higher mortality compared to those with low alcohol intake, and all biomarkers were associated with mortality. In most of the sensitivity analyses, our findings remained largely unchanged. In interaction analyses, we found some evidence for differential associations of alcohol use by SES.

Individuals with low income and low education faced 59% and 62% higher all‐cause mortality compared to those with high income and high education (controlled for age and sex, model 1), respectively. Of this social gradient, 48.5% (income) and 20.2% (education) were explained by marital status, smoking, and BMI (model 2). After controlling for these risk factors, individuals with low income and low education still faced 37% and 49% higher mortality. Of this social gradient, 14.0% and 6.0% were explained by alcohol use (model 3). This is within the range of estimates (−3% to 17%) from previous studies on all‐cause mortality (Probst et al. 2020; Puka et al. 2022). With estimates of only 0.6% or 0.3% (results not reported), the explanatory value of heavy episodic drinking (HED) is much lower in our sample than estimates of 24 to 27% in a previous study (Sydén and Landberg 2017). However, comparability of studies is limited due to different operationalizations of HED and samples from different countries (Stockholm County, Sweden, and Western Pomerania, Germany) which may contribute to differences in estimates.

We found higher mortality in participants with high alcohol intake, a finding that fits well with the existing literature (Zhao et al. 2023) and emphasizes harmful alcohol effects. Higher hazard ratios in participants with high compared to those with low alcohol intake were present across models in main analyses (up to 31%), in sensitivity analyses (up to 41%), and for both women (up to 47%) and men (up to 27%).

Moreover, we found higher mortality in lifetime abstainers (up to 16% in main analyses) and lower mortality in participants with moderate alcohol intake (up to 18% in main analyses), both compared to participants with low alcohol use. It is now well known that previously claimed health‐promoting effects of low alcohol use are largely attributable to bias (Stockwell et al. 2024). However, even when trying to avoid known sources of bias (e.g., by controlling for health‐related quality of life), the direction of these differences persisted in most of our sensitivity analyses. Only when we excluded individuals aged ≥ 55 years, we found the expected health benefits, that is, 65% reduced mortality, in lifetime abstainers. This corresponds to the analyses by Stockwell et al. (2024), who found that in higher quality studies on cohorts aged < 55 years at baseline, hazard ratios were higher in participants with low alcohol intake compared to abstainers. Lower mortality in participants with moderate alcohol intake was still observed in this subsample in our study, though. One explanation might be that we have not been able to sufficiently control for health status or illnesses. Thus, these might equalize the actual health benefits of low compared to moderate alcohol intake in our study. Furthermore, the described variations should be interpreted with caution, especially since the corresponding confidence intervals crossed 1.

We found positive associations of biomarkers with all‐cause mortality when controlling for demographics, confounding risk factors and self‐reported alcohol use in our main and sensitivity analyses. In line with previous findings (Yoon et al. 2016; Yuwaki et al. 2019), GGT, MCV, ALT, and AST showed positive associations with mortality. Moreover, CDT was linked to elevated mortality in our study.

Nevertheless, only GGT improved the predictive ability of our model. In contrast, the only previous study on this showed that using biomarkers in addition to self‐reported alcohol use increased the predictive ability for GGT, ALT, AST, and CDT (Peña, Mäkelä, Härkänen, et al. 2021). However, Peña, Mäkelä, Härkänen, et al. (2021) did not run models with only ALT, AST, or CDT, but combined all of them with GGT. Even more importantly, they examined associations with alcohol‐attributable mortality while all‐cause mortality was the outcome variable in our study, which might explain why the role of biomarkers is smaller in our study. Only 1.3 or 1.4% of the social gradient was explained by the alcohol biomarker GGT (after controlling for self‐reported alcohol use). We thus do not provide evidence of measurement errors in self‐reported alcohol use playing a major role in mortality inequality.

In our study, associations of demographics and alcohol use with mortality differed between women and men. First, mortality differences between individuals with the lowest and highest incomes were markedly more pronounced among men than among women. This is consistent with another study that also found stronger income gradients in mortality among men in Germany (Geyer et al. 2019).

Second, divorced and widowed women displayed lower while divorced and widowed men faced higher mortality. Both single women and men were found to have higher mortality, but this was more marked in men than women. Our findings point in a similar direction as those of a meta‐analysis that demonstrated that increased mortality associated with being non‐married is lower for women compared to men (Wang et al. 2020). Particularly among women, however, the findings of our study exhibit substantial uncertainty.

Third, female lifetime abstainers exhibited increased mortality compared to individuals with low alcohol intake. Male lifetime abstainers, however, were found to show lower mortality. Notably, confidence intervals of the respective hazard ratios included 1 for both women and men. Consequently, these results are characterized by high uncertainty and should be validated by further studies prior to a comprehensive interpretation.

Both, in analyses using AAHR and CPHR, we found indications of statistical interaction effects. Compared to individuals with low alcohol intake and high education, for example, individuals with high alcohol intake and low education displayed higher mortality, with 76.25 additional cases per 10,000 person‐years, respectively. As in previous studies (Christensen et al. 2017; Peña, Mäkelä, Laatikainen, et al. 2021; Probst et al. 2025; Smith et al. 2024), a differential effect of alcohol consumption by SES is thus generally supported. However, small subgroup counts clearly limit the certainty of the results from our statistical interaction analysis and impede substantive conclusions. To investigate whether there is a differential effect in Germany, future studies should perform interaction analyses in larger samples, for example, the German National Cohort (NAKO) (Peters et al. 2022), when a sufficient observation period has been reached.

### 4.1 Strengths and Limitations

This study comes with certain strengths and limitations. First, this is one of few studies to adjust for biomarkers of chronic alcohol consumption. Beyond that, our study is characterized by a long observation period of more than 20 years, with a mean observation time of more than 18 years (as compared to, respectively, 8.8 and 13.5 years in Puka et al. (2022) and Smith et al. (2024)). Finally, while some previous studies exclusively used education as an SES proxy (Christensen et al. 2017; Puka et al. 2022), we used income as a primary and education as a secondary SES proxy.

A limitation of our work is the much smaller sample size compared to similar studies (Christensen et al. 2017; Peña, Mäkelä, Härkänen, et al. 2021; Peña, Mäkelä, Laatikainen, et al. 2021; Puka et al. 2022; Smith et al. 2024), which increases uncertainty on the interpretations of our findings, particularly with regards to interaction effects. A corresponding limitation may be seen in the large number of hypotheses tested despite the relatively small sample size. Consequently, our findings require validation in future studies. As Boyd et al. (2023) noted as a limitation in comparable studies, we also had limited information on drinking history and used baseline alcohol use as a proxy. This approach risks classifying individuals as a lifetime or current abstainer or having a low alcohol intake who may have had a high intake in their drinking history, leading to underestimation of the association between alcohol consumption and mortality. In pursuit of transparent science, we made the underlying data quality assessment report publicly available. While these assessments did not reveal major issues, the temporal variability of laboratory markers may indicate some batch effects. This heterogeneity may have attenuated the findings.

Previous analyses of SHIP‐START data indicate a high prevalence of illnesses for the population in Western Pomerania, for example, gallstone disease, hepatic steatosis and hypertension (Meisinger et al. 2006; Völzke et al. 2011, 2005). Thus, it is very likely that individuals in our sample have reduced their alcohol use due to illness so that effects of (previous) alcohol consumption are concealed.

Finally, as explained in detail by Rübenach (2007), alcohol mortality is likely underestimated when using data from German population registries. We therefore decided to analyze associations of alcohol use and all‐cause mortality instead of alcohol mortality in our study.

## 5 Conclusions

In our study, low SES individuals, current abstainers that consumed alcohol in the past and participants with high alcohol intake displayed increased all‐cause mortality. Participants with moderate alcohol intake showed lower mortality. In most analyses, alcohol biomarkers were positively associated with mortality, that is, the higher the values of the respective biomarker, the more likely the participants' death was during the study period. The social gradient in mortality was attenuated—but not fully explained—by risk factors, self‐reported alcohol use and biomarkers. There was evidence of statistical interaction effects of alcohol use by income. A comparatively small sample size represents a major limitation of our study.

## Funding

SHIP is part of the Community Medicine Research net of the University of Greifswald, Germany, which is funded by the Federal Ministry of Education and Research (grants no. 01ZZ9603, 01ZZ0103, and 01ZZ0403), the Ministry of Cultural Affairs as well as the Social Ministry of the Federal State of Mecklenburg‐West Pomerania, and the network ‘Greifswald Approach to Individualized Medicine (GANI_MED)’ funded by the Federal Ministry of Education and Research (grant 03IS2061A).

## Conflicts of Interest

H.J.G. has received travel grants and speakers' honoraria from Neuraxpharm, Servier, Indorsia, and Janssen Cilag. All authors have confirmed to have no conflicts of interest with respect to this manuscript.

## Supporting information


**Table A.** Test for violations of the proportional hazards assumption in models 5a using either income or education as SES proxy.
**Table B.** Frequency of missing data.
**Table C.** Descriptive statistics on 4307 participants (dataset including missings) and 3939 participants by education group.
**Table D.** Risk of death according to income (tercile) in cox regression models 4a–4e.
**Table E.** Risk of death according to income (tercile) in cox regression models 5b–5e.
**Table F.** Risk of death according to education in cox regression models 1, 2, 3, and 5a.
**Table G.** Risk of death according to education in cox regression models 4a–4e.
**Table H.** Risk of death according to education in cox regression models 5b–5e.
**Table I.** Predictive validity in cox regression models including income.
**Table J.** Risk of death according to alcohol biomarkers in cox regression models 4a–4e with penalized splines.
**Table K.** Risk of death according to alcohol biomarkers in cox regression models 5a–5e with penalized splines.
**Table L.** Risk of death according to net equivalent income in cox regression models 1, 2, 3, and 5a.
**Table M.** Risk of death according to net equivalent income in cox regression models 4a–4e.
**Table N.** Risk of death according to net equivalent income in cox regression models 5b–5e.
**Table O.** Comparison of models 5a with and without penalized spline for incomes.
**Table P.** Risk of death according to alcohol use in sensitivity analyses using cox regression models 3 with age and sex as time–varying variables and income (tercile) as SES proxy.
**Table Q.** Risk of death according to interactions of education and alcohol use in cox regression models and Aalen additive models.

**Figure 1.** Graph of the scaled schoenfeld residuals for variable sex against transformed time in model 5a using income as SES proxy.
**Figure 2.** Graph of the scaled schoenfeld residuals for variable age against transformed time in model 5a using income as SES proxy.
**Figure 3.** Graph of the scaled schoenfeld residuals for variable marital status against transformed time in model 5a using income as SES proxy.
**Figure 4.** Graph of the scaled schoenfeld residuals for variable sex against transformed time in model 5a using education as SES proxy.
**Figure 5.** Graph of the scaled schoenfeld residuals for variable age against transformed time in model 5a using education as SES proxy.
**Figure 6.** Plot illustrating risk of death according to net equivalent income in cox regression model 5a with penalized splines.
**Figure 7.** Plots illustrating risk of death according to alcohol biomarkers in cox regression models 4a–4e with penalized splines.
**Figure 8.** Plots illustrating risk of death according to alcohol biomarkers in cox regression models 5a 5e with penalized splines.

**Table A.** Frequency of missing data.
**Table B.** Descriptive statistics by income tercile, stratified by sex.
**Table C.** Descriptive statistics by education group, stratified by sex.
**Table D.** Risk of death according to income (tercile) in cox regression models 1, 2, 3, and 5a for women.
**Table E.** Risk of death according to income (tercile) in cox regression models 4a–4e for women.
**Table F.** Risk of death according to income (tercile) in cox regression models 5b–5e for women.
**Table G.** Risk of death according to income (tercile) in cox regression models 1, 2, 3, and 5a for men.
**Table H.** Risk of death according to income (tercile) in cox regression models 4a–4e for men.
**Table I.** Risk of death according to income (tercile) in cox regression models 5b–5e for men.
**Table J.** Risk of death according to education in cox regression models 1, 2, 3, and 5a for women.
**Table K.** Risk of death according to education in cox regression models 4a–4e for women.
**Table L.** Risk of death according to education in cox regression models 5b–5e for women.
**Table M.** Risk of death according to education in cox regression models 1, 2, 3, and 5a for men.
**Table N.** Risk of death according to education in cox regression models 4a–4e for men.
**Table O.** Risk of death according to education in cox regression models 5b–5e for men.

## Data Availability

Data from the ‘Study of Health of Pomerania’ are available from the University Medicine Greifswald, Germany, upon request at https://transfer.ship‐med.uni‐greifswald.de/FAIRequest/ and with permission of the University Medicine Greifswald.
