# Supplementary material for: Socioeconomic Status, Alcohol Use, and Mortality: Results From a Prospective Cohort Study
Source: Alcohol Clin Exp Res (Hoboken). 2026 Sep 5;50(9):e70402. doi: 10.1111/acer.70402 (PMC13545546; doi:10.1111/acer.70402)
Supplement: Supplementary file 1 [file ACER-50-0-s002.pdf]

Supplementary Material

Captions for Figures

Figure 1

Graph of the scaled schoenfeld residuals for variable sex against transformed time in model 5a using income as SES proxy

Figure 2

Graph of the scaled schoenfeld residuals for variable age against transformed time in model 5a using income as SES proxy

Figure 3

Graph of the scaled schoenfeld residuals for variable marital status against transformed time in model 5a using income as SES proxy

Figure 4

Graph of the scaled schoenfeld residuals for variable sex against transformed time in model 5a using education as SES proxy

Figure 5

Graph of the scaled schoenfeld residuals for variable age against transformed time in model 5a using education as SES proxy

Figure 6

Plot illustrating risk of death according to net equivalent income in cox regression model 5a with penalized splines

Figure 7

Plots illustrating risk of death according to alcohol biomarkers in cox regression models 4a-4e with penalized splines

Figure 8

Plots illustrating risk of death according to alcohol biomarkers in cox regression models 5a-5e with penalized splines

Figures

Figure 1

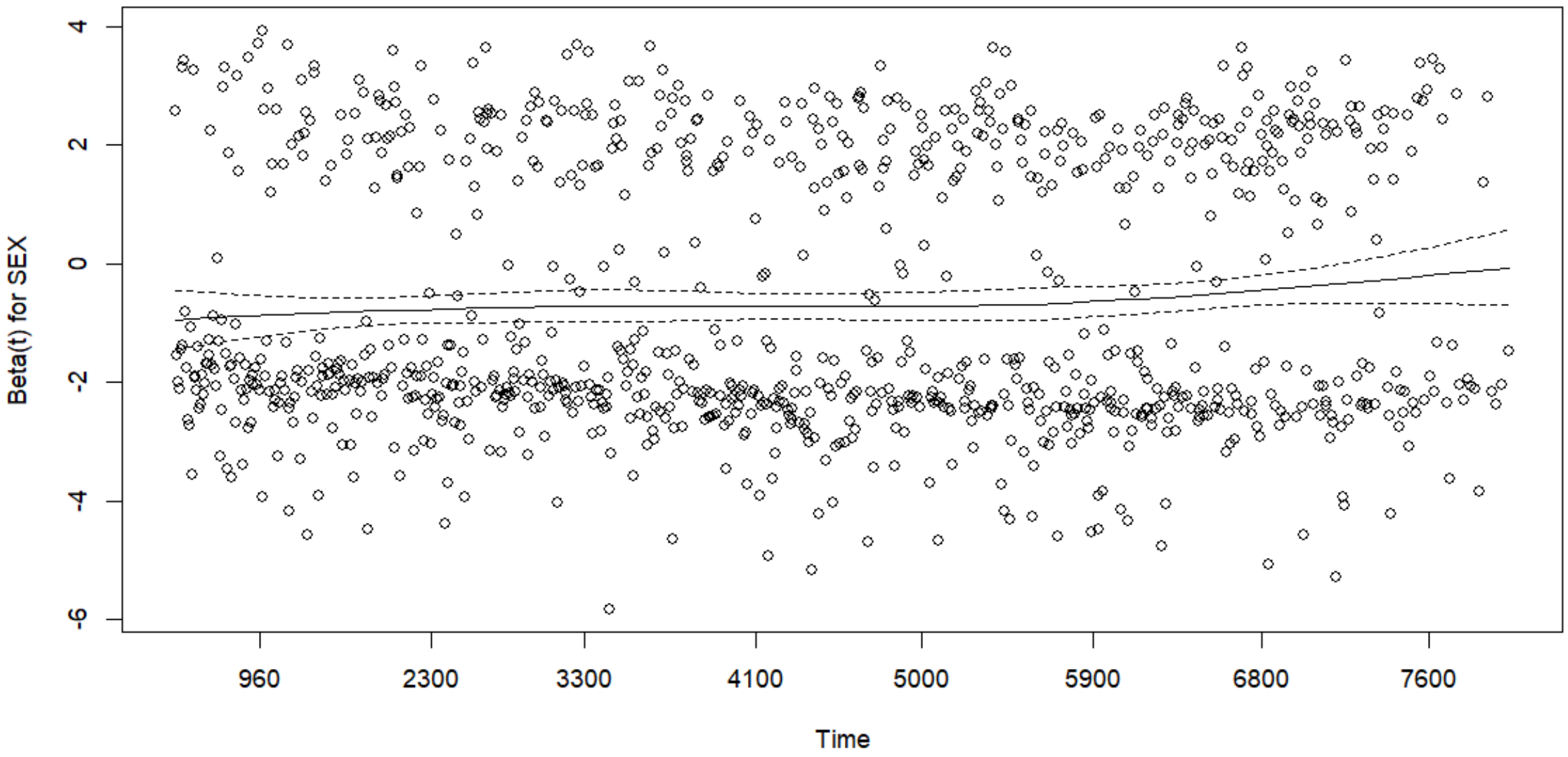

Figure 2

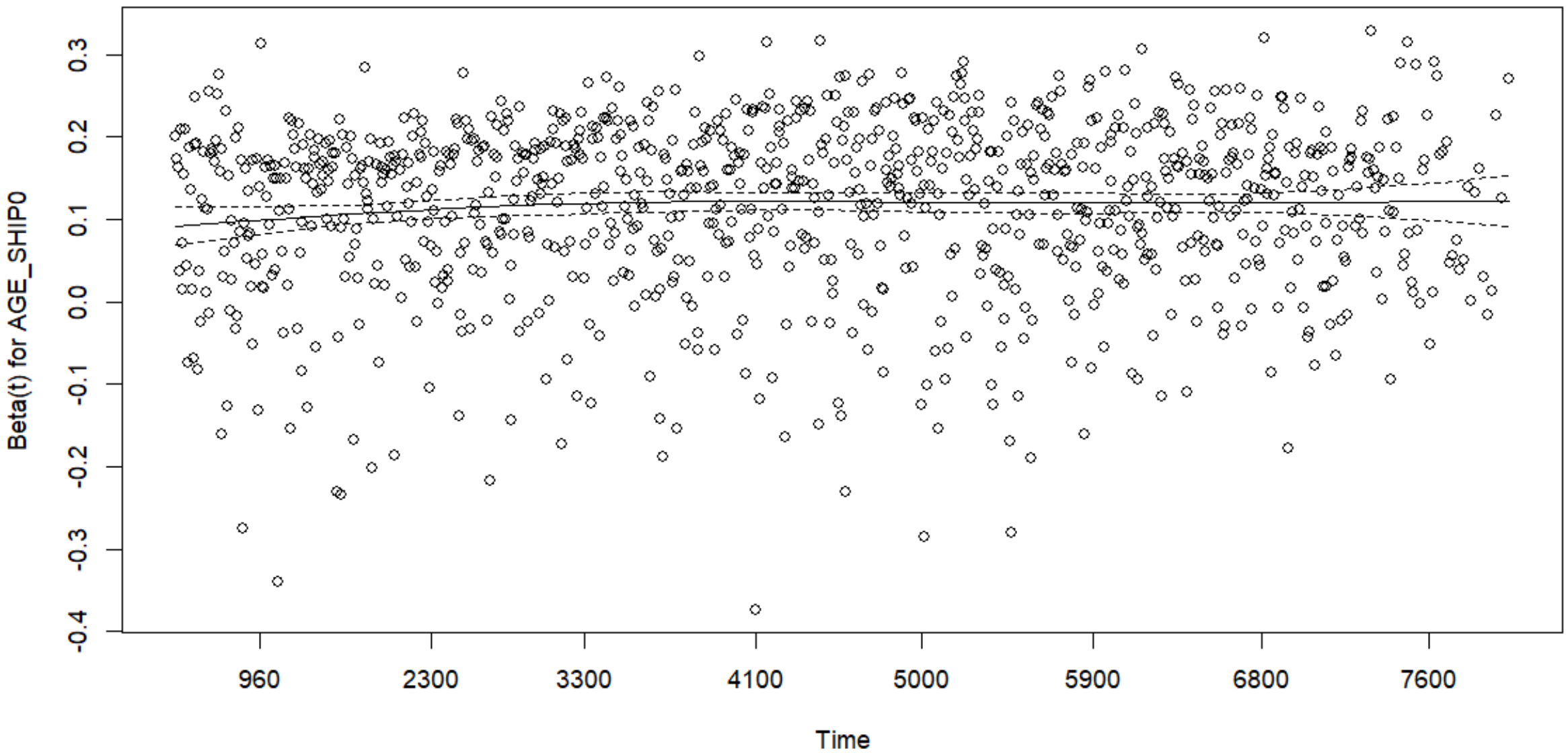

Figure 3

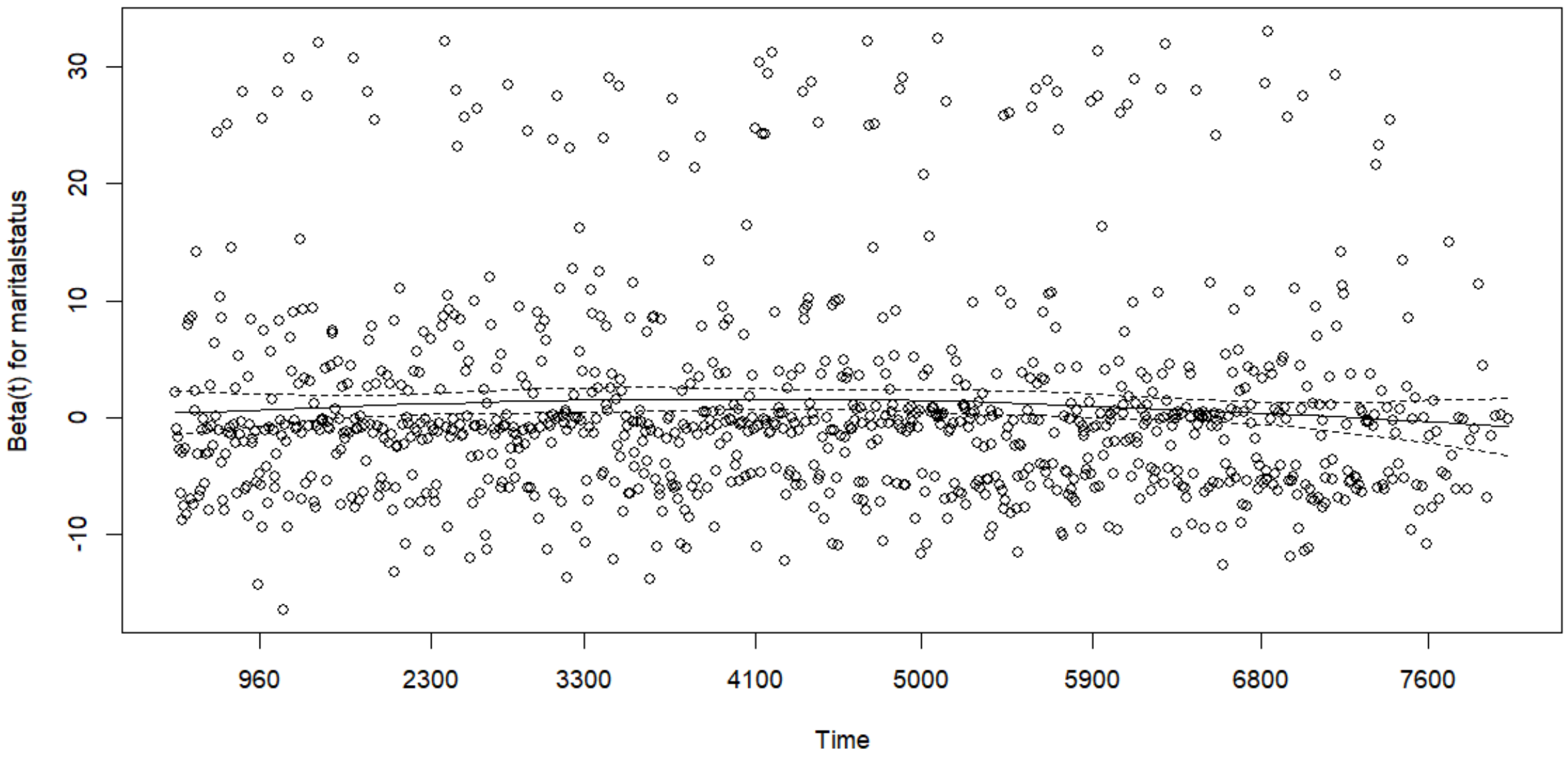

Figure 4

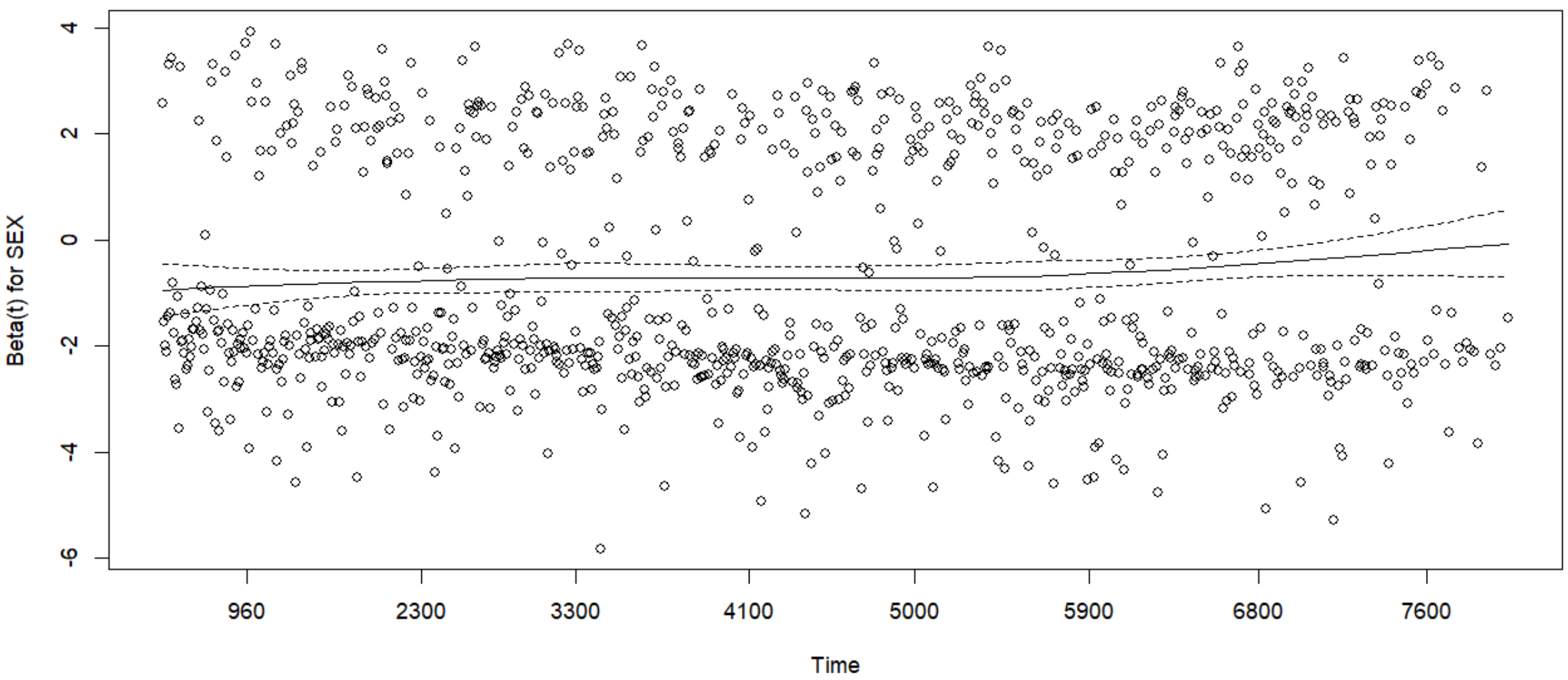

Figure 5

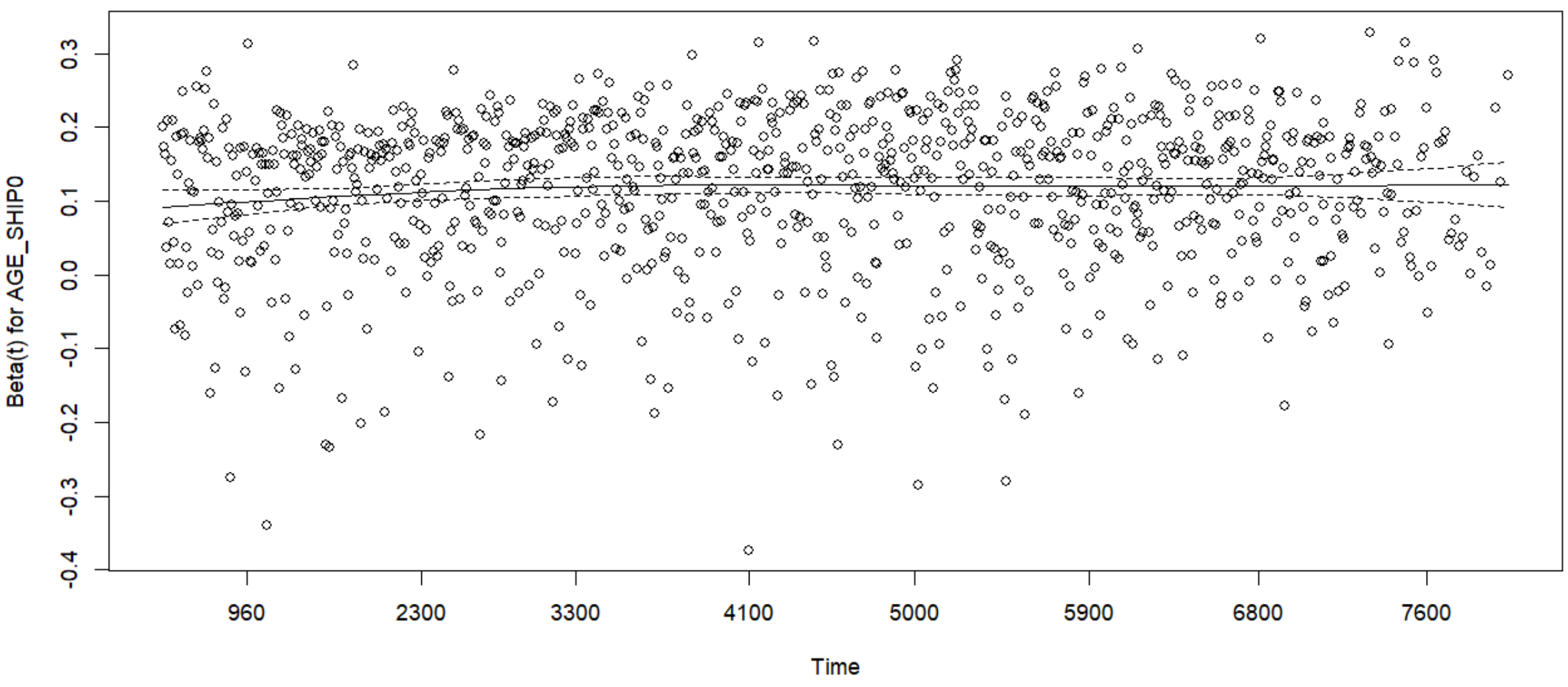
Beta(t) for AGE_SHIP0
0.3
0.2
0.1
0.0
-0.1
-0.2
-0.3
-0.4
960
2300
3300
4100
5000
5900
6800
7600
Time

37

Figure 6

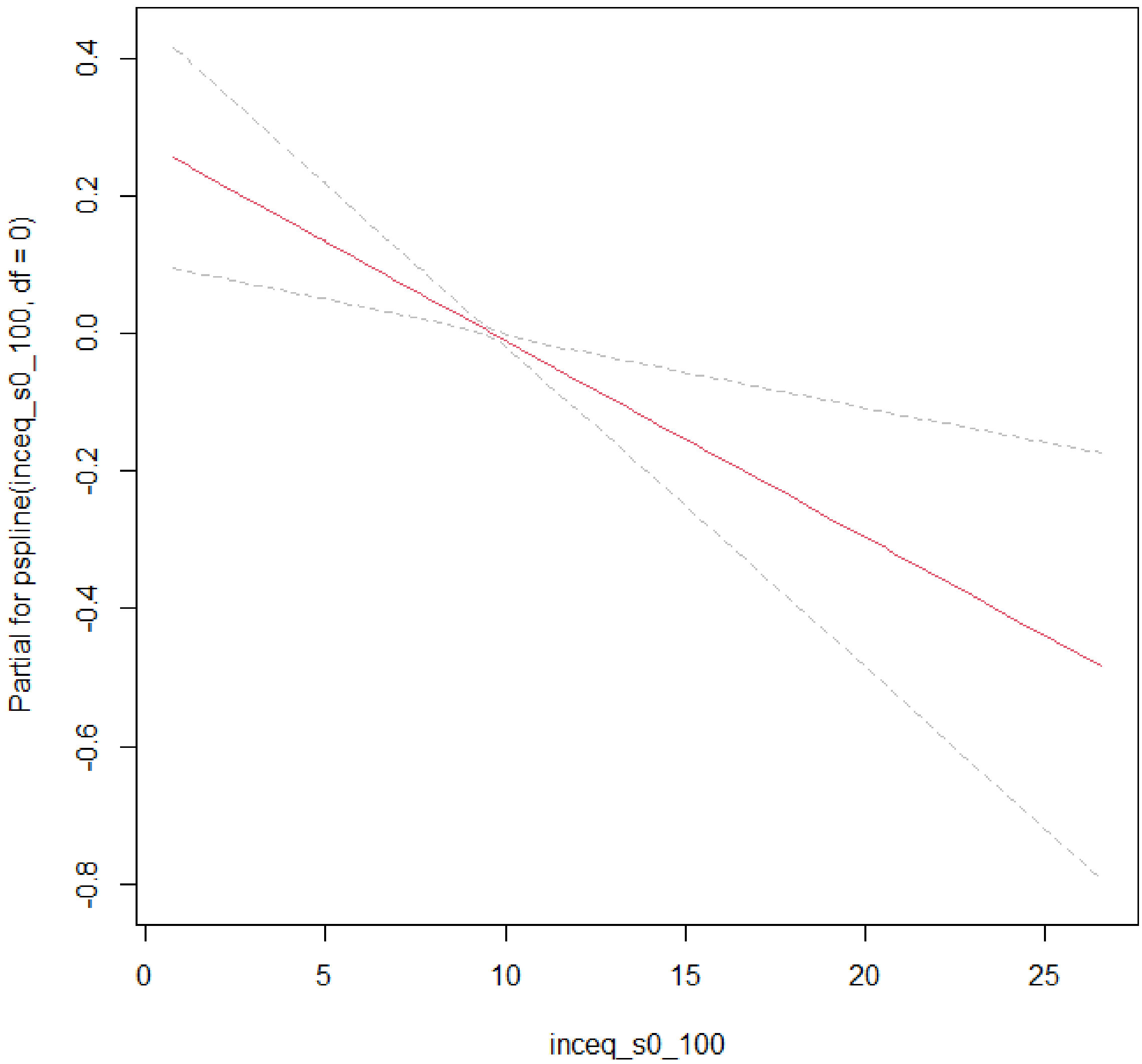
Partial for pspline(inceq_s0_100, df = 0)
0.4
0.2
0.0
-0.2
-0.4
-0.6
-0.8
0
5
10
15
20
25
inceq_s0_100

Figure 7

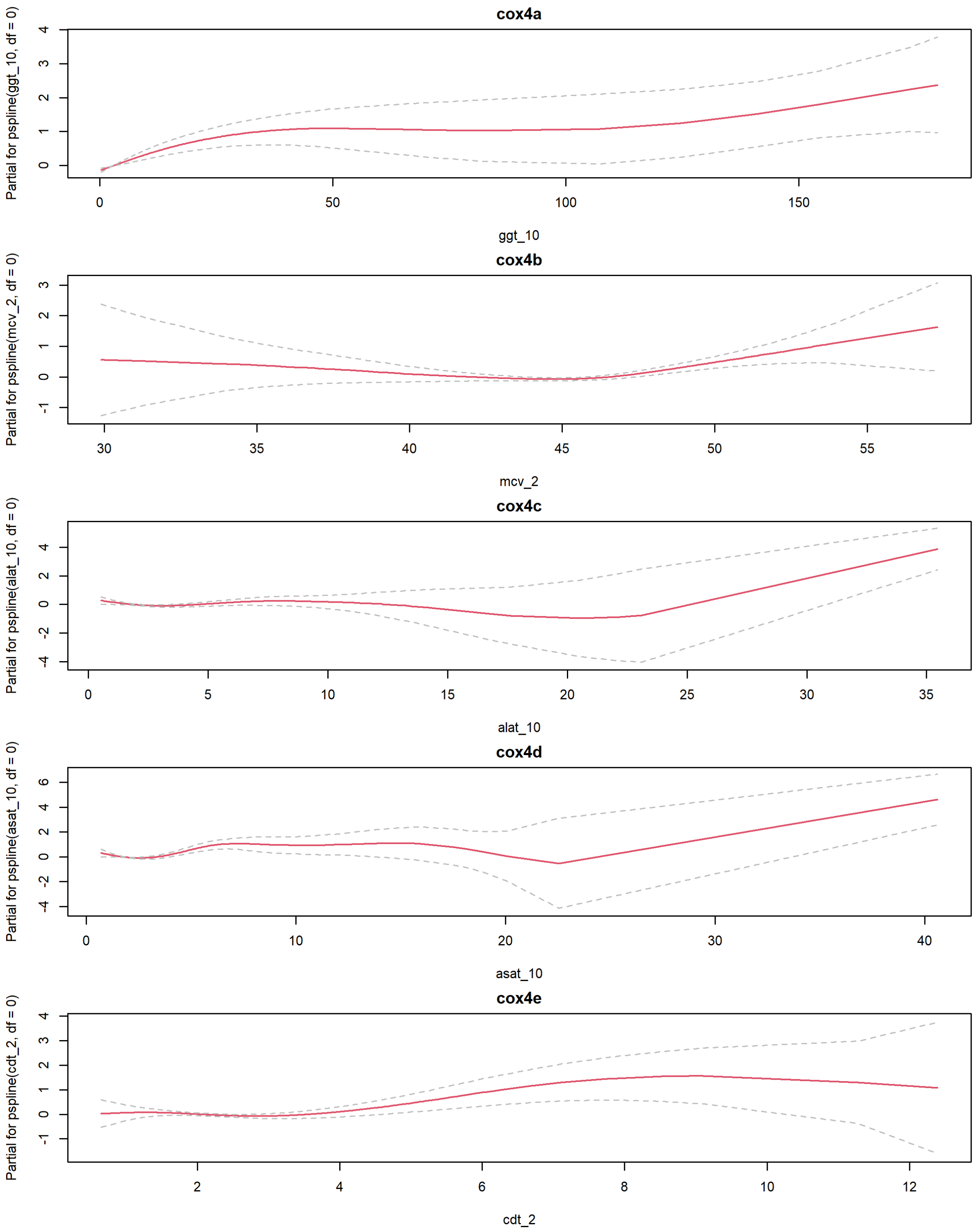

cox4a
Partial for pspline(ggt_10, df = 0)
0 1 2 3 4
0 50 100 150
ggt_10
cox4b
Partial for pspline(mcv_2, df = 0)
-1 0 1 2 3
30 35 40 45 50 55
mcv_2
cox4c
Partial for pspline(alat_10, df = 0)
-4 -2 0 2 4
0 5 10 15 20 25 30 35
alat_10
cox4d
Partial for pspline(asat_10, df = 0)
-4 -2 0 2 4 6
0 10 20 30 40
asat_10
cox4e
Partial for pspline(cdt_2, df = 0)
-1 0 1 2 3 4
2 4 6 8 10 12
cdt_2

Figure 8

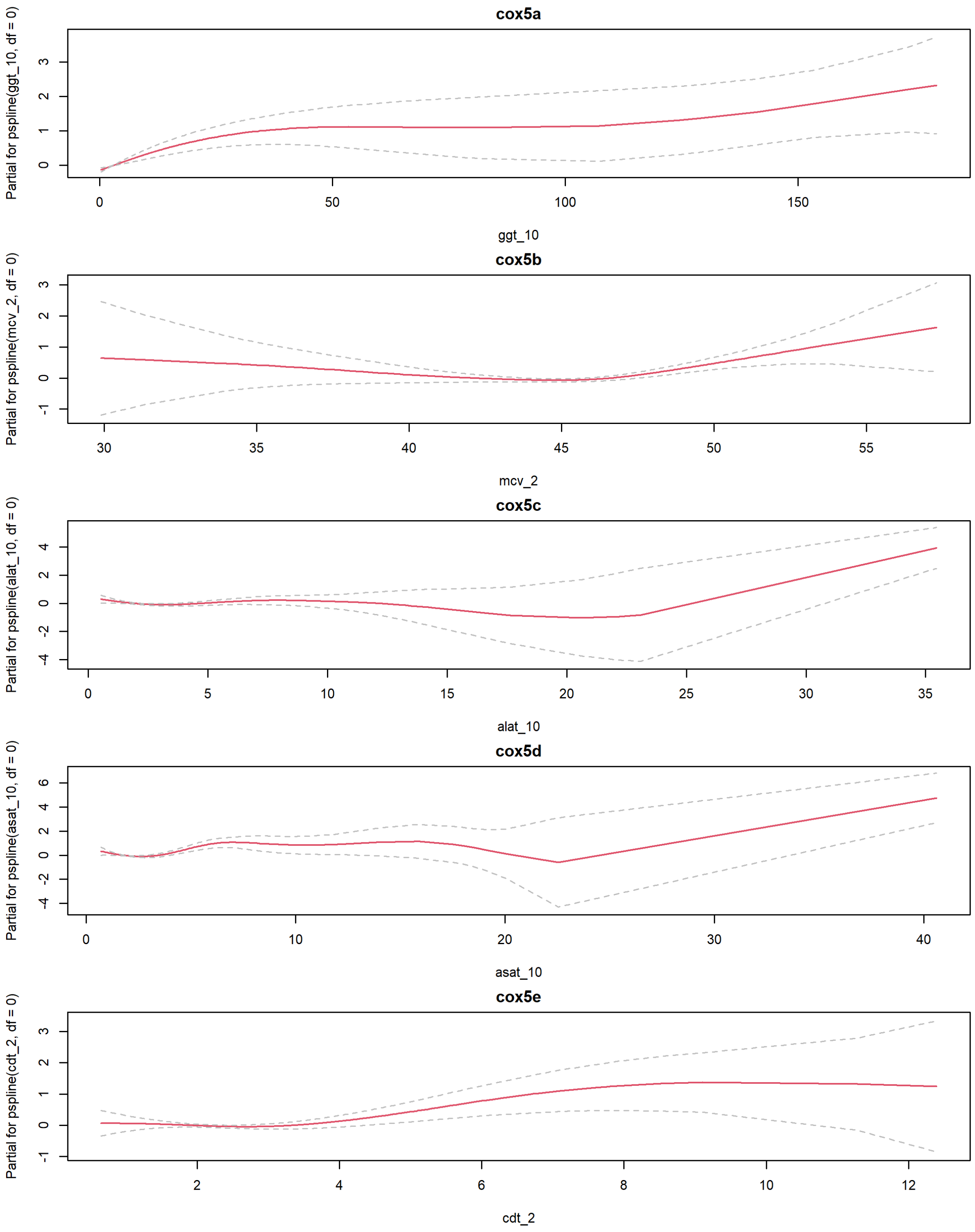
cox5a
Partial for pspline(ggt_10, df = 0)
ggt_10
cox5b
Partial for pspline(mcv_2, df = 0)
mcv_2
cox5c
Partial for pspline(alat_10, df = 0)
alat_10
cox5d
Partial for pspline(asat_10, df = 0)
asat_10
cox5e
Partial for pspline(cdt_2, df = 0)
cdt_2
