# Supplementary material for: Socioeconomic Status, Alcohol Use, and Mortality: Results From a Prospective Cohort Study
Source: Alcohol Clin Exp Res (Hoboken). 2026 Sep 5;50(9):e70402. doi: 10.1111/acer.70402 (PMC13545546; doi:10.1111/acer.70402)
Supplement: Supplementary file 2 [file ACER-50-0-s001.docx]

**Supplementary Material A**

**Tables**

**Table A:** *Test for violations of the proportional hazards assumption in models 5a using either income or education as SES proxy.*

**Table B:** *Frequency of missing data.*

**Table C:** *Descriptive statistics on 4307 participants (dataset including missings) and 3939 participants by education group.*

**Table D:** *Risk of death according to income (tercile) in cox regression models 4a-4e.*

**Table E:** *Risk of death according to income (tercile) in cox regression models 5b-5e.*

**Table F:** *Risk of death according to education in cox regression models 1, 2, 3 and 5a.*

**Table G:** *Risk of death according to education in cox regression models 4a-4e.*

**Table H:** *Risk of death according to education in cox regression models 5b-5e.*

**Table I:** *Predictive validity in cox regression models including income*.

**Table J:** *Risk of death according to alcohol biomarkers in cox regression models 4a-4e with penalized splines.*

**Table K:** *Risk of death according to alcohol biomarkers in cox regression models 5a-5e with penalized splines.*

**Table L:** *Risk of death according to net equivalent income in cox regression models 1, 2, 3 and 5a.*

**Table M:** *Risk of death according to net equivalent income in cox regression models 4a-4e.*

**Table N:** *Risk of death according to net equivalent income in cox regression models 5b-5e.*

**Table O:** *Comparison of models 5a with and without penalized splines.*

**Table P:** *Risk of death according to alcohol use in sensitivity analyses using cox regression models 3 with age and sex as time-varying variables and income (tercile) as SES proxy.*

**Table Q:** *Risk of death according to interactions of education and alcohol use in cox regression models and aalen additive models.*

**Table A**

*Test for violations of the proportional hazards assumption in models 5a using either income or education as SES proxy.*

|  | Model 5a using income | | | Model 5a using education | | |
| --- | --- | --- | --- | --- | --- | --- |
|  | *Χ*^2^ | Degrees of freedom | *p* | *Χ*^2^ | Degrees of freedom | *p* |
| Sex | 5.08 | 1 | 0.024 | 3.96 | 1 | 0.047 |
| Age | 5.33 | 1 | 0.021 | 4.62 | 1 | 0.032 |
| Income | 0.05 | 2 | 0.974 |  |  |  |
| Education |  |  |  | 0.19 | 2 | 0.909 |
| Marital Status | 8.45 | 3 | 0.038 | 6.41 | 3 | 0.093 |
| Smoking | 3.55 | 2 | 0.169 | 3.19 | 2 | 0.203 |
| BMI (kg/m^2^) | 0.28 | 1 | 0.596 | 0.03 | 1 | 0.865 |
| Alcohol Use | 1.74 | 4 | 0.783 | 1.67 | 4 | 0.797 |
| GGT | 0.29 | 1 | 0.588 | 0.31 | 1 | 0.578 |
| Global | 20.82 | 15 | 0.143 | 18.17 | 15 | 0.254 |

*Notes:* Model 5a is adjusted for age, sex, education, marital status, smoking, BMI, alcohol use and GGT. BMI = body mass index; GGT = gamma-glutamyltransferase.

**Table B**

*Frequency of missing data.*

| Variable | *N* (missing) | *N* (available) |
| --- | --- | --- |
| Death | 0 | 4307 |
| Sex | 0 | 4307 |
| Age | 0 | 4307 |
| Income | 251 | 4056 |
| Education | 30 | 4277 |
| SF-12 |  |  |
| *PCS* | 319 | 3988 |
| *MCS* | 319 | 3988 |
| BMI (kg/m^2^) | 10 | 4297 |
| Smoking | 19 | 4288 |
| Alcohol use | 268 | 4039 |
| Biomarker |  |  |
| *GGT (U/l)* | 51 | 4256 |
| *ALT (U/l)* | 30 | 4277 |
| *AST (U/l)* | 21 | 4286 |
| *MCV (fl)* | 17 | 4290 |
| *CDT (%)* | 16 | 4291 |

*Notes:* Using income as a SES proxy, 536 (12.4%) were excluded due to missing data in any of the exposure or confounder variables included in models 5a-e, resulting in a final study population of 3771 individuals. Using education as an SES proxy, 368 (8.5%) were excluded due to missing data resulting in a final study population of 3939 individuals.

**Table C**

*Descriptive statistics on 4307 participants (dataset including missings) and 3939 participants by education group.*

|  |  |  | Education | | |
| --- | --- | --- | --- | --- | --- |
|  | Dataset including missings | Full sample | < 10 years | 10 years | > years |
| Total participants (*N*) | 4307 | 3939 | 1532 | 1735 | 672 |
| Deaths | 27.7 | 26.6 | 48.6 | 11.4 | 15.6 |
| Sex (% female) | 50.9 | 51.0 | 48.6 | 54.2 | 48.4 |
| Age | 49.81 (16.39) | 49.43 (16.35) | 60.97 (12.92) | 41.35 (13.04) | 43.97 (15.70) |
| SF-12 |  |  |  |  |  |
| *PCS* | 48.04 (8.99) | 48.13 (8.91) | 44.68 (9.82) | 49.92 (7.69) | 51.05 (7.18) |
| *MCS* | 51.93 (8.31) | 51.93 (8.27) | 52.71 (8.59) | 51.82 (7.90) | 50.55 (8.33) |
| BMI (kg/m^2^) | 27.28 (4.76) | 27.23 (4.75) | 28.60 (4.73) | 26.55 (4.65) | 25.86 (4.26) |
| Smoking |  |  |  |  |  |
| *Never smoker (%)* | 35.8 | 35.9 | 38.9 | 30.8 | 42.3 |
| *Former smoker (%)* | 33.9 | 33.6 | 37.3 | 30.4 | 33.6 |
| *Current smoker (%)* | 30.3 | 30.5 | 23.8 | 38.8 | 24.1 |
| Alcohol use |  |  |  |  |  |
| *Lifetime abstainers (%)* | 2.6 | 2.6 | 5.0 | 1.2 | 0.7 |
| *Current abstainers (%)* | 5.7 | 5.6 | 10.0 | 3.1 | 1.9 |
| *Low intake (%)* | 61.7 | 61.7 | 63.3 | 61.8 | 57.9 |
| *Moderate intake (%)* | 21.1 | 21.1 | 15.1 | 23.2 | 29.3 |
| *High intake (%)* | 8.9 | 9.0 | 6.7 | 10.7 | 10.1 |
| Biomarker |  |  |  |  |  |
| *GGT (U/l)* | 35.25 (75.09) | 35.11 (77.02) | 37.70 (72.91) | 34.44 (83.72) | 30.92 (67.38) |
| *ALT (U/l)* | 28.41 (20.02) | 28.52 (20.33) | 28.40 (19.15) | 29.44 (22.65) | 26.43 (16.07) |
| *AST (U/l)* | 22.13 (13.45) | 22.18 (13.59) | 22.64 (12.57) | 22.22 (15.95) | 21.02 (7.97) |
| *MCV (fl)* | 90.16 (4.51) | 90.11 (4.48) | 90.45 (4.68) | 89.93 (4.34) | 89.79 (4.34) |
| *CDT (%)* | 4.83 (1.65) | 4.82 (1.64) | 4.75 (1.67) | 4.90 (1.71) | 4.76 (1.33) |

*Notes:* Numbers display absolute values (*N*), percentages (*%*) or mean (standard deviation in brackets). PCS = physical component summary; MCS = mental component summary; BMI = body mass index; GGT = gamma-glutamyltransferase; ALT = alanine transaminase; AST = aspartate transaminase; MCV = mean corpuscular volume; CDT = carbohydrate-deficient transferrin.

**Table D**

*Risk of death according to income (tercile) in cox regression models 4a-4e.*

|  | Model 4a |  | Model 4b |  | Model 4c |  | Model 4d |  | Model 4e |  |
| --- | --- | --- | --- | --- | --- | --- | --- | --- | --- | --- |
|  | Harrel’s C: 0.863 |  | Harrel’s C: 0.862 |  | Harrel’s C: 0.861 |  | Harrel’s C: 0.862 |  | Harrel’s C: 0.861 |  |
|  | HR | 95% CI | HR | 95% CI | HR | 95% CI | HR | 95% CI | HR | 95% CI |
| Age (t = 1) | 1.11 | 1.10, 1.12 | 1.11 | 1.10,  1.12 | 1.11 | 1.10,  1.12 | 1.11 | 1.10,  1.12 | 1.11 | 1.10,  1.12 |
| Age (t = 2) | 1.01 | 1.00, 1.03 | 1.02 | 1.00, 1.03 | 1.02 | 1.00, 1.03 | 1.01 | 1.00, 1.03 | 1.02 | 1.00, 1.03 |
| Age (t = 3) | 1.02 | 1.00, 1.04 | 1.02 | 1.00, 1.04 | 1.02 | 1.00, 1.04 | 1.02 | 1.00, 1.04 | 1.02 | 1.00, 1.04 |
| Sex |  |  |  |  |  |  |  |  |  |  |
| *Male* | reference |  | reference |  | reference |  | reference |  | reference |  |
| *Female (t = 1)* | 0.44 | 0.33, 0.57 | 0.43 | 0.33,  0.56 | 0.42 | 0.32,  0.55 | 0.43 | 0.33,  0.56 | 0.41 | 0.32,  0.54 |
| *Female (t = 2)* | 1.13 | 0.82, 1.55 | 1.13 | 0.82, 1.55 | 1.14 | 0.83, 1.57 | 1.14 | 0.83, 1.56 | 1.14 | 0.83, 1.56 |
| *Female (t = 3)* | 1.54 | 1.07, 2.21 | 1.54 | 1.07, 2.20 | 1.58 | 1.10, 2.26 | 1.57 | 1.09, 2.25 | 1.57 | 1.09, 2.25 |
| Income (tercile) |  |  |  |  |  |  |  |  |  |  |
| *1 (lowest)* | 1.37 | 1.16, 1.63 | 1.36 | 1.15,  1.61 | 1.36 | 1.15,  1.61 | 1.35 | 1.14,  1.61 | 1.36 | 1.14,  1.61 |
| *2* | 1.05 | 0.90, 1.23 | 1.03 | 0.88,  1.20 | 1.03 | 0.89,  1.20 | 1.03 | 0.89,  1.20 | 1.03 | 0.89,  1.20 |
| *3 (highest)* | reference |  | reference |  | reference |  | reference |  | reference |  |
| Marital status |  |  |  |  |  |  |  |  |  |  |
| *Married* | reference |  | reference |  | reference |  | reference |  | reference |  |
| *Divorced* | 1.23 | 0.95,  1.59 | 1.25 | 0.97,  1.62 | 1.23 | 0.95,  1.59 | 1.22 | 0.95,  1.58 | 1.23 | 0.95,  1.59 |
| *Single* | 1.65 | 1.27,  2.14 | 1.71 | 1.32,  2.21 | 1.70 | 1.31,  2.20 | 1.70 | 1.31,  2.20 | 1.71 | 1.32,  2.21 |
| *Widowed* | 1.17 | 0.98,  1.41 | 1.19 | 0.99,  1.43 | 1.20 | 1.00,  1.44 | 1.19 | 0.99,  1.43 | 1.20 | 1.00,  1.44 |
| Smoking |  |  |  |  |  |  |  |  |  |  |
| *Never smoker* | reference |  | reference |  | reference |  | reference |  | reference |  |
| *Former smoker* | 1.07 | 0.92,  1.26 | 1.06 | 0.91,  1.25 | 1.07 | 0.91,  1.25 | 1.07 | 0.91,  1.25 | 1.07 | 0.91,  1.25 |
| *Current smoker* | 1.90 | 1.57,  2.29 | 1.85 | 1.53,  2.24 | 1.94 | 1.61,  2.35 | 1.95 | 1.61,  2.36 | 1.93 | 1.59,  2.33 |
| BMI (kg/m^2^) | 1.03 | 1.01,  1.04 | 1.03 | 1.02,  1.05 | 1.03 | 1.01,  1.04 | 1.03 | 1.02,  1.05 | 1.03 | 1.02,  1.05 |
| Biomarker |  |  |  |  |  |  |  |  |  |  |
| *GGT* | 1.01 | 1.01, 1.02 |  |  |  |  |  |  |  |  |
| *MCV* |  |  | 1.04 | 1.01, 1.08 |  |  |  |  |  |  |
| *ALT* |  |  |  |  | 1.03 | 0.99, 1.07 |  |  |  |  |
| *AST* |  |  |  |  |  |  | 1.09 | 1.05, 1.13 |  |  |
| *CDT* |  |  |  |  |  |  |  |  | 1.07 | 0.98, 1.16 |

*Notes:* Models with income include 3771 participants. Model 4a-4e are adjusted for age, sex, income, marital status, smoking, BMI, and for one of the biomarkers in each case. Values of GGT, ALT and AST were divided by 10 and values of MCV and CDT were divided by two, so that the estimates now represent changes per 10-unit or 2-unit increase in the biomarker level. HR = hazard ratio; CI = confidence interval; BMI = body mass index; GGT = gamma-glutamyltransferase; ALT = alanine transaminase; AST = aspartate transaminase; MCV = mean corpuscular volume; CDT = carbohydrate-deficient transferrin.

**Table E**

*Risk of death according to income (tercile) in cox regression models 5b-5e.*

|  | Model 5b | | Model 5c | | Model 5d | | Model 5e | |
| --- | --- | --- | --- | --- | --- | --- | --- | --- |
|  | Harrel’s C: 0.863 | | Harrel’s C: 0.863 | | Harrel’s C: 0.864 | | Harrel’s C: 0.863 | |
|  | HR | 95% CI | HR | 95% CI | HR | 95% CI | HR | 95% CI |
| Age (t = 1) | 1.11 | 1.09,  1.12 | 1.11 | 1.10,  1.12 | 1.11 | 1.10,  1.12 | 1.11 | 1.10,  1.12 |
| Age (t = 2) | 1.02 | 1.00,  1.03 | 1.02 | 1.00,  1.03 | 1.01 | 1.00,  1.03 | 1.02 | 1.00,  1.03 |
| Age (t = 3) | 1.02 | 1.00,  1.04 | 1.02 | 1.00,  1.04 | 1.02 | 1.00,  1.04 | 1.02 | 1.00,  1.04 |
| Sex |  |  |  |  |  |  |  |  |
| *Male* | reference |  | reference |  | reference |  | reference |  |
| *Female (t = 1)* | 0.43 | 0.32,  0.56 | 0.42 | 0.32,  0.55 | 0.42 | 0.32,  0.56 | 0.41 | 0.32,  0.54 |
| *Female (t = 2)* | 1.12 | 0.82,  1.54 | 1.14 | 0.83,  1.56 | 1.14 | 0.83,  1.56 | 1.14 | 0.83,  1.56 |
| *Female (t = 3)* | 1.52 | 1.06,  2.19 | 1.57 | 1.09,  2.25 | 1.56 | 1.09,  2.24 | 1.56 | 1.09,  2.23 |
| Income (tercile) |  |  |  |  |  |  |  |  |
| *1 (lowest)* | 1.31 | 1.10,  1.56 | 1.31 | 1.10,  1.56 | 1.30 | 1.09,  1.54 | 1.30 | 1.10,  1.55 |
| *2* | 1.01 | 0.87,  1.18 | 1.01 | 0.87,  1.18 | 1.01 | 0.87,  1.18 | 1.01 | 0.87,  1.18 |
| *3 (highest)* | reference |  | reference |  | reference |  | reference |  |
| Marital status |  |  |  |  |  |  |  |  |
| *Married* | reference |  | reference |  | reference |  | reference |  |
| *Divorced* | 1.26 | 0.97,  1.62 | 1.24 | 0.96,  1.60 | 1.23 | 0.96,  1.59 | 1.24 | 0.96,  1.60 |
| *Single* | 1.66 | 1.28,  2.15 | 1.65 | 1.27,  2.14 | 1.65 | 1.28,  2.14 | 1.66 | 1.28,  2.15 |
| *Widowed* | 1.17 | 0.97,  1.40 | 1.17 | 0.97,  1.40 | 1.16 | 0.97,  1.39 | 1.17 | 0.98,  1.41 |
| Smoking |  |  |  |  |  |  |  |  |
| *Never smoker* | reference |  | reference |  | reference |  | reference |  |
| *Former smoker* | 1.08 | 0.92,  1.27 | 1.08 | 0.92,  1.27 | 1.08 | 0.92,  1.27 | 1.08 | 0.92,  1.27 |
| *Current smoker* | 1.86 | 1.53,  2.25 | 1.94 | 1.60,  2.35 | 1.95 | 1.61,  2.37 | 1.93 | 1.59,  2.33 |
| BMI (kg/m^2^) | 1.03 | 1.02,  1.04 | 1.03 | 1.01,  1.04 | 1.03 | 1.01,  1.04 | 1.03 | 1.02 |
| Alcohol use |  |  |  |  |  |  |  |  |
| *Lifetime abstainers* | 1.15 | 0.81,  1.64 | 1.13 | 0.79,  1.60 | 1.13 | 0.79,  1.61 | 1.16 | 0.81,  1.65 |
| *Current abstainers* | 1.53 | 1.24,  1.88 | 1.55 | 1.26,  1.91 | 1.56 | 1.27,  1.92 | 1.59 | 1.29,  1.96 |
| *Low intake* | reference |  | reference |  | reference |  | reference |  |
| *Moderate intake* | 0.84 | 0.70,  1.01 | 0.86 | 0.71,  1.03 | 0.85 | 0.70,  1.02 | 0.85 | 0.71,  1.02 |
| *High intake* | 1.25 | 0.98,  1.59 | 1.28 | 1.01,  1.63 | 1.22 | 0.96,  1.56 | 1.26 | 0.99,  1.61 |
| Biomarker |  |  |  |  |  |  |  |  |
| *MCV* | 1.04 | 1.01,  1.07 |  |  |  |  |  |  |
| *ALT* |  |  | 1.02 | 0.99,  1.06 |  |  |  |  |
| *AST* |  |  |  |  | 1.09 | 1.05,  1.13 |  |  |
| *CDT* |  |  |  |  |  |  | 1.08 | 1.00,  1.18 |

*Notes:* Models with income include 3771 participants. Model 5b-5e are adjusted for age, sex, income, marital status, smoking, BMI, alcohol use and for one of the biomarkers in each case. Values of ALT and AST were divided by 10 and values of MCV and CDT were divided by two, so that the estimates now represent changes per 10-unit or 2-unit increase in the biomarker level. HR = hazard ratio; CI = confidence interval; BMI = body mass index; MCV = mean corpuscular volume; ALT = alanine transaminase; AST = aspartate transaminase; CDT = carbohydrate-deficient transferrin.

**Table F**

*Risk of death according to education in cox regression models 1, 2, 3 and 5a.*

|  | Model 1 |  | Model 2 |  | Model 3 |  | Model 5a |  |
| --- | --- | --- | --- | --- | --- | --- | --- | --- |
|  | HR | 95% CI | HR | 95% CI | HR | 95% CI | HR | 95% CI |
| Age (t = 1) | 1.10 | 1.08, 1.11 | 1.11 | 1.09, 1.12 | 1.11 | 1.09, 1.12 | 1.11 | 1.09,  1.12 |
| Age (t = 2) | 1.01 | 1.00,  1.03 | 1.01 | 1.00,  1.03 | 1.01 | 1.00,  1.03 | 1.01 | 1.00,  1.03 |
| Age (t = 3) | 1.02 | 1.00,  1.03 | 1.02 | 1.00,  1.03 | 1.02 | 1.00,  1.04 | 1.02 | 1.00,  1.03 |
| Sex |  |  |  |  |  |  |  |  |
| *Male* | reference |  | reference |  | reference |  | reference |  |
| *Female (t = 1)* | 0.46 | 0.36, 0.59 | 0.43 | 0.33,  0.55 | 0.42 | 0.33, 0.55 | 0.44 | 0.34,  0.58 |
| *Female (t = 2)* | 1.08 | 0.80,  1.46 | 1.07 | 0.79,  1.44 | 1.06 | 0.79,  1.44 | 1.05 | 0.78,  1.42 |
| *Female (t = 3)* | 1.45 | 1.02,  2.05 | 1.44 | 1.02,  2.04 | 1.43 | 1.01,  2.02 | 1.40 | 0.99,  1.99 |
| Education |  |  |  |  |  |  |  |  |
| *< 10 years* | 1.62 | 1.32, 2.00 | 1.49 | 1.21, 1.84 | 1.46 | 1.18, 1.80 | 1.45 | 1.18,  1.79 |
| *10 years* | 1.26 | 0.99, 1.59 | 1.20 | 0.94, 1.52 | 1.19 | 0.94, 1.52 | 1.17 | 0.92,  1.49 |
| *> 10 years* | reference |  | reference |  | reference |  | reference |  |
| Marital status |  |  |  |  |  |  |  |  |
| *Married* |  |  | reference |  | reference |  | reference |  |
| *Divorced* |  |  | 1.35 | 1.06, 1.73 | 1.35 | 1.06, 1.73 | 1.34 | 1.05,  1.72 |
| *Single* |  |  | 1.89 | 1.48, 2.42 | 1.83 | 1.43, 2.35 | 1.78 | 1.39,  2.28 |
| *Widowed* |  |  | 1.23 | 1.04, 1.48 | 1.22 | 1.03, 1.46 | 1.19 | 1.00,  1.42 |
| Smoking |  |  |  |  |  |  |  |  |
| *Never smoker* |  |  | reference |  | reference |  | reference |  |
| *Former smoker* |  |  | 1.03 | 0.88, 1.20 | 1.04 | 0.89, 1.22 | 1.05 | 0.90,  1.23 |
| *Current smoker* |  |  | 2.02 | 1.68, 2.42 | 2.01 | 1.68, 2.42 | 1.99 | 1.66,  2.39 |
| BMI (kg/m^2^) |  |  | 1.03 | 1.02, 1.05 | 1.03 | 1.02, 1.04 | 1.03 | 1.01,  1.04 |
| Alcohol use |  |  |  |  |  |  |  |  |
| *Lifetime abstainers* |  |  |  |  | 1.07 | 0.75, 1.51 | 1.07 | 0.76,  1.52 |
| *Current abstainers* |  |  |  |  | 1.46 | 1.19, 1.77 | 1.47 | 1.21,  1.79 |
| *Low intake* |  |  |  |  | reference |  | reference |  |
| *Moderate intake* |  |  |  |  | 0.84 | 0.70, 1.01 | 0.83 | 0.69,  1.00 |
| *High intake* |  |  |  |  | 1.31 | 1.04, 1.66 | 1.21 | 0.96, 1.54 |
| GGT |  |  |  |  |  |  | 1.01 | 1.01,  1.02 |

*Notes:* Models with education include 3939 participants. Model 1 is adjusted for age, sex, and education; model 2 is model 1 + adjustment for marital status, smoking and BMI; model 3 is model 2 + adjustment for alcohol use; model 5a is model 3 + adjustment for GGT. Values of GGT were divided by 10, so that the estimates now represent changes per 10-unit increase. HR = hazard ratio; CI = confidence interval; BMI = body mass index; GGT = gamma-glutamyltransferase.

**Table G**

*Risk of death according to education in cox regression models 4a-4e.*

|  | Model 4a |  | Model 4b |  | Model 4c |  | Model 4d |  | Model 4e |  |
| --- | --- | --- | --- | --- | --- | --- | --- | --- | --- | --- |
|  | HR | 95% CI | HR | 95% CI | HR | 95% CI | HR | 95% CI | HR | 95% CI |
| Age (t = 1) | 1.11 | 1.10,  1.12 | 1.11 | 1.09,  1.12 | 1.11 | 1.09,  1.12 | 1.11 | 1.10,  1.12 | 1.11 | 1.09,  1.12 |
| Age (t = 2) | 1.01 | 1.00,  1.03 | 1.01 | 1.00,  1.03 | 1.01 | 1.00,  1.03 | 1.01 | 1.00,  1.03 | 1.01 | 1.00,  1.03 |
| Age (t = 3) | 1.02 | 1.00,  1.03 | 1.02 | 1.00,  1.03 | 1.02 | 1.00,  1.03 | 1.02 | 1.00,  1.03 | 1.02 | 1.00,  1.03 |
| Sex |  |  |  |  |  |  |  |  |  |  |
| *Male* |  |  |  |  |  |  |  |  |  |  |
| *Female (t = 1)* | 0.45 | 0.35,  0.58 | 0.44 | 0.34,  0.57 | 0.43 | 0.33,  0.56 | 0.44 | 0.34,  0.57 | 0.43 | 0.33,  0.55 |
| *Female (t = 2)* | 1.06 | 0.78,  1.43 | 1.06 | 0.78,  1.43 | 1.07 | 0.79,  1.44 | 1.07 | 0.79,  1.44 | 1.07 | 0.79,  1.44 |
| *Female (t = 3)* | 1.42 | 1.00,  2.01 | 1.42 | 1.00,  2.01 | 1.45 | 1.02,  2.05 | 1.44 | 1.02,  2.05 | 1.44 | 1.02,  2.05 |
| Education |  |  |  |  |  |  |  |  |  |  |
| *< 10 years* | 1.50 | 1.21,  1.84 | 1.49 | 1.21,  1.84 | 1.49 | 1.21,  1.84 | 1.48 | 1.20,  1.83 | 1.50 | 1.21,  1.84 |
| *10 years* | 1.18 | 0.93,  1.50 | 1.21 | 0.95,  1.53 | 1.19 | 0.94,  1.52 | 1.18 | 0.93,  1.50 | 1.20 | 0.94,  1.52 |
| *> 10 years* | reference |  | reference |  | reference |  | reference |  | reference |  |
| Marital status |  |  |  |  |  |  |  |  |  |  |
| *Married* | reference |  | reference |  | reference |  | reference |  |  |  |
| *Divorced* | 1.35 | 1.05,  1.73 | 1.37 | 1.07,  1.75 | 1.35 | 1.06,  1.73 | 1.34 | 1.05,  1.72 | 1.35 | 1.06,  1.73 |
| *Single* | 1.84 | 1.43,  2.35 | 1.90 | 1.48,  2.43 | 1.89 | 1.47,  2.41 | 1.88 | 1.47,  2.40 | 1.89 | 1.48,  2.42 |
| *Widowed* | 1.21 | 1.01,  1.44 | 1.23 | 1.03,  1.46 | 1.23 | 1.04,  1.47 | 1.22 | 1.03,  1.45 | 1.24 | 1.04,  1.47 |
| Smoking |  |  |  |  |  |  |  |  |  |  |
| *Never smoker* | reference |  | reference |  | reference |  | reference |  |  |  |
| *Former smoker* | 1.04 | 0.89,  1.21 | 1.03 | 0.88,  1.20 | 1.03 | 0.88,  1.20 | 1.03 | 0.88,  1.20 | 1.04 | 0.89,  1.21 |
| *Current smoker* | 1.99 | 1.65,  2.39 | 1.94 | 1.62,  2.34 | 2.03 | 1.69,  2.43 | 2.04 | 1.70,  2.45 | 2.01 | 1.68,  2.42 |
| BMI (kg/m^2^) | 1.03 | 1.01,  1.04 | 1.03 | 1.02,  1.05 | 1.03 | 1.01,  1.04 | 1.03 | 1.01,  1.04 | 1.03 | 1.02,  1.05 |
| *GGT* | 1.01 | 1.01,  1.02 |  |  |  |  |  |  |  |  |
| *MCV* |  |  | 1.04 | 1.01,  1.07 |  |  |  |  |  |  |
| *ALT* |  |  |  |  | 1.02 | 0.98,  1.06 |  |  |  |  |
| *AST* |  |  |  |  |  |  | 1.09 | 1.05,  1.13 |  |  |
| *CDT* |  |  |  |  |  |  |  |  | 1.07 | 0.99,  1.16 |

*Notes:* Models with education include 3939 participants. Model 4a-4e are adjusted for age, sex, education, marital status, smoking, BMI, and for one of the biomarkers in each case. Values of GGT, ALT and AST were divided by 10 and values of MCV and CDT were divided by two, so that the estimates now represent changes per 10-unit or 2-unit increase in the biomarker level. HR = hazard ratio; CI = confidence interval; BMI = body mass index; GGT = gamma-glutamyltransferase; ALT = alanine transaminase; AST = aspartate transaminase; MCV = mean corpuscular volume; CDT = carbohydrate-deficient transferrin.

**Table H**

*Risk of death according to education in cox regression models 5b-5e.*

|  | Model 5b | | Model 5c | | Model 5d | | Model 5e | |
| --- | --- | --- | --- | --- | --- | --- | --- | --- |
|  | HR | 95% CI | HR | 95% CI | HR | 95% CI | HR | 95% CI |
| Age (t = 1) | 1.10 | 1.09,  1.12 | 1.11 | 1.09,  1.12 | 1.11 | 1.09,  1.12 | 1.11 | 1.09,  1.12 |
| Age (t = 2) | 1.01 | 1.00,  1.03 | 1.01 | 1.00,  1.03 | 1.01 | 1.00,  1.03 | 1.01 | 1.00,  1.03 |
| Age (t = 3) | 1.02 | 1.00,  1.04 | 1.02 | 1.00,  1.04 | 1.02 | 1.00,  1.03 | 1.02 | 1.00,  1.04 |
| Sex |  |  |  |  |  |  |  |  |
| *Male* | reference |  | reference |  | reference |  | reference |  |
| *Female (t = 1)* | 0.44 | 0.34,  0.56 | 0.43 | 0.33,  0.55 | 0.43 | 0.33,  0.56 | 0.42 | 0.33,  0.55 |
| *Female (t = 2)* | 1.01 | 1.00,  1.03 | 1.06 | 0.79,  1.44 | 1.06 | 0.79,  1.44 | 1.06 | 0.79,  1.44 |
| *Female (t = 3)* | 1.02 | 1.00,  1.04 | 1.43 | 1.01,  2.03 | 1.43 | 1.01,  2.03 | 1.43 | 1.01,  2.02 |
| Education |  |  |  |  |  |  |  |  |
| *< 10 years* | 1.46 | 1.18,  1.80 | 1.46 | 1.18,  1.80 | 1.44 | 1.17,  1.78 | 1.46 | 1.18,  1.80 |
| *10 years* | 1.20 | 0.94,  1.53 | 1.19 | 0.94,  1.51 | 1.17 | 0.92,  1.49 | 1.20 | 0.94,  1.52 |
| *> 10 years* | reference |  | reference |  | reference |  | reference |  |
| Marital status |  |  |  |  |  |  |  |  |
| *Married* | reference |  | reference |  | reference |  | reference |  |
| *Divorced* | 1.36 | 1.06,  1.74 | 1.35 | 1.05,  1.72 | 1.34 | 1.05,  1.71 | 1.35 | 1.05,  1.73 |
| *Single* | 1.83 | 1.43,  2.35 | 1.83 | 1.43,  2.34 | 1.82 | 1.43,  2.33 | 1.83 | 1.43,  2.34 |
| *Widowed* | 1.21 | 1.02,  1.44 | 1.22 | 1.02,  1.45 | 1.20 | 1.01,  1.43 | 1.22 | 1.02,  1.45 |
| Smoking |  |  |  |  |  |  |  |  |
| *Never smoker* | reference |  | reference |  | reference |  | reference |  |
| *Former smoker* | 1.04 | 0.89,  1.22 | 1.04 | 0.89,  1.22 | 1.04 | 0.89,  1.22 | 1.05 | 0.90,  1.23 |
| *Current smoker* | 1.95 | 1.62,  2.35 | 2.02 | 1.68,  2.43 | 2.04 | 1.70,  2.46 | 2.01 | 1.68,  2.42 |
| BMI (kg/m^2^) | 1.03 | 1.02,  1.04 | 1.03 | 1.01,  1.04 | 1.03 | 1.01,  1.04 | 1.03 | 1.02,  1.05 |
| Alcohol use |  |  |  |  |  |  |  |  |
| *Lifetime abstainers* | 1.09 | 0.77,  1.55 | 1.06 | 0.75,  1.51 | 1.06 | 0.75,  1.51 | 1.10 | 0.77,  1.55 |
| *Current abstainers* | 1.44 | 1.18,  1.75 | 1.45 | 1.19,  1.77 | 1.46 | 1.20,  1.78 | 1.49 | 1.22,  1.81 |
| *Low intake* | reference |  | reference |  | reference |  | reference |  |
| *Moderate intake* | 0.82 | 0.69,  0.99 | 0.84 | 0.70,  1.01 | 0.83 | 0.69,  0.99 | 0.83 | 0.69,  1.00 |
| *High intake* | 1.26 | 0.99,  1.60 | 1.30 | 1.02,  1.64 | 1.22 | 0.96,  1.55 | 1.26 | 1.00,  1.61 |
| Biomarker |  |  |  |  |  |  |  |  |
| *MCV* | 1.04 | 1.01,  1.07 |  |  |  |  |  |  |
| *ALT* |  |  | 1.01 | 0.98,  1.05 |  |  |  |  |
| *AST* |  |  |  |  | 1.09 | 1.05,  1.13 |  |  |
| *CDT* |  |  |  |  |  |  | 1.08 | 1.00,  1.17 |

*Notes:* Models with education include 3939 participants. Model 5b-5e are adjusted for age, sex, education, marital status, smoking, BMI, alcohol use and for one of the biomarkers in each case. Values of ALT and AST were divided by 10 and values of MCV and CDT were divided by two, so that the estimates now represent changes per 10-unit or 2-unit increase in the biomarker level. HR = hazard ratio; CI = confidence interval; BMI = body mass index; MCV = mean corpuscular volume; ALT = alanine transaminase; AST = aspartate transaminase; CDT = carbohydrate-deficient transferrin.

**Table I**

*Predictive validity in cox regression models including income.*

| Model | Harrel’s C | Change in Harrel’s C (compared to model 3) | *p* |
| --- | --- | --- | --- |
| Model 3 | 0.863 |  |  |
| Model 4a | 0.863 | < .001 | .894 |
| Model 4b | 0.862 | -.001 | .113 |
| Model 4c | 0.861 | -.002 | .031 |
| Model 4d | 0.862 | -.001 | .651 |
| Model 4e | 0.861 | -.002 | .022 |
|  |  |  |  |
| Model 3 | 0.863 |  |  |
| Model 5a | 0.865 | .002 | .009 |
| Model 5b | 0.863 | <.001 | .227 |
| Model 5c | 0.863 | <.001 | .379 |
| Model 5d | 0.864 | .001 | .080 |
| Model 5e | 0.863 | <.001 | .315 |

*Notes:* All models were adjusted for age, sex, income, marital status, smoking, and BMI. Model 3 was additionally adjusted for alcohol use, models 4a-4e were additionally adjusted for one of the biomarkers in each case (GGT, MCV, ALT, AST, CDT) and models 5a-5e were adjusted for both alcohol use and one of the biomarkers. Models 4a-4e and 5a-5e were compared to model 3; BMI = body mass index; GGT = gamma-glutamyltransferase; MCV = mean corpuscular volume; ALT = alanine transaminase; AST = aspartate transaminase; CDT = carbohydrate-deficient transferrin.

**Table J**

*Risk of death according to alcohol biomarkers in cox regression models 4a-4e with penalized splines.*

|  | Model 4a |  | Model 4b |  | Model 4c |  | Model 4d |  | Model 4e |  |
| --- | --- | --- | --- | --- | --- | --- | --- | --- | --- | --- |
|  | HR | 95% CI | HR | 95% CI | HR | 95% CI | HR | 95% CI | HR | 95% CI |
| Age (t = 1) | 1.11 | 1.10,  1.13 | 1.11 | 1.10,  1.12 | 1.11 | 1.10,  1.12 | 1.11 | 1.10,  1.12 | 1.11 | 1.10,  1.12 |
| Age (t = 2) | 1.01 | 1.00, 1.03 | 1.02 | 1.00, 1.03 | 1.02 | 1.00, 1.03 | 1.01 | 1.00, 1.03 | 1.02 | 1.00, 1.03 |
| Age (t = 3) | 1.02 | 1.00, 1.04 | 1.02 | 1.00, 1.04 | 1.02 | 1.00, 1.04 | 1.02 | 1.00, 1.04 | 1.02 | 1.00, 1.04 |
| Sex |  |  |  |  |  |  |  |  |  |  |
| *Male* | reference |  | reference |  | reference |  | reference |  | reference |  |
| *Female (t = 1)* | 0.46 | 0.35,  0.60 | 0.43 | 0.33,  0.56 | 0.41 | 0.32,  0.54 | 0.42 | 0.32,  0.55 | 0.42 | 0.32,  0.55 |
| *Female (t = 2)* | 1.14 | 0.83, 1.56 | 1.12 | 0.82, 1.54 | 1.13 | 0.82, 1.54 | 1.12 | 0.82, 1.53 | 1.14 | 0.83, 1.56 |
| *Female (t = 3)* | 1.55 | 1.08, 2.23 | 1.54 | 1.07, 2.20 | 1.54 | 1.08, 2.22 | 1.52 | 1.06, 2.18 | 1.56 | 1.09, 2.24 |
| Income (tercile) |  |  |  |  |  |  |  |  |  |  |
| *1 (lowest)* | 1.37 | 1.16,  1.63 | 1.36 | 1.14,  1.61 | 1.37 | 1.15,  1.63 | 1.36 | 1.15,  1.62 | 1.35 | 1.14,  1.61 |
| *2* | 1.06 | 0.91,  1.23 | 1.03 | 0.89,  1.20 | 1.03 | 0.88,  1.20 | 1.04 | 0.89,  1.22 | 1.02 | 0.88,  1.19 |
| *3 (highest)* | reference |  | reference |  | reference |  | reference |  | reference |  |
| Marital status |  |  |  |  |  |  |  |  |  |  |
| *Married* | reference |  | reference |  | reference |  | reference |  | reference |  |
| *Divorced* | 1.23 | 0.95,  1.58 | 1.25 | 0.96,  1.61 | 1.20 | 0.93,  1.56 | 1.21 | 0.94,  1.57 | 1.25 | 0.97,  1.61 |
| *Single* | 1.64 | 1.27,  2.13 | 1.67 | 1.29,  2.17 | 1.71 | 1.32,  2.22 | 1.73 | 1.33,  2.24 | 1.69 | 1.30,  2.19 |
| *Widowed* | 1.19 | 0.99,  1.42 | 1.15 | 0.96,  1.39 | 1.18 | 0.98,  1.42 | 1.16 | 0.97,  1.40 | 1.20 | 0.99,  1.44 |
| Smoking |  |  |  |  |  |  |  |  |  |  |
| *Never smoker* | reference |  | reference |  | reference |  | reference |  | reference |  |
| *Former smoker* | 1.08 | 0.92,  1.27 | 1.06 | 0.90,  1.24 | 1.06 | 0.90,  1.24 | 1.06 | 0.90,  1.24 | 1.08 | 0.92,  1.26 |
| *Current smoker* | 1.90 | 1.57,  2.30 | 1.83 | 1.51,  2.22 | 1.90 | 1.57,  2.30 | 1.88 | 1.55,  2.28 | 1.89 | 1.56,  2.28 |
| BMI (kg/m^2^) | 1.03 | 1.01,  1.04 | 1.03 | 1.02,  1.05 | 1.03 | 1.02,  1.05 | 1.03 | 1.02,  1.05 | 1.03 | 1.02,  1.05 |
|  | *Χ*^2^(df) | *p* | *Χ*^2^(df) | *p* | *Χ*^2^(df) | *p* | *Χ*^2^(df) | *p* | *Χ*^2^(df) | *p* |
| Biomarker |  |  |  |  |  |  |  |  |  |  |
| *GGT* |  |  |  |  |  |  |  |  |  |  |
| Linear | *Χ*^2^(1.00) = 33.69 | < .001 |  |  |  |  |  |  |  |  |
| Non-linear | *Χ*^2^(2.42) = 16.20 | .001 |  |  |  |  |  |  |  |  |
| *MCV* |  |  |  |  |  |  |  |  |  |  |
| Linear |  |  | *Χ*^2^(1.00) = 9.81 | .002 |  |  |  |  |  |  |
| Non-linear |  |  | *Χ*^2^(2.58) = 19.19 | < .001 |  |  |  |  |  |  |
| *ALT* |  |  |  |  |  |  |  |  |  |  |
| Linear |  |  |  |  | *Χ*^2^(1.00) = 16.75 | < .001 |  |  |  |  |
| Non-linear |  |  |  |  | *Χ*^2^(3.95) = 19.93 | < .001 |  |  |  |  |
| *AST* |  |  |  |  |  |  |  |  |  |  |
| Linear |  |  |  |  |  |  | *Χ*^2^(1.00) = 40.99 | < .001 |  |  |
| Non-linear |  |  |  |  |  |  | *Χ*^2^(4.79) = 16.53 | .005 |  |  |
| *CDT* |  |  |  |  |  |  |  |  |  |  |
| Linear |  |  |  |  |  |  |  |  | *Χ*^2^(1.00) = 5.02 | .025 |
| Non-linear |  |  |  |  |  |  |  |  | *Χ*^2^(3.71) = 14.85 | .004 |

*Notes:* Models with income include 3771 participants. Model 4a-4e are adjusted for age, sex, income, marital status, smoking, BMI, and for one of the biomarkers in each case. HR = hazard ratio; CI = confidence interval; BMI = body mass index; GGT = gamma-glutamyltransferase; ALT = alanine transaminase; AST = aspartate transaminase; MCV = mean corpuscular volume; CDT = carbohydrate-deficient transferrin.

**Table K**

*Risk of death according to alcohol biomarkers in cox regression models 5a-5e with penalized splines.*

|  | Model 5a |  | Model 5b |  | Model 5c |  | Model 5d |  | Model 5e |  |
| --- | --- | --- | --- | --- | --- | --- | --- | --- | --- | --- |
|  | HR | 95% CI | HR | 95% CI | HR | 95% CI | HR | 95% CI | HR | 95% CI |
| Age (t = 1) | 1.11 | 1.10,  1.12 | 1.11 | 1.10,  1.12 | 1.11 | 1.10,  1.12 | 1.11 | 1.10,  1.12 | 1.11 | 1.10,  1.12 |
| Age (t = 2) | 1.01 | 1.00, 1.03 | 1.02 | 1.00, 1.03 | 1.02 | 1.00, 1.03 | 1.02 | 1.00, 1.03 | 1.02 | 1.00, 1.03 |
| Age (t = 3) | 1.02 | 1.00, 1.04 | 1.02 | 1.01, 1.04 | 1.02 | 1.01, 1.04 | 1.02 | 1.00, 1.04 | 1.02 | 1.00, 1.04 |
| Sex |  |  |  |  |  |  |  |  |  |  |
| *Male* | reference |  | reference |  | reference |  | reference |  | reference |  |
| *Female (t = 1)* | 0.45 | 0.34,  0.59 | 0.43 | 0.33,  0.56 | 0.41 | 0.31,  0.54 | 0.42 | 0.32,  0.55 | 0.42 | 0.32,  0.55 |
| *Female (t = 2)* | 1.13 | 0.83, 1.55 | 1.12 | 0.82, 1.53 | 1.12 | 0.82, 1.54 | 1.11 | 0.81, 1.52 | 1.13 | 0.83, 1.55 |
| *Female (t = 3)* | 1.54 | 1.07, 2.21 | 1.52 | 1.06, 2.19 | 1.53 | 1.07, 2.20 | 1.51 | 1.05, 2.16 | 1.55 | 1.08, 2.23 |
| Income (tercile) |  |  |  |  |  |  |  |  |  |  |
| *1 (lowest)* | 1.31 | 1.11,  1.56 | 1.31 | 1.10,  1.55 | 1.32 | 1.11,  1.57 | 1.31 | 1.10,  1.56 | 1.30 | 1.09,  1.54 |
| *2* | 1.04 | 0.89,  1.21 | 1.02 | 0.87,  1.18 | 1.01 | 0.87,  1.18 | 1.02 | 0.88,  1.19 | 1.00 | 0.86,  1.17 |
| *3 (highest)* | reference |  | reference |  | reference |  | reference |  | reference |  |
| Marital status |  |  |  |  |  |  |  |  |  |  |
| *Married* | reference |  | reference |  | reference |  | reference |  | reference |  |
| *Divorced* | 1.23 | 0.95,  1.59 | 1.25 | 0.97,  1.62 | 1.21 | 0.94,  1.57 | 1.22 | 0.95,  1.58 | 1.25 | 0.97,  1.62 |
| *Single* | 1.60 | 1.23,  2.08 | 1.62 | 1.25,  2.10 | 1.66 | 1.28,  2.16 | 1.69 | 1.31,  2.20 | 1.64 | 1.27,  2.13 |
| *Widowed* | 1.16 | 0.96,  1.39 | 1.13 | 0.94,  1.35 | 1.15 | 0.96,  1.39 | 1.13 | 0.94,  1.36 | 1.17 | 0.97,  1.40 |
| Smoking |  |  |  |  |  |  |  |  |  |  |
| *Never smoker* | reference |  | reference |  | reference |  | reference |  | reference |  |
| *Former smoker* | 1.10 | 0.93,  1.29 | 1.08 | 0.92,  1.26 | 1.07 | 0.91,  1.25 | 1.08 | 0.92,  1.26 | 1.09 | 0.93,  1.28 |
| *Current smoker* | 1.92 | 1.58,  2.32 | 1.84 | 1.52,  2.23 | 1.90 | 1.57,  2.30 | 1.88 | 1.55,  2.28 | 1.89 | 1.56,  2.30 |
| BMI (kg/m^2^) | 1.02 | 1.01,  1.04 | 1.03 | 1.02,  1.05 | 1.03 | 1.02,  1.05 | 1.03 | 1.01,  1.04 | 1.03 | 1.02,  1.05 |
| Alcohol use |  |  |  |  |  |  |  |  |  |  |
| *Lifetime abstainers* | 1.14 | 0.80,  1.62 | 1.18 | 0.83,  1.68 | 1.14 | 0.80,  1.62 | 1.15 | 0.81,  1.64 | 1.14 | 0.80,  1.62 |
| *Current abstainers* | 1.56 | 1.27,  1.92 | 1.54 | 1.25,  1.89 | 1.56 | 1.27,  1.92 | 1.57 | 1.28,  1.93 | 1.56 | 1.27,  1.92 |
| *Low intake* | reference |  | reference |  | reference |  | reference |  | reference |  |
| *Moderate intake* | 0.83 | 0.69,  1.00 | 0.84 | 0.70,  1.02 | 0.85 | 0.71,  1.03 | 0.84 | 0.70,  1.01 | 0.86 | 0.71,  1.03 |
| *High intake* | 1.12 | 0.87,  1.43 | 1.24 | 0.97,  1.58 | 1.31 | 1.03,  1.67 | 1.22 | 0.95,  1.57 | 1.26 | 0.98,  1.60 |
| Biomarker |  |  |  |  |  |  |  |  |  |  |
|  | *Χ*^2^(df) | *p* | *Χ*^2^(df) | *p* | *Χ*^2^(df) | *p* | *Χ*^2^(df) | *p* | *Χ*^2^(df) | *p* |
| *GGT* |  |  |  |  |  |  |  |  |  |  |
| Linear | *Χ*^2^(1.00) = 32.15 | < .001 |  |  |  |  |  |  |  |  |
| Non-linear | *Χ*^2^(2.38) = 14.91 | .001 |  |  |  |  |  |  |  |  |
| *MCV* |  |  |  |  |  |  |  |  |  |  |
| Linear |  |  | *Χ*^2^(1.00) = 8.38 | .004 |  |  |  |  |  |  |
| Non-linear |  |  | *Χ*^2^(2.58) = 19.35 | < .001 |  |  |  |  |  |  |
| *ALT* |  |  |  |  |  |  |  |  |  |  |
| Linear |  |  |  |  | *Χ*^2^(1.00) = 15.77 | < .001 |  |  |  |  |
| Non-linear |  |  |  |  | *Χ*^2^(3.95) = 21.98 | <.001 |  |  |  |  |
| *AST* |  |  |  |  |  |  |  |  |  |  |
| Linear |  |  |  |  |  |  | *Χ*^2^(1.00) = 41.03 | < .001 |  |  |
| Non-linear |  |  |  |  |  |  | *Χ*^2^(4.97) = 17.26 | .004 |  |  |
| *CDT* |  |  |  |  |  |  |  |  |  |  |
| Linear |  |  |  |  |  |  |  |  | *Χ*^2^(1.00) = 5.55 | .018 |
| Non-linear |  |  |  |  |  |  |  |  | *Χ*^2^(2.56) = 9.76 | .014 |

*Notes:* Models with income include 3771 participants. Model 5b-5e are adjusted for age, sex, income, marital status, smoking, BMI, alcohol use and for one of the biomarkers in each case. HR = hazard ratio; CI = confidence interval; BMI = body mass index; MCV = mean corpuscular volume; ALT = alanine transaminase; AST = aspartate transaminase; CDT = carbohydrate-deficient transferrin.

**Table L**

*Risk of death according to net equivalent income in cox regression models 1, 2, 3 and 5a.*

|  | Model 1 |  | Model 2 |  | Model 3 |  | Model 5a |  |
| --- | --- | --- | --- | --- | --- | --- | --- | --- |
|  | HR | 95% CI | HR | 95% CI | HR | 95% CI | HR | 95% CI |
| Age (t = 1) | 1.10 | 1.09,  1.11 | 1.11 | 1.10,  1.12 | 1.11 | 1.09,  1.12 | 1.11 | 1.10,  1.12 |
| Age (t = 2) | 1.02 | 1.00,  1.03 | 1.02 | 1.00,  1.03 | 1.02 | 1.00,  1.03 | 1.01 | 1.00,  1.03 |
| Age (t = 3) | 1.02 | 1.00,  1.04 | 1.02 | 1.00,  1.04 | 1.02 | 1.00,  1.04 | 1.02 | 1.00,  1.04 |
| Sex |  |  |  |  |  |  |  |  |
| *Male* | reference |  | reference |  | reference |  | reference |  |
| *Female (t = 1)* | 0.43 | 0.33,  0.55 | 0.41 | 0.31,  0.54 | 0.41 | 0.32,  0.54 | 0.43 | 0.33,  0.57 |
| *Female (t = 2)* | 1.17 | 0.85,  1.60 | 1.15 | 0.84,  1.57 | 1.14 | 0.83,  1.57 | 1.13 | 0.83,  1.55 |
| *Female (t = 3)* | 1.59 | 1.11,  2.28 | 1.57 | 1.09,  2.25 | 1.56 | 1.09,  2.24 | 1.53 | 1.07,  2.20 |
| Net equivalent income | 0.95 | 0.94,  0.97 | 0.97 | 0.95,  0.98 | 0.97 | 0.95,  0.99 | 0.97 | 0.95,  0.99 |
| Marital status |  |  |  |  |  |  |  |  |
| *Married* |  |  | reference |  | reference |  | reference |  |
| *Divorced* |  |  | 1.26 | 0.98,  1.62 | 1.26 | 0.98,  1.62 | 1.26 | 0.98,  1.62 |
| *Single* |  |  | 1.69 | 1.30,  2.20 | 1.65 | 1.27,  2.14 | 1.59 | 1.23,  2.07 |
| *Widowed* |  |  | 1.20 | 1.00,  1.44 | 1.17 | 0.97,  1.40 | 1.14 | 0.95,  1.37 |
| Smoking |  |  |  |  |  |  |  |  |
| *Never smoker* |  |  | reference |  | reference |  | reference |  |
| *Former smoker* |  |  | 1.06 | 0.91,  1.25 | 1.08 | 0.92,  1.26 | 1.09 | 0.93,  1.28 |
| *Current smoker* |  |  | 1.94 | 1.61,  2.35 | 1.94 | 1.61,  2.35 | 1.92 | 1.59,  2.33 |
| BMI (kg/m^2^) |  |  | 1.03 | 1.02,  1.05 | 1.03 | 1.01,  1.04 | 1.03 | 1.01,  1.04 |
| Alcohol use |  |  |  |  |  |  |  |  |
| *Lifetime abstainers* |  |  |  |  | 1.12 | 0.79,  1.60 | 1.13 | 0.79,  1.61 |
| *Current abstainers* |  |  |  |  | 1.54 | 1.25,  1.89 | 1.55 | 1.26,  1.91 |
| *Low intake* |  |  |  |  | reference |  | reference |  |
| *Moderate intake* |  |  |  |  | 0.86 | 0.72,  1.04 | 0.85 | 0.71,  1.03 |
| *High intake* |  |  |  |  | 1.32 | 1.04,  1.68 | 1.23 | 0.96,  1.57 |
| GGT |  |  |  |  |  |  | 1.01 | 1.01,  1.02 |

*Notes:* Models with income include 3754 participants. Model 1 is adjusted for age, sex, and net equivalent income; model 2 is model 1 + adjustment for marital status, smoking and BMI; model 3 is model 2 + adjustment for alcohol use; model 5a is model 3 + adjustment for GGT. Net equivalent income was divided by 100 to facilitate interpretation. Values of GGT were divided by 10, so that the estimates now represent changes per 10-unit increase. HR = hazard ratio; CI = confidence interval; BMI = body mass index; GGT = gamma-glutamyltransferase.

**Table M**

*Risk of death according to net equivalent income in cox regression models 4a-4e.*

|  | Model 4a |  | Model 4b |  | Model 4c |  | Model 4d |  | Model 4e |  |
| --- | --- | --- | --- | --- | --- | --- | --- | --- | --- | --- |
|  | HR | 95% CI | HR | 95% CI | HR | 95% CI | HR | 95% CI | HR | 95% CI |
| Age (t = 1) | 1.11 | 1.10,  1.12 | 1.11 | 1.09,  1.12 | 1.11 | 1.10,  1.12 | 1.11 | 1.10,  1.12 | 1.11 | 1.10,  1.12 |
| Age (t = 2) | 1.01 | 1.00,  1.03 | 1.02 | 1.00,  1.03 | 1.02 | 1.00,  1.03 | 1.02 | 1.00,  1.03 | 1.02 | 1.00,  1.03 |
| Age (t = 3) | 1.02 | 1.00,  1.04 | 1.02 | 1.00,  1.04 | 1.02 | 1.00,  1.04 | 1.02 | 1.00,  1.04 | 1.02 | 1.00,  1.04 |
| Sex |  |  |  |  |  |  |  |  |  |  |
| *Male* | reference |  | reference |  | reference |  | reference |  | reference |  |
| *Female (t = 1)* | 0.44 | 0.33,  0.57 | 0.43 | 0.33,  0.56 | 0.42 | 0.32,  0.55 | 0.42 | 0.32,  0.55 | 0.41 | 0.32,  0.54 |
| *Female (t = 2)* | 1.14 | 0.83,  1.56 | 1.14 | 0.83,  1.56 | 1.15 | 0.84,  1.58 | 1.15 | 0.84,  1.57 | 1.15 | 0.84,  1.58 |
| *Female (t = 3)* | 1.54 | 1.08,  2.22 | 1.54 | 1.07,  2.21 | 1.58 | 1.10,  2.27 | 1.57 | 1.10,  2.25 | 1.57 | 1.10,  2.25 |
| Net equivalent income | 0.97 | 0.95,  0.98 | 0.97 | 0.95,  0.99 | 0.97 | 0.95,  0.99 | 0.97 | 0.95,  0.99 | 0.97 | 0.95,  0.99 |
| Marital status |  |  |  |  |  |  |  |  |  |  |
| *Married* | reference |  | reference |  | reference |  | reference |  | reference |  |
| *Divorced* | 1.25 | 0.97,  1.62 | 1.28 | 0.99,  1.65 | 1.25 | 0.97,  1.62 | 1.25 | 0.97,  1.61 | 1.26 | 0.98,  1.62 |
| *Single* | 1.64 | 1.26,  2.13 | 1.70 | 1.31,  2.20 | 1.68 | 1.30,  2.19 | 1.69 | 1.30,  2.19 | 1.69 | 1.31,  2.20 |
| *Widowed* | 1.17 | 0.97,  1.40 | 1.19 | 0.99,  1.43 | 1.19 | 0.99,  1.43 | 1.18 | 0.99,  1.42 | 1.20 | 1.00,  1.44 |
| Smoking |  |  |  |  |  |  |  |  |  |  |
| *Never smoker* | reference |  | reference |  | reference |  | reference |  | reference |  |
| *Former smoker* | 1.07 | 0.91,  1.26 | 1.06 | 0.91,  1.25 | 1.06 | 0.91,  1.25 | 1.06 | 0.91,  1.25 | 1.07 | 0.91,  1.25 |
| *Current smoker* | 1.91 | 1.58,  2.31 | 1.86 | 1.54,  2.26 | 1.96 | 1.62,  2.37 | 1.97 | 1.63,  2.38 | 1.94 | 1.61,  2.35 |
| BMI (kg/m^2^) | 1.03 | 1.01,  1.04 | 1.03 | 1.02,  1.05 | 1.03 | 1.01,  1.04 | 1.03 | 1.01,  1.04 | 1.03 | 1.02,  1.05 |
| Biomarker |  |  |  |  |  |  |  |  |  |  |
| *GGT* | 1.01 | 1.01,  1.02 |  |  |  |  |  |  |  |  |
| *MCV* |  |  | 1.05 | 1.01,  1.08 |  |  |  |  |  |  |
| *ALT* |  |  |  |  | 1.03 | 0.99,  1.07 |  |  |  |  |
| *AST* |  |  |  |  |  |  | 1.09 | 1.05,  1.13 |  |  |
| *CDT* |  |  |  |  |  |  |  |  | 1.06 | 0.98,  1.15 |

*Notes:* Models with income include 3754 participants. Model 4a-4e are adjusted for age, sex, net equivalent income, marital status, smoking, BMI, and for one of the biomarkers in each case. Net equivalent income was divided by 100 to facilitate interpretation. Values of GGT, ALT and AST were divided by 10 and values of MCV and CDT were divided by two, so that the estimates now represent changes per 10-unit or 2-unit increase in the biomarker level. HR = hazard ratio; CI = confidence interval; BMI = body mass index; GGT = gamma-glutamyltransferase; ALT = alanine transaminase; AST = aspartate transaminase; MCV = mean corpuscular volume; CDT = carbohydrate-deficient transferrin.

**Table N**

*Risk of death according to net equivalent income in cox regression models 5b-5e.*

|  | Model 5b |  | Model 5c |  | Model 5d |  | Model 5e |  |
| --- | --- | --- | --- | --- | --- | --- | --- | --- |
|  | HR | 95% CI | HR | 95% CI | HR | 95% CI | HR | 95% CI |
| Age (t = 1) | 1.11 | 1.09,  1.12 | 1.11 | 1.10,  1.12 | 1.11 | 1.10,  1.12 | 1.11 | 1.09,  1.12 |
| Age (t = 2) | 1.02 | 1.00,  1.03 | 1.02 | 1.00,  1.03 | 1.02 | 1.00,  1.03 | 1.02 | 1.00,  1.03 |
| Age (t = 3) | 1.02 | 1.00,  1.04 | 1.02 | 1.00,  1.04 | 1.02 | 1.00,  1.04 | 1.02 | 1.00,  1.04 |
| Sex |  |  |  |  |  |  |  |  |
| *Male* | reference |  | reference |  | reference |  | reference |  |
| *Female (t = 1)* | 0.43 | 0.32,  0.56 | 0.42 | 0.32,  0.55 | 0.42 | 0.32,  0.55 | 0.41 | 0.31,  0.54 |
| *Female (t = 2)* | 1.13 | 0.83,  1.55 | 1.15 | 0.84,  1.57 | 1.14 | 0.84,  1.57 | 1.15 | 0.84,  1.57 |
| *Female (t = 3)* | 1.53 | 1.07,  2.19 | 1.57 | 1.09,  2.25 | 1.56 | 1.09,  2.24 | 1.56 | 1.09,  2.24 |
| Net equivalent income | 0.97 | 0.95,  0.99 | 0.97 | 0.95,  0.99 | 0.97 | 0.96,  0.99 | 0.97 | 0.96,  0.99 |
| Marital status |  |  |  |  |  |  |  |  |
| *Married* | reference |  | reference |  | reference |  | reference |  |
| *Divorced* | 1.28 | 0.99,  1.65 | 1.26 | 0.97,  1.62 | 1.25 | 0.97,  1.62 | 1.26 | 0.98,  1.63 |
| *Single* | 1.65 | 1.27,  2.14 | 1.64 | 1.27,  2.13 | 1.65 | 1.27,  2.14 | 1.65 | 1.27,  2.14 |
| *Widowed* | 1.16 | 0.97,  1.39 | 1.16 | 0.97,  1.39 | 1.15 | 0.96,  1.38 | 1.17 | 0.97,  1.40 |
| Smoking |  |  |  |  |  |  |  |  |
| *Never smoker* | reference |  | reference |  | reference |  | reference |  |
| *Former smoker* | 1.08 | 0.92,  1.27 | 1.08 | 0.92,  1.27 | 1.08 | 0.92,  1.27 | 1.08 | 0.92,  1.27 |
| *Current smoker* | 1.88 | 1.55,  2.28 | 1.96 | 1.62,  2.37 | 1.97 | 1.63,  2.39 | 1.94 | 1.61,  2.36 |
| BMI (kg/m^2^) | 1.03 | 1.01,  1.04 | 1.03 | 1.01,  1.04 | 1.03 | 1.01,  1.04 | 1.03 | 1.01,  1.04 |
| Alcohol use |  |  |  |  |  |  |  |  |
| *Lifetime abstainers* | 1.14 | 0.80,  1.62 | 1.11 | 0.78,  1.59 | 1.12 | 0.79,  1.59 | 1.15 | 0.80,  1.63 |
| *Current abstainers* | 1.51 | 1.22,  1.85 | 1.53 | 1.25,  1.88 | 1.54 | 1.25,  1.90 | 1.57 | 1.27,  1.93 |
| *Low intake* | reference |  | reference |  | reference |  | reference |  |
| *Moderate intake* | 0.84 | 0.70,  1.01 | 0.86 | 0.71,  1.03 | 0.85 | 0.70,  1.02 | 0.85 | 0.71,  1.03 |
| *High intake* | 1.27 | 0.99,  1.61 | 1.30 | 1.02,  1.66 | 1.24 | 0.97,  1.58 | 1.28 | 1.01,  1.63 |
| Biomarker |  |  |  |  |  |  |  |  |
| *MCV* | 1.04 | 1.01,  1.07 |  |  |  |  |  |  |
| *ALT* |  |  | 1.02 | 0.98,  1.06 |  |  |  |  |
| *AST* |  |  |  |  | 1.09 | 1.05,  1.13 |  |  |
| *CDT* |  |  |  |  |  |  | 1.08 | 0.99,  1.17 |

*Notes:* Models with income include 3754 participants. Model 5b-5e are adjusted for age, sex, net equivalent income, marital status, smoking, BMI, alcohol use and for one of the biomarkers in each case. Net equivalent income was divided by 100 to facilitate interpretation. Values of ALT and AST were divided by 10 and values of MCV and CDT were divided by two, so that the estimates now represent changes per 10-unit or 2-unit increase in the biomarker level. HR = hazard ratio; CI = confidence interval; BMI = body mass index; MCV = mean corpuscular volume; ALT = alanine transaminase; AST = aspartate transaminase; CDT = carbohydrate-deficient transferrin.

**Table O**

*Comparison of models 5a with and without penalized spline for incomes.*

|  | Model 5a with net equivalent income | | Model 5a using splines for net equivalent income | | Loglikelihood-ratio-test for both models | |
| --- | --- | --- | --- | --- | --- | --- |
|  |  | |  | | *χ*²(df) | *p* |
|  | HR | 95% CI | HR | 95% CI | *χ*²(0.01) = 0.02 | .022 |
| Age (t = 1) | 1.11 | 1.10,  1.12 | 1.11 | 1.10,  1.12 |  | |
| Age (t = 2) | 1.01 | 1.00,  1.03 | 1.01 | 1.00, 1.03 |  |  |
| Age (t = 3) | 1.02 | 1.00,  1.04 | 1.02 | 1.00, 1.04 |  |  |
| Sex |  |  |  |  |  |  |
| *Male* | reference |  | reference |  |  |  |
| *Female (t = 1)* | 0.43 | 0.33,  0.57 | 0.43 | 0.33,  0.57 |  |  |
| *Female (t = 2)* | 1.13 | 0.83,  1.55 | 1.13 | 0.83, 1.55 |  |  |
| *Female (t = 3)* | 1.53 | 1.07,  2.20 | 1.53 | 1.07, 2.20 |  |  |
| Net equivalent income | 0.97 | 0.95,  0.99 |  |  |  |  |
| *Linear* |  |  | *Χ*^2^(1.00) = 10.13 | *p* = .002 |  |  |
| *Non-linear* |  |  | *Χ*^2^(0.01) = 0.01 | *p* = .720 |  |  |
| Marital status |  |  |  |  |  |  |
| *Married* | reference |  | reference |  |  |  |
| *Divorced* | 1.26 | 0.98,  1.62 | 1.26 | 0.97,  1.62 |  |  |
| *Single* | 1.59 | 1.23,  2.07 | 1.59 | 1.23,  2.07 |  |  |
| *Widowed* | 1.14 | 0.95,  1.37 | 1.14 | 0.95,  1.37 |  |  |
| Smoking |  |  |  |  |  |  |
| *Never smoker* | reference |  | reference |  |  |  |
| *Former smoker* | 1.09 | 0.93,  1.28 | 1.09 | 0.93,  1.28 |  |  |
| *Current smoker* | 1.92 | 1.59,  2.33 | 1.92 | 1.59,  2.33 |  |  |
| BMI (kg/m^2^) | 1.03 | 1.01,  1.04 | 1.03 | 1.01,  1.04 |  |  |
| Alcohol use |  |  |  |  |  |  |
| *Lifetime abstainers* | 1.13 | 0.79,  1.61 | 1.13 | 0.79,  1.61 |  |  |
| *Current abstainers* | 1.55 | 1.26,  1.91 | 1.55 | 1.26,  1.91 |  |  |
| *Low intake* | reference |  | reference |  |  |  |
| *Moderate intake* | 0.85 | 0.71,  1.03 | 0.85 | 0.71,  1.03 |  |  |
| *High intake* | 1.23 | 0.96,  1.57 | 1.23 | 0.96,  1.57 |  |  |
| Biomarker |  |  |  |  |  |  |
| *GGT* | 1.01 | 1.01,  1.02 | 1.01 | 1.01,  1.02 |  | |

*Notes:* Models include 3754 participants. Model 5b-5e are adjusted for age, sex, income, marital status, smoking, BMI, alcohol use and for one of the biomarkers in each case. Values of GGT were divided by 10, so that the estimates now represent changes per 10-unit increase. HR = hazard ratio; CI = confidence interval; BMI = body mass index; MCV = mean corpuscular volume; ALT = alanine transaminase; AST = aspartate transaminase; CDT = carbohydrate-deficient transferrin.

**Table P**

*Risk of death according to alcohol use in sensitivity analyses using cox regression models 3 with age and sex as time-varying variables and income (tercile) as SES proxy*

|  | (a) analysis for the younger part of the cohort (aged < 55 years at baseline) | (b) exclusion of individuals with pre-existing health conditions (pcs and/or mcs scores in SF12 > 40), | (c) no control of smoking status | (d) no control of income or education | (e) HED as an alternative alcohol exposure | (f) control of health-related quality of life as pcs and mcs scores in SF12 | (g) control of depressive symptoms |
| --- | --- | --- | --- | --- | --- | --- | --- |
| Total participants (*N*) | 2320 | 2700 | 3771 | 3771 | 3771 | 3571 | 3752 |
|  | HR (95% CI) | HR (95% CI) | HR (95% CI) | HR (95% CI) | HR (95% CI) | HR (95% CI) | HR (95% CI) |
| Alcohol use |  |  |  |  |  |  |  |
| *Lifetime abstainers* | 0.35 (0.05, 2.54) | 1.28 (0.79, 2.08) | 1.11 (0.78, 1.58) | 1.15 (0.81, 1.63) |  | 1.32 (0.92, 1.91) | 1.10 (0.77, 1.57) |
| *Current abstainers* | 0.84 (0.38, 1.85) | 1.59 (1.20, 2.12) | 1.59 (1.29, 1.96) | 1.61 (1.31, 1.97) |  | 1.49 (1.21, 1.84) | 1.56 (1.27, 1.92) |
| *Low intake* | reference | reference | reference | reference |  | reference | reference |
| *Moderate intake* | 0.75 (0.50, 1.11) | 0.95 (0.75, 1.20) | 0.88 (0.73, 1.07) | 0.86 (0.71, 1.03) |  | 0.89 (0.74, 1.09) | 0.86 (0.71, 1.03) |
| *High intake* | 1.13 (0.74, 1.72) | 1.32 (0.97, 1.78) | 1.37 (1.08, 1.74) | 1.38 (1.09, 1.75) |  | 1.41 (1.10, 1.80) | 1.28 (1.01, 1.63) |
| HED |  |  |  |  |  |  |  |
| *No* |  |  |  |  | reference |  |  |
| *Yes* |  |  |  |  | 0.94 (0.80, 1.11) |  |  |

*Notes:* Models include sex, age, income, marital status, smoking, BMI and self-reported alcohol use if not otherwise stated. Model e includes HED instead of alcohol use. HED = heavy episodic drinking; HR = hazard ratio; CI = confidence interval; PCS = physical component summary; MCS = mental component summary; BMI = body mass index.

**Table Q**

*Risk of death according to interactions of education and alcohol use in cox regression models and aalen additive models*

|  | *Cox proportional hazards regression* | | | | | Aalen additive hazards regression | | | | | |
| --- | --- | --- | --- | --- | --- | --- | --- | --- | --- | --- | --- |
|  | *Model output* | | | Reparametrization | | Model output | | | Reparametrization | | |
|  | *N* | HR | 95% CI | HR | 95% CI | Coefficient | 95% CI | Additional cases per 10,000-person years | Coefficient | 95% CI | Additional cases per 10,000-person years |
| Education |  |  |  |  |  |  |  |  |  |  |  |
| *< 10 years* | 1532 | 1.51 | 1.15, 1.99 |  |  | 8.58e-06 | -1.02e-06, 1.82e-05 | 31.34 |  |  |  |
| *10 years* | 1735 | 1.19 | 0.87, 1.63 |  |  | 3.92e-06 | -1.98e-06, 9.82e-06 | 14.32 |  |  |  |
| *> 10 years* | 672 | reference |  |  |  | reference |  |  |  |  |  |
| Alcohol use |  |  |  |  |  |  |  |  |  |  |  |
| *Lifetime abstainers* | 102 | 0.67 | 0.09, 4.85 |  |  | -6.73e-06 | -6.24e-05, 4.89e-05 | -24.58 |  |  |  |
| *Current abstainers* | 220 | 1.40 | 0.60, 3.26 |  |  | 2.27e-05 | -3.96e-05, 8.50e-05 | 82.91 |  |  |  |
| *Low intake* | 2431 | reference |  |  |  | reference |  |  |  |  |  |
| *Moderate intake* | 831 | 0.80 | 0.50, 1.28 |  |  | -1.47e-05 | -2.34e-05, -5.96e-06 | -53.69 |  |  |  |
| *High intake* | 355 | 1.22 | 0.68, 2.19 |  |  | -1.03e-05 | -2.57e-05, 5.09e-06 | -37.62 |  |  |  |
| Interactions |  |  |  |  |  |  |  |  |  |  |  |
| *< 10 years*, lifetime abstainer | 76 | 1.69 | 0.23, 12.63 | 1.71 | 1.11, 2.65 | 1.01e-06 | -5.99e-05, 6.20e-05 | 3.69 | 2.86e-06 | -7.281e-05, 7.853e-05 | 10.45 |
| *10 years*, lifetime abstainer | 21 | 0.67 | 0.04, 10.98 | 0.54 | 0.07, 3.89 | -3.81e-06 | -6.08e-05, 5.32e-05 | -13.92 | -6.62e-06 | -8.439e-05, 7.115e-05 | -24.18 |
| *< 10 years, current abstainer* | 153 | 1.09 | 0.46, 2.60 | 2.31 | 1.67, 3.19 | 3.43e-05 | -3.39e-05, 1.03e-04 | 125.28 | 6.564e-05 | -1.892e-05, 0.00015021 | 239.77 |
| *10 years, current abstainer* | 54 | 1.04 | 0.36, 2.96 | 1.73 | 0.91, 3.29 | -1.71e-05 | -8.22e-05, 4.80e-05 | -62.46 | 9.51e-06 | -7.679e-05, 9.581e-05 | 34.74 |
| *< 10 years, moderate intake* | 232 | 1.04 | 0.62, 1.74 | 1.26 | 0.91, 1.74 | 4.08e-07 | -1.70e-05, 1.78e-05 | 1.49 | -5.76e-06 | -1.185e-05, 3.3e-07 | -21.05 |
| *10 years, moderate intake* | 402 | 1.18 | 0.66, 2.10 | 1.12 | 0.76, 1.67 | 3.20e-06 | -6.70e-06, 1.31e-05 | 11.69 | -7.63e-06 | -1.794e-05, 2.67e-06 | -27.88 |
| *< 10 years, high intake* | 102 | 1.19 | 0.62, 2.28 | 2.19 | 1.50, 3.20 | 2.26e-05 | -6.21e-06, 5.14e-05 | 82.55 | 2.088e-05 | 1.936e-05, 2.239e-05 | 76.25 |
| *10 years, high intake* | 185 | 1.09 | 0.52, 2.29 | 1.59 | 0.96, 2.61 | 1.99e-06 | -1.49e-05, 1.89e-05 | 7.27 | -4.35e-06 | -2.425e-05, 1.555e-05 | -15.89 |
| *>10 years, low intake* | 389 | reference |  |  |  | reference |  | reference |  |  |  |

*Notes:* Models are adjusted for age, sex, and marital status. To simplify the interpretation of interaction effects, we reparametrized HR and coefficients from the model outputs, i.e. products of HR of respective main and interaction effects (CPHR) and sums of coefficients of respective main and interaction effects (AAHR) were computed. HR = hazard ratio; CI = confidence interval.
