# Supplementary material for: Socioeconomic Status, Alcohol Use, and Mortality: Results From a Prospective Cohort Study
Source: Alcohol Clin Exp Res (Hoboken). 2026 Sep 5;50(9):e70402. doi: 10.1111/acer.70402 (PMC13545546; doi:10.1111/acer.70402)
Supplement: Supplementary file 3 [file ACER-50-0-s003.docx]

**Supplementary Material B**

**Tables**

**Table A:** *Frequency of missing data, stratified by sex.*

**Table B:** *Descriptive statistics by income tercile, stratified by sex.*

**Table C:** *Descriptive statistics by education group, stratified by sex.*

**Table D:** *Risk of death according to income (tercile) in cox regression models 1, 2, 3, and 5a for women.*

**Table E:** *Risk of death according to income (tercile) in cox regression models 4a-4e for women.*

**Table F:** *Risk of death according to income (tercile) in cox regression models 5b-5e for women.*

**Table G:** *Risk of death according to income (tercile) in cox regression models 1, 2, 3, and 5a for men.*

**Table H:** *Risk of death according to income (tercile) in cox regression models 4a-4e for men.*

**Table I:** *Risk of death according to income (tercile) in cox regression models 5b-5e for men.*

**Table J:** *Risk of death according to education in cox regression models 1, 2, 3, and 5a for women.*

**Table K:** *Risk of death according to education in cox regression models 4a-4e for women.*

**Table L:** *Risk of death according to education in cox regression models 5b-5e for women.*

**Table M:** *Risk of death according to education in cox regression models 1, 2, 3, and 5a for men.*

**Table N:** *Risk of death according to education in cox regression models 4a-4e for men.*

**Table O:** *Risk of death according to education in cox regression models 5b-5e for men.*

**Table A**

*Frequency of missing data.*

|  | **Women** |  | **Men** |  |
| --- | --- | --- | --- | --- |
| Variable | *N* (missing) | *N* (available) | *N* (missing) | *N* (available) |
| Death | 0 | 2192 | 0 | 2115 |
| Age | 0 | 2192 | 0 | 2115 |
| Income | 125 | 2066 | 126 | 1989 |
| Education | 15 | 2176 | 15 | 2100 |
| SF-12 |  |  |  |  |
| *PCS* | 151 | 2040 | 168 | 1947 |
| *MCS* | 151 | 2040 | 168 | 1947 |
| BMI (kg/m^2^) | 5 | 2186 | 5 | 2110 |
| Smoking | 8 | 2183 | 11 | 2104 |
| Alcohol use | 127 | 2064 | 141 | 1974 |
| Biomarker |  |  |  |  |
| *GGT (U/l)* | 33 | 2158 | 18 | 2097 |
| *ALT (U/l)* | 15 | 2176 | 15 | 2100 |
| *AST (U/l)* | 11 | 2180 | 10 | 2105 |
| *MCV (fl)* | 12 | 2179 | 5 | 2110 |
| *CDT (%)* | 8 | 2183 | 8 | 2107 |

*Notes:* Using income as a SES proxy, 536 (12.4%) were excluded due to missing data in any of the exposure or confounder variables included in models 5a-e, resulting in a final study population of 3771 individuals. Using education as an SES proxy, 368 (8.5%) were excluded due to missing data resulting in a final study population of 3939 individuals.

**Table B**

*Descriptive statistics by income tercile, stratified by sex.*

|  | **Women** |  |  |  | **Men** |  |  |  |
| --- | --- | --- | --- | --- | --- | --- | --- | --- |
|  |  | Income (terciles) |  |  |  | Income (terciles) |  |  |
|  | Full sample | First (lowest) | Second | Third (highest) | Full Sample | First (lowest) | Second | Third (highest) |
| Total participants (*N*) | 1925 | 662 | 716 | 547 | 1846 | 574 | 593 | 679 |
| Deaths (% died) | 19.3% | 15.9% | 24.3% | 17% | 33% | 31% | 35.2% | 32.7% |
| Age (in years) | 48.24 (16.05) | 44.16 (16.27) | 51.29 (16.38) | 49.2 (14.22) | 50.07 (16.6) | 43.88 (16.38) | 52.72 (16.96) | 53.00 (14.99) |
| SF-12 |  |  |  |  |  |  |  |  |
| *PCS* | 47.79 (9.22) | 48.35 (9.23) | 46.91 (9.37) | 48.27 (8.94) | 48.57 (8.51) | 48.83 (8.4) | 47.77 (8.74) | 49.02 (8.36) |
| *MCS* | 50.61 (8.83) | 49.45 (9.05) | 51.25 (8.53) | 51.15 (8.83) | 53.23 (7.42) | 52.18 (8.1) | 53.61 (7.43) | 53.8 (6.69) |
| BMI (kg/m^2^) | 26.83 (5.3) | 27.06 (5.58) | 27.17 (5.34) | 26.1 (4.83) | 27.62 (4.08) | 27.02 (4.4) | 28.06 (4.06) | 27.75 (3.74) |
| Smoking |  |  |  |  |  |  |  |  |
| *Never smoker (%)* | 49.6% | 42.1% | 54.7% | 51.7% | 21.8% | 19.7% | 20.6% | 24.6% |
| *Former smoker (%)* | 22.9% | 19.6% | 22.1% | 28.0% | 44.5% | 32.4% | 48.6% | 51.3% |
| *Current smoker (%)* | 27.5% | 38.2% | 23.2% | 20.3% | 33.7% | 47.9% | 30.9% | 24.2% |
| Alcohol use |  |  |  |  |  |  |  |  |
| *Lifetime abstainers (%)* | 4.0% | 5.1% | 4.3% | 2.2% | 1.1% | 2.1% | 1.2% | 0.3% |
| *Current abstainers (%)* | 5.1% | 6.8% | 5.7% | 2.4% | 6.0% | 7.8% | 5.7% | 4.6% |
| *Low intake (%)* | 76.3% | 75.7% | 76.3% | 77.1% | 45.7% | 45.1% | 47.9% | 44.2% |
| *Moderate intake (%)* | 10.6% | 8.6% | 10.6% | 13.0% | 32.4% | 27.4% | 33.2% | 36.1% |
| *High intake (%)* | 3.9% | 3.8% | 3.1% | 5.3% | 14.8% | 17.6% | 12.0% | 14.9% |
| Biomarker |  |  |  |  |  |  |  |  |
| *GGT (U/l)* | 22.6 (32.43) | 22.8 (37.27) | 22.02 (27.37) | 23.12 (32.29) | 48.72 (104.86) | 55.83 (132.35) | 43.96 (80.41) | 46.86 (97.00) |
| *MCV (fl)* | 89.53 (4.53) | 89.49 (5.03) | 89.39 (3.98) | 89.77 (4.56) | 90.69 (4.41) | 90.81 (4.5) | 90.85 (4.42) | 90.44 (4.33) |
| *ALT (U/l)* | 22.16 (13.75) | 23.06 (16.94) | 21.58 (11.56) | 21.85 (11.95) | 35.19 (23.94) | 36.58 (22.37) | 34.26 (24.58) | 34.82 (24.62) |
| *AST (U/l)* | 19.35 (8.99) | 19.62 (11.76) | 19.26 (6.81) | 19.14 (7.53) | 25.19 (16.8) | 26.24 (14.86) | 24.8 (20.68) | 24.64 (14.39) |
| *CDT (%)* | 4.69 (1.48) | 4.69 (1.57) | 4.7 (1.46) | 4.7 (1.4) | 4.94 (1.74) | 5.17 (2.1) | 4.84 (1.68) | 4.82 (1.42) |

*Notes:* Numbers display absolute values (*N*), percentages (*%*) or mean (standard deviation in brackets). PCS = physical component summary; MCS = mental component summary; BMI = body mass index; GGT = gamma-glutamyltransferase; ALT = alanine transaminase; AST = aspartate transaminase; MCV = mean corpuscular volume; CDT = carbohydrate-deficient transferrin.

**Table C**

*Descriptive statistics by education group, stratified by sex.*

|  | **Women** |  |  |  | **Men** |  |  |  |
| --- | --- | --- | --- | --- | --- | --- | --- | --- |
|  |  | Education |  |  |  | Education |  |  |
|  | Full sample | < 10 years | 10 years | > years | Full sample | < 10 years | 10 years | > 10 years |
| Total participants (*N*) | 2010 | 745 | 940 | 325 | 1929 | 787 | 795 | 347 |
| Deaths | 20.0% | 41.5% | 7.3% | 7.1% | 33.5% | 55.3% | 16.2% | 23.6% |
| Age | 48.59 (16.12) | 61.86 (11.86) | 40.75 (12.28) | 40.83 (14.42) | 50.31 (16.55) | 60.13 (13.81) | 42.07 (13.86) | 46.92 (16.28) |
| SF-12 |  |  |  |  |  |  |  |  |
| *PCS* | 47.76 (9.22) | 43.48 (10.23) | 49.82 (7.82) | 51.08 (6.94) | 48.53 (8.56) | 45.8 (9.29) | 50.04 (7.54) | 51.03 (7.41) |
| *MCS* | 50.61 (8.84) | 51.42 (9.13) | 50.48 (8.6) | 49.23 (8.71) | 53.32 (7.38) | 53.91 (7.86) | 53.41 (6.64) | 51.81 (7.74) |
| BMI (kg/m^2^) | 26.85 (5.3) | 28.94 (5.21) | 25.89 (5.13) | 24.84 (4.37) | 27.62 (4.06) | 28.28 (4.2) | 27.33 (3.88) | 26.82 (3.93) |
| Smoking |  |  |  |  |  |  |  |  |
| *Never smoker (%)* | 49.9% | 64.4% | 38.0% | 50.8% | 21.4% | 14.7% | 22.3% | 34.3% |
| *Former smoker (%)* | 22.7% | 17.7% | 25.5% | 26.2% | 45.0% | 55.9% | 36.1% | 40.6% |
| *Current smoker (%)* | 27.4% | 17.9% | 36.5% | 23.1% | 33.6% | 29.4% | 41.6% | 25.1% |
| Alcohol use |  |  |  |  |  |  |  |  |
| *Lifetime abstainers (%)* | 4.0% | 8.6% | 1.7% | 0.3% | 1.1% | 1.5% | 0.6% | 1.2% |
| *Current abstainers (%)* | 5.2% | 10.1% | 2.7% | 1.2% | 6.0% | 9.9% | 3.6% | 2.6% |
| *Low intake (%)* | 76.6% | 74.5% | 79.5% | 73.2% | 46.2% | 52.6% | 41.0% | 43.5% |
| *Moderate intake (%)* | 10.3% | 5.0% | 11.7% | 18.8% | 32.2% | 24.8% | 36.7% | 39.2 |
| *High intake (%)* | 3.8% | 1.9% | 4.5% | 6.5% | 14.4% | 11.2% | 18.0% | 13.5% |
| Biomarker |  |  |  |  |  |  |  |  |
| *GGT (U/l)* | 22.45 (32.07) | 27.72 (44.85) | 19.17 (18.06) | 19.86 (26.64) | 48.3 (103.46) | 47.16 (90.92) | 52.5 (119.66) | 41.29 (88.98) |
| *MCV (fl)* | 89.55 (4.49) | 89.61 (4.5) | 89.66 (4.54) | 89.1 (4.30) | 90.69 (4.41) | 91.25 (4.71) | 90.25 (4.08) | 90.43 (4.28) |
| *ALT (U/l)* | 22.2 (13.75) | 25.16 (15.48) | 20.77 (13.01) | 19.58 (9.90) | 35.11 (23.72) | 31.47 (21.63) | 39.7 (26.93) | 32.86 (17.98) |
| *AST (U/l)* | 19.39 (8.95) | 21.23 (12.00) | 18.36 (6.67) | 18.12 (4.93) | 25.09 (16.65) | 23.97 (12.96) | 26.79 (21.55) | 23.73 (9.22) |
| *CDT (%)* | 4.7 (1.49) | 4.64 (1.41) | 4.74 (1.60) | 4.72 (1.32) | 4.94 (1.77) | 4.86 (1.88) | 5.08 (1.82) | 4.79 (1.34) |

*Notes:* Numbers display absolute values (*N*), percentages (*%*) or mean (standard deviation in brackets). PCS = physical component summary; MCS = mental component summary; BMI = body mass index; GGT = gamma-glutamyltransferase; ALT = alanine transaminase; AST = aspartate transaminase; MCV = mean corpuscular volume; CDT = carbohydrate-deficient transferrin.

**Table D**

*Risk of death according to income (tercile) in cox regression models 1, 2, 3, and 5a for women.*

|  | Model 1 |  | Model 2 |  | Model 3 |  | Model 5a |  |
| --- | --- | --- | --- | --- | --- | --- | --- | --- |
|  | HR | 95% CI | HR | 95% CI | HR | 95% CI | HR | 95% CI |
| Age (t = 1) | 1.13 | 1.11,  1.16 | 1.14 | 1.11,  1.16 | 1.13 | 1.11,  1.16 | 1.13 | 1.11,  1.16 |
| Age (t = 2) | 1.00 | 0.97,  1.03 | 1.00 | 0.97,  1.03 | 1.00 | 0.97,  1.03 | 1.00 | 0.97,  1.03 |
| Age (t = 3) | 1.00 | 0.97,  1.03 | 1.00 | 0.97,  1.03 | 1.00 | 0.97,  1.03 | 1.00 | 0.97,  1.03 |
| Income (tercile) |  |  |  |  |  |  |  |  |
| *1 (lowest)* | 1.17 | 0.88,  1.54 | 1.14 | 0.85,  1.54 | 1.10 | 0.82,  1.49 | 1.11 | 0.82, 1.50 |
| *2* | 1.08 | 0.84,  1.39 | 1.06 | 0.81,  1.37 | 1.03 | 0.80,  1.34 | 1.07 | 0.82,  1.39 |
| *3 (highest)* | reference |  | reference |  | reference |  | reference |  |
| Marital status |  |  |  |  |  |  |  |  |
| *Married* | reference |  | reference |  | reference |  | reference |  |
| *Divorced* |  |  | 0.84 | 0.58,  1.23 | 0.87 | 0.59,  1.27 | 0.86 | 0.59,  1.26 |
| *Single* |  |  | 1.14 | 0.77,  1.68 | 1.15 | 0.77,  1.70 | 1.13 | 0.76,  1.67 |
| *Widowed* |  |  | 0.96 | 0.74,  1.25 | 0.96 | 0.74,  1.24 | 0.96 | 0.74,  1.25 |
| Smoking |  |  |  |  |  |  |  |  |
| *Never smoker* | reference |  | reference |  | reference |  | reference |  |
| *Former smoker* |  |  | 1.18 | 0.92,  1.52 | 1.20 | 0.93,  1.54 | 1.20 | 0.94, 1.55 |
| *Current smoker* |  |  | 1.56 | 1.14,  2.14 | 1.55 | 1.13,  2.12 | 1.59 | 1.16,  2.18 |
| BMI (kg/m^2^) |  |  | 1.03 | 1.01,  1.05 | 1.02 | 1.00,  1.04 | 1.02 | 1.00,  1.04 |
| Alcohol use |  |  |  |  |  |  |  |  |
| *Lifetime abstainers* |  |  |  |  | 1.26 | 0.84,  1.91 | 1.24 | 0.82,  1.87 |
| *Current abstainers* |  |  |  |  | 1.57 | 1.14,  2.16 | 1.56 | 1.13,  2.15 |
| *Low intake* | reference |  | reference |  | reference |  | reference |  |
| *Moderate intake* |  |  |  |  | 0.74 | 0.43,  1.28 | 0.69 | 0.39,  1.20 |
| *High intake* |  |  |  |  | 1.30 | 0.66,  2.55 | 1.30 | 0.66,  2.56 |
| Biomarker |  |  |  |  |  |  |  |  |
| *GGT* |  |  |  |  |  |  | 1.04 | 1.02,  1.06 |

*Notes:* Models with income include 1925 female participants. Model 1 is adjusted for age, sex, and income; model 2 is model 1 + adjustment for marital status, smoking and BMI; model 3 is model 2 + adjustment for alcohol use; model 5a is model 3 + adjustment for GGT. Values of GGT were divided by 10, so that the estimates now represent changes per 10-unit increase. HR = hazard ratio; CI = confidence interval; BMI = body mass index; GGT = gamma-glutamyltransferase.

**Table E**

*Risk of death according to income (tercile) in cox regression models 4a-4e for women.*

|  | Model 4a |  | Model 4b |  | Model 4c |  | Model 4d |  | Model 4e |  |
| --- | --- | --- | --- | --- | --- | --- | --- | --- | --- | --- |
|  | Harrel’s C: |  | Harrel’s C: |  | Harrel’s C: |  | Harrel’s C: |  | Harrel’s C: |  |
|  | HR | 95% CI | HR | 95% CI | HR | 95% CI | HR | 95% CI | HR | 95% CI |
| Age (t = 1) | 1.14 | 1.11,  1.17 | 1.14 | 1.11,  1.16 | 1.14 | 1.11,  1.16 | 1.14 | 1.11,  1.16 | 1.14 | 1.11,  1.16 |
| Age (t = 2) | 1.00 | 0.97,  1.03 | 1.00 | 0.97,  1.03 | 1.00 | 0.97,  1.03 | 1.00 | 0.97, 1.03 | 1.00 | 0.97,  1.03 |
| Age (t = 3) | 1.00 | 0.97,  1.03 | 1.00 | 0.97,  1.03 | 1.00 | 0.97,  1.03 | 1.00 | 0.97, 1.03 | 1.00 | 0.97,  1.03 |
| Income (tercile) |  |  |  |  |  |  |  |  |  |  |
| *1 (lowest)* | 1.15 | 0.85,  1.55 | 1.14 | 0.84,  1.54 | 1.13 | 0.84,  1.53 | 1.14 | 0.84,  1.54 | 1.15 | 0.85,  1.55 |
| *2* | 1.09 | 0.84,  1.41 | 1.05 | 0.81,  1.37 | 1.05 | 0.81,  1.37 | 1.05 | 0.81,  1.37 | 1.05 | 0.81,  1.37 |
| *3 (highest)* | reference |  | reference |  | reference |  | reference |  | reference |  |
| Marital status |  |  |  |  |  |  |  |  |  |  |
| *Married* | reference |  | reference |  | reference |  | reference |  | reference |  |
| *Divorced* | 0.83 | 0.57,  1.21 | 0.84 | 0.57,  1.23 | 0.84 | 0.58,  1.23 | 0.84 | 0.57,  1.23 | 0.84 | 0.58,  1.23 |
| *Single* | 1.12 | 0.76,  1.65 | 1.14 | 0.77,  1.68 | 1.13 | 0.77,  1.67 | 1.14 | 0.77,  1.68 | 1.13 | 0.77,  1.67 |
| *Widowed* | 0.97 | 0.75,  1.25 | 0.96 | 0.74,  1.25 | 0.96 | 0.74,  1.25 | 0.96 | 0.74,  1.25 | 0.97 | 0.74,  1.25 |
| Smoking |  |  |  |  |  |  |  |  |  |  |
| *Never smoker* | reference |  | reference |  | reference |  | reference |  | reference |  |
| *Former smoker* | 1.18 | 0.92,  1.52 | 1.18 | 0.92,  1.52 | 1.18 | 0.92,  1.52 | 1.18 | 0.92,  1.52 | 1.19 | 0.92,  1.52 |
| *Current smoker* | 1.60 | 1.17,  2.19 | 1.58 | 1.15,  2.16 | 1.57 | 1.15,  2.15 | 1.57 | 1.15,  2.15 | 1.56 | 1.14,  2.13 |
| BMI (kg/m^2^) | 1.02 | 1.00,  1.05 | 1.03 | 1.01,  1.05 | 1.03 | 1.01,  1.05 | 1.03 | 1.01,  1.05 | 1.03 | 1.01,  1.05 |
| Biomarker |  |  |  |  |  |  |  |  |  |  |
| *GGT* | 1.04 | 1.02,  1.06 |  |  |  |  |  |  |  |  |
| *MCV* |  |  | 0.99 | 0.94,  1.04 |  |  |  |  |  |  |
| *ALT* |  |  |  |  | 1.02 | 0.96,  1.10 |  |  |  |  |
| *AST* |  |  |  |  |  |  | 1.03 | 0.93,  1.13 |  |  |
| *CDT* |  |  |  |  |  |  |  |  | 0.93 | 0.80,  1.09 |

*Notes:* Models with income include 1925 female participants. Model 5b-5e are adjusted for age, sex, income, marital status, smoking, BMI, alcohol use and for one of the biomarkers in each case. Values of GGT, ALT and AST were divided by 10 and values of MCV and CDT were divided by two, so that the estimates now represent changes per 10-unit or 2-unit increase in the biomarker level. HR = hazard ratio; CI = confidence interval; BMI = body mass index; GGT = gamma-glutamyltransferase; ALT = alanine transaminase; AST = aspartate transaminase; MCV = mean corpuscular volume; CDT = carbohydrate-deficient transferrin.

**Table F**

*Risk of death according to income (tercile) in cox regression models 5b-5e for women.*

|  | Model 5b |  | Model 5c |  | Model 5d |  | Model 5e |  |
| --- | --- | --- | --- | --- | --- | --- | --- | --- |
|  | HR | 95% CI | HR | 95% CI | HR | 95% CI | HR | 95% CI |
| Age (t = 1) | 1.13 | 1.11,  1.16 | 1.13 | 1.11,  1.16 | 1.13 | 1.11,  1.16 | 1.13 | 1.11,  1.16 |
| Age (t = 2) | 1.00 | 0.97,  1.03 | 1.00 | 0.97,  1.03 | 1.00 | 0.97,  1.03 | 1.00 | 0.97,  1.03 |
| Age (t = 3) | 1.00 | 0.97,  1.03 | 1.00 | 0.97,  1.03 | 1.00 | 0.97,  1.03 | 1.00 | 0.97,  1.03 |
| Income (tercile) |  |  |  |  |  |  |  |  |
| *1 (lowest)* | 1.10 | 0.82,  1.49 | 1.09 | 0.81,  1.48 | 1.10 | 0.81,  1.48 | 1.11 | 0.82,  1.50 |
| *2* | 1.03 | 0.79,  1.34 | 1.03 | 0.79,  1.34 | 1.03 | 0.80,  1.34 | 1.03 | 0.79,  1.34 |
| *3 (highest)* | reference |  | reference |  | reference |  | reference |  |
| Marital status |  |  |  |  |  |  |  |  |
| *Married* | reference |  | reference |  | reference |  | reference |  |
| *Divorced* | 0.87 | 0.59,  1.27 | 0.87 | 0.59,  1.27 | 0.87 | 0.59,  1.27 | 0.87 | 0.59,  1.27 |
| *Single* | 1.15 | 0.77,  1.70 | 1.14 | 0.77,  1.69 | 1.14 | 0.77,  1.69 | 1.14 | 0.77,  1.69 |
| *Widowed* | 0.96 | 0.74,  1.24 | 0.96 | 0.74,  1.25 | 0.96 | 0.74,  1.24 | 0.96 | 0.74,  1.25 |
| Smoking |  |  |  |  |  |  |  |  |
| *Never smoker* | reference |  | reference |  | reference |  | reference |  |
| *Former smoker* | 1.20 | 0.93,  1.54 | 1.20 | 0.93,  1.54 | 1.20 | 0.93,  1.54 | 1.20 | 0.93,  1.54 |
| *Current smoker* | 1.55 | 1.13,  2.13 | 1.56 | 1.14,  2.13 | 1.56 | 1.14,  2.14 | 1.54 | 1.13,  2.11 |
| BMI (kg/m^2^) | 1.02 | 1.00,  1.04 | 1.02 | 1.00,  1.04 | 1.02 | 1.00,  1.04 | 1.02 | 1.00,  1.04 |
| Alcohol use |  |  |  |  |  |  |  |  |
| *Lifetime abstainers* | 1.26 | 0.84,  1.91 | 1.26 | 0.83,  1.90 | 1.26 | 0.84,  1.90 | 1.25 | 0.83,  1.89 |
| *Current abstainers* | 1.56 | 1.13,  2.16 | 1.58 | 1.15,  2.18 | 1.57 | 1.14,  2.17 | 1.55 | 1.13,  2.14 |
| *Low intake* | reference |  | reference |  | reference |  | reference |  |
| *Moderate intake* | 0.74 | 0.43,  1.28 | 0.74 | 0.43,  1.28 | 0.74 | 0.43,  1.28 | 0.75 | 0.43,  1.29 |
| *High intake* | 1.30 | 0.66,  2.56 | 1.30 | 0.66,  2.54 | 1.30 | 0.66,  2.55 | 1.31 | 0.67,  2.57 |
| Biomarker |  |  |  |  |  |  |  |  |
| *MCV* | 1.00 | 0.95,  1.05 |  |  |  |  |  |  |
| *ALT* |  |  | 1.03 | 0.96,  1.11 |  |  |  |  |
| *AST* |  |  |  |  | 1.03 | 0.94,  1.14 |  |  |
| *CDT* |  |  |  |  |  |  | 0.95 | 0.82,  1.12 |

*Notes:* Models with income include 1925 female participants. Model 5b-5e are adjusted for age, sex, income, marital status, smoking, BMI, alcohol use and for one of the biomarkers in each case. Values of ALT and AST were divided by 10 and values of MCV and CDT were divided by two, so that the estimates now represent changes per 10-unit or 2-unit increase in the biomarker level. HR = hazard ratio; CI = confidence interval; BMI = body mass index; MCV = mean corpuscular volume; ALT = alanine transaminase; AST = aspartate transaminase; CDT = carbohydrate-deficient transferrin.

**Table G**

*Risk of death according to income (tercile) in cox regression models 1, 2, 3, and 5a for men.*

|  | Model 1 |  | Model 2 |  | Model 3 |  | Model 5a |  |
| --- | --- | --- | --- | --- | --- | --- | --- | --- |
|  | HR | 95% CI | HR | 95% CI | HR | 95% CI | HR | 95% CI |
| Age (t = 1) | 1.09 | 1.08,  1.11 | 1.11 | 1.09,  1.12 | 1.11 | 1.09,  1.12 | 1.11 | 1.09,  1.12 |
| Age (t = 2) | 1.02 | 1.00,  1.04 | 1.02 | 1.00,  1.04 | 1.02 | 1.00,  1.04 | 1.02 | 1.00,  1.04 |
| Age (t = 3) | 1.02 | 1.00,  1.05 | 1.03 | 1.01,  1.05 | 1.03 | 1.01,  1.05 | 1.03 | 1.00,  1.05 |
| Income (tercile) |  |  |  |  |  |  |  |  |
| *1 (lowest)* | 1.87 | 1.53,  2.28 | 1.55 | 1.26,  1.91 | 1.49 | 1.21,  1.85 | 1.50 | 1.21,  1.85 |
| *2* | 1.07 | 0.89,  1.30 | 1.00 | 0.82,  1.21 | 0.98 | 0.81,  1.19 | 1.00 | 0.82,  1.21 |
| *3 (highest)* | reference |  | reference |  | reference |  | reference |  |
| Marital status |  |  |  |  |  |  |  |  |
| *Married* | reference |  | reference |  | reference |  | reference |  |
| *Divorced* |  |  | 1.83 | 1.29,  2.60 | 1.79 | 1.26,  2.54 | 1.80 | 1.27,  2.56 |
| *Single* |  |  | 2.31 | 1.59,  3.36 | 2.19 | 1.51,  3.19 | 2.10 | 1.45,  3.06 |
| *Widowed* |  |  | 1.25 | 0.95,  1.66 | 1.22 | 0.92,  1.61 | 1.17 | 0.89,  1.55 |
| Smoking |  |  |  |  |  |  |  |  |
| *Never smoker* | reference |  | reference |  | reference |  | reference |  |
| *Former smoker* |  |  | 1.16 | 0.93,  1.45 | 1.16 | 0.93,  1.46 | 1.17 | 0.94,  1.47 |
| *Current smoker* |  |  | 2.30 | 1.77,  2.99 | 2.29 | 1.75,  2.98 | 2.24 | 1.72,  2.93 |
| BMI (kg/m^2^) |  |  | 1.04 | 1.01,  1.06 | 1.04 | 1.01,  1.06 | 1.03 | 1.01,  1.05 |
| Alcohol use |  |  |  |  |  |  |  |  |
| *Lifetime abstainers* |  |  |  |  | 0.95 | 0.47,  1.93 | 0.97 | 0.48,  1.96 |
| *Current abstainers* |  |  |  |  | 1.54 | 1.17,  2.02 | 1.55 | 1.18,  2.04 |
| *Low intake* | reference |  | reference |  | reference |  | reference |  |
| *Moderate intake* |  |  |  |  | 0.89 | 0.73,  1.09 | 0.88 | 0.72,  1.08 |
| *High intake* |  |  |  |  | 1.26 | 0.97,  1.63 | 1.18 | 0.90,  1.53 |
| Biomarker |  |  |  |  |  |  |  |  |
| *GGT* |  |  |  |  |  |  | 1.01 | 1.01,  1.02 |

*Notes:* Models with income include 1846 male participants. Model 1 is adjusted for age, sex, and income; model 2 is model 1 + adjustment for marital status, smoking and BMI; model 3 is model 2 + adjustment for alcohol use; model 5a is model 3 + adjustment for GGT. Values of GGT were divided by 10, so that the estimates now represent changes per 10-unit increase. HR = hazard ratio; CI = confidence interval; BMI = body mass index; GGT = gamma-glutamyltransferase.

**Table H**

*Risk of death according to income (tercile) in cox regression models 4a-4e for men.*

|  | Model 4a |  | Model 4b |  | Model 4c |  | Model 4d |  | Model 4e |  |
| --- | --- | --- | --- | --- | --- | --- | --- | --- | --- | --- |
|  | HR | 95% CI | HR | 95% CI | HR | 95% CI | HR | 95% CI | HR | 95% CI |
| Age (t = 1) | 1.11 | 1.10,  1.13 | 1.11 | 1.09,  1.12 | 1.11 | 1.09,  1.13 | 1.11 | 1.10,  1.13 | 1.11 | 1.09,  1.12 |
| Age (t = 2) | 1.02 | 1.00,  1.04 | 1.02 | 1.00,  1.04 | 1.02 | 1.00,  1.04 | 1.02 | 1.00,  1.04 | 1.02 | 1.00,  1.04 |
| Age (t = 3) | 1.02 | 1.00,  1.05 | 1.03 | 1.01,  1.05 | 1.03 | 1.01,  1.05 | 1.02 | 1.00,  1.05 | 1.03 | 1.00,  1.05 |
| Income (tercile) |  |  |  |  |  |  |  |  |  |  |
| *1 (lowest)* | 1.56 | 1.26,  1.92 | 1.53 | 1.24,  1.89 | 1.55 | 1.26,  1.91 | 1.56 | 1.27,  1.92 | 1.54 | 1.25,  1.90 |
| *2* | 1.02 | 0.84,  1.23 | 0.97 | 0.80,  1.18 | 0.99 | 0.82,  1.20 | 1.00 | 0.82,  1.21 | 0.99 | 0.82,  1.20 |
| *3 (highest)* | reference |  | reference |  | reference |  | reference |  |  |  |
| Marital status |  |  |  |  |  |  |  |  |  |  |
| *Married* | reference |  | reference |  | reference |  | reference |  | reference |  |
| *Divorced* | 1.85 | 1.31,  2.62 | 1.94 | 1.37,  2.76 | 1.83 | 1.29,  2.60 | 1.85 | 1.31,  2.62 | 1.83 | 1.29,  2.59 |
| *Single* | 2.23 | 1.53,  3.24 | 2.34 | 1.61,  3.39 | 2.32 | 1.60,  3.37 | 2.34 | 1.61,  3.40 | 2.31 | 1.59,  3.36 |
| *Widowed* | 1.21 | 0.91,  1.59 | 1.19 | 0.90,  1.58 | 1.24 | 0.94,  1.64 | 1.20 | 0.91,  1.59 | 1.27 | 0.96,  1.67 |
| Smoking |  |  |  |  |  |  |  |  |  |  |
| *Never smoker* | reference |  | reference |  | reference |  | reference |  | reference |  |
| *Former smoker* | 1.16 | 0.93,  1.45 | 1.17 | 0.94,  1.46 | 1.16 | 0.93,  1.44 | 1.15 | 0.92,  1.43 | 1.16 | 0.93,  1.45 |
| *Current smoker* | 2.24 | 1.72,  2.91 | 2.14 | 1.64,  2.80 | 2.31 | 1.77,  3.00 | 2.29 | 1.76,  2.98 | 2.28 | 1.75,  2.97 |
| BMI (kg/m^2^) | 1.03 | 1.01,  1.05 | 1.04 | 1.02,  1.06 | 1.03 | 1.01,  1.06 | 1.03 | 1.01,  1.05 | 1.04 | 1.02,  1.06 |
| Biomarker |  |  |  |  |  |  |  |  |  |  |
| *GGT* | 1.01 | 1.01,  1.02 |  |  |  |  |  |  |  |  |
| *MCV* |  |  | 1.09 | 1.04,  1.13 |  |  |  |  |  |  |
| *ALT* |  |  |  |  | 1.03 | 0.98,  1.08 |  |  |  |  |
| *AST* |  |  |  |  |  |  | 1.11 | 1.07,  1.15 |  |  |
| *CDT* |  |  |  |  |  |  |  |  | 1.11 | 1.01,  1.22 |

*Notes:* Models with income include 1846 male participants. Model 4a-4e are adjusted for age, sex, income, marital status, smoking, BMI and for one of the biomarkers in each case. Values of GGT, ALT and AST were divided by 10 and values of MCV and CDT were divided by two, so that the estimates now represent changes per 10-unit or 2-unit increase in the biomarker level. HR = hazard ratio; CI = confidence interval; BMI = body mass index; GGT = gamma-glutamyltransferase; ALT = alanine transaminase; AST = aspartate transaminase; MCV = mean corpuscular volume; CDT = carbohydrate-deficient transferrin.

**Table I**

*Risk of death according to income (tercile) in cox regression models 5b-5e for men.*

|  | Model 5b | | Model 5c | | Model 5d | | Model 5e | |
| --- | --- | --- | --- | --- | --- | --- | --- | --- |
|  | HR | 95% CI | HR | 95% CI | HR | 95% CI | HR | 95% CI |
| Age (t = 1) | 1.11 | 1.09,  1.12 | 1.11 | 1.09,  1.12 | 1.11 | 1.10,  1.13 | 1.11 | 1.09,  1.12 |
| Age (t = 2) | 1.02 | 1.00,  1.04 | 1.02 | 1.00,  1.04 | 1.02 | 1.00,  1.04 | 1.02 | 1.00,  1.04 |
| Age (t = 3) | 1.03 | 1.01,  1.05 | 1.03 | 1.01,  1.05 | 1.03 | 1.00,  1.05 | 1.03 | 1.01,  1.05 |
| Income (tercile) |  |  |  |  |  |  |  |  |
| *1 (lowest)* | 1.48 | 1.20,  1.83 | 1.49 | 1.21,  1.84 | 1.50 | 1.21,  1.85 | 1.48 | 1.19,  1.83 |
| *2* | 0.96 | 0.79,  1.16 | 0.98 | 0.81,  1.19 | 0.98 | 0.81,  1.18 | 0.98 | 0.81,  1.18 |
| *3 (highest)* | reference |  | reference |  | reference |  | reference |  |
| Marital status |  |  |  |  |  |  |  |  |
| *Married* | reference |  | reference |  | reference |  | reference |  |
| *Divorced* | 1.89 | 1.33,  2.68 | 1.79 | 1.26,  2.54 | 1.81 | 1.27,  2.57 | 1.78 | 1.26,  2.53 |
| *Single* | 2.20 | 1.52,  3.19 | 2.20 | 1.52,  3.19 | 2.22 | 1.53,  3.22 | 2.18 | 1.50,  3.16 |
| *Widowed* | 1.16 | 0.88,  1.54 | 1.21 | 0.91,  1.60 | 1.17 | 0.88,  1.55 | 1.23 | 0.93,  1.63 |
| Smoking |  |  |  |  |  |  |  |  |
| *Never smoker* | reference |  | reference |  | reference |  | reference |  |
| *Former smoker* | 1.18 | 0.94,  1.48 | 1.16 | 0.93,  1.46 | 1.16 | 0.93,  1.45 | 1.17 | 0.94,  1.46 |
| *Current smoker* | 2.16 | 1.66,  2.83 | 2.29 | 1.76,  3.00 | 2.30 | 1.76,  3.01 | 2.28 | 1.74,  2.97 |
| BMI (kg/m^2^) | 1.04 | 1.02,  1.06 | 1.03 | 1.01,  1.06 | 1.03 | 1.01,  1.05 | 1.04 | 1.02,  1.06 |
| Alcohol use |  |  |  |  |  |  |  |  |
| *Lifetime abstainers* | 0.99 | 0.49,  2.00 | 0.95 | 0.47,  1.92 | 0.95 | 0.47,  1.94 | 0.97 | 0.48,  1.98 |
| *Current abstainers* | 1.43 | 1.09,  1.89 | 1.52 | 1.16,  2.00 | 1.52 | 1.16,  2.00 | 1.59 | 1.21,  2.09 |
| *Low intake* | reference |  | reference |  | reference |  | reference |  |
| *Moderate intake* | 0.85 | 0.69,  1.04 | 0.89 | 0.73,  1.09 | 0.87 | 0.71,  1.07 | 0.88 | 0.72,  1.08 |
| *High intake* | 1.14 | 0.87,  1.49 | 1.24 | 0.95,  1.62 | 1.15 | 0.88,  1.49 | 1.19 | 0.91,  1.56 |
| Biomarker |  |  |  |  |  |  |  |  |
| *MCV* | 1.08 | 1.04,  1.12 |  |  |  |  |  |  |
| *ALT* |  |  | 1.02 | 0.97,  1.07 |  |  |  |  |
| *AST* |  |  |  |  | 1.11 | 1.06,  1.15 |  |  |
| *CDT* |  |  |  |  |  |  | 1.12 | 1.02,  1.23 |

*Notes:* Models with income include 1846 male participants. Model 5b-5e are adjusted for age, sex, income, marital status, smoking, BMI, alcohol use and for one of the biomarkers in each case. Values of ALT and AST were divided by 10 and values of MCV and CDT were divided by two, so that the estimates now represent changes per 10-unit or 2-unit increase in the biomarker level. HR = hazard ratio; CI = confidence interval; BMI = body mass index; MCV = mean corpuscular volume; ALT = alanine transaminase; AST = aspartate transaminase; CDT = carbohydrate-deficient transferrin.

**Table J**

*Risk of death according to education in cox regression models 1, 2, 3, and 5a for women.*

|  | Model 1 |  | Model 2 |  | Model 3 |  | Model 5a |  |
| --- | --- | --- | --- | --- | --- | --- | --- | --- |
|  | HR | 95% CI | HR | 95% CI | HR | 95% CI | HR | 95% CI |
| Age (t = 1) | 1.13 | 1.10,  1.15 | 1.13 | 1.10,  1.16 | 1.13 | 1.10,  1.15 | 1.13 | 1.10,  1.16 |
| Age (t = 2) | 1.00 | 0.97,  1.03 | 1.00 | 0.97,  1.03 | 1.00 | 0.97,  1.03 | 1.00 | 0.97,  1.03 |
| Age (t = 3) | 1.00 | 0.97,  1.03 | 1.00 | 0.97,  1.03 | 1.00 | 0.97,  1.03 | 1.00 | 0.97,  1.03 |
| Education |  |  |  |  |  |  |  |  |
| < 10 years | 1.74 | 1.13,  2.68 | 1.65 | 1.06,  2.55 |  |  |  |  |
| 10 years | 1.39 | 0.87,  2.23 | 1.34 | 0.83,  2.15 |  |  |  |  |
| > 10 years | reference |  | reference |  | reference |  | reference |  |
| Marital status |  |  |  |  |  |  |  |  |
| *Married* | reference |  | reference |  | reference |  | reference |  |
| *Divorced* |  |  | 0.91 | 0.64,  1.31 | 0.93 | 0.65,  1.33 | 0.91 | 0.64,  1.31 |
| *Single* |  |  | 1.31 | 0.91,  1.88 | 1.29 | 0.90,  1.86 | 1.27 | 0.88,  1.83 |
| *Widowed* |  |  | 1.05 | 0.82,  1.34 | 1.05 | 0.82,  1.34 | 1.05 | 0.82,  1.34 |
| Smoking |  |  |  |  |  |  |  |  |
| *Never smoker* | reference |  | reference |  | reference |  | reference |  |
| *Former smoker* |  |  | 1.15 | 0.90,  1.47 | 1.17 | 0.92,  1.50 | 1.17 | 0.92,  1.50 |
| *Current smoker* |  |  | 1.66 | 1.23,  2.22 | 1.65 | 1.23,  2.22 | 1.69 | 1.26,  2.28 |
| BMI (kg/m^2^) |  |  | 1.03 | 1.01,  1.05 | 1.02 | 1.00,  1.04 | 1.02 | 1.00,  1.04 |
| Alcohol use |  |  |  |  |  |  |  |  |
| *Lifetime abstainers* |  |  |  |  | 1.15 | 0.76,  1.72 | 1.13 | 0.75,  1.70 |
| *Current abstainers* |  |  |  |  | 1.48 | 1.08,  2.01 | 1.48 | 1.09,  2.02 |
| *Low intake* | reference |  | reference |  | reference |  | reference |  |
| *Moderate intake* |  |  |  |  | 0.72 | 0.42,  1.25 | 0.67 | 0.38,  1.16 |
| *High intake* |  |  |  |  | 1.45 | 0.77,  2.76 | 1.47 | 0.77,  2.79 |
| Biomarker |  |  |  |  |  |  |  |  |
| *GGT* |  |  |  |  |  |  | 1.04 | 1.02,  1.06 |

*Notes:* Models with education include 2010 female participants. Model 1 is adjusted for age, sex, and education; model 2 is model 1 + adjustment for marital status, smoking and BMI; model 3 is model 2 + adjustment for alcohol use; model 5a is model 3 + adjustment for GGT. Values of GGT were divided by 10, so that the estimates now represent changes per 10-unit increase. HR = hazard ratio; CI = confidence interval; BMI = body mass index; GGT = gamma-glutamyltransferase.

**Table K**

*Risk of death according to education in cox regression models 4a-4e for women.*

|  | Model 4a |  | Model 4b |  | Model 4c |  | Model 4d |  | Model 4e |  |
| --- | --- | --- | --- | --- | --- | --- | --- | --- | --- | --- |
|  | Harrel’s C: |  | Harrel’s C: |  | Harrel’s C: |  | Harrel’s C: |  | Harrel’s C: |  |
|  | HR | 95% CI | HR | 95% CI | HR | 95% CI | HR | 95% CI | HR | 95% CI |
| Age (t = 1) | 1.13 | 1.10,  1.16 | 1.13 | 1.10,  1.16 | 1.13 | 1.10,  1.16 | 1.13 | 1.10,  1.16 | 1.13 | 1.10,  1.16 |
| Age (t = 2) | 1.00 | 0.97,  1.03 | 1.00 | 0.97,  1.03 | 1.00 | 0.97,  1.03 | 1.00 | 0.97,  1.03 | 1.00 | 0.97,  1.03 |
| Age (t = 3) | 1.00 | 0.97,  1.03 | 1.00 | 0.97,  1.03 | 1.00 | 0.97,  1.03 | 1.00 | 0.97,  1.03 | 1.00 | 0.97,  1.03 |
| Education |  |  |  |  |  |  |  |  |  |  |
| < 10 years | 1.66 | 1.07,  2.57 | 1.64 | 1.06,  2.55 | 1.64 | 1.06,  2.55 | 1.64 | 1.06,  2.54 | 1.63 | 1.05,  2.53 |
| 10 years | 1.38 | 0.86,  2.22 | 1.34 | 0.83,  2.15 | 1.34 | 0.83,  2.15 | 1.34 | 0.83,  2.15 | 1.34 | 0.83,  2.15 |
| > 10 years | reference |  | reference |  | reference |  | reference |  | reference |  |
| Marital status |  |  |  |  |  |  |  |  |  |  |
| *Married* | reference |  | reference |  | reference |  | reference |  | reference |  |
| *Divorced* | 0.89 | 0.62,  1.28 | 0.91 | 0.63,  1.31 | 0.91 | 0.63,  1.31 | 0.91 | 0.63,  1.31 | 0.91 | 0.64,  1.31 |
| *Single* | 1.28 | 0.89,  1.84 | 1.31 | 0.91,  1.88 | 1.30 | 0.91,  1.88 | 1.30 | 0.91,  1.88 | 1.30 | 0.91,  1.87 |
| *Widowed* | 1.05 | 0.82,  1.34 | 1.05 | 0.82,  1.34 | 1.05 | 0.82,  1.34 | 1.05 | 0.82,  1.34 | 1.05 | 0.82,  1.34 |
| Smoking |  |  |  |  |  |  |  |  |  |  |
| *Never smoker* | reference |  | reference |  | reference |  | reference |  | reference |  |
| *Former smoker* | 1.15 | 0.90,  1.47 | 1.16 | 0.91,  1.48 | 1.15 | 0.90,  1.47 | 1.15 | 0.91,  1.47 | 1.16 | 0.91,  1.48 |
| *Current smoker* | 1.69 | 1.26,  2.27 | 1.68 | 1.25,  2.26 | 1.66 | 1.24,  2.23 | 1.66 | 1.24,  2.23 | 1.65 | 1.23,  2.21 |
| BMI (kg/m^2^) | 1.02 | 1.01,  1.04 | 1.03 | 1.01,  1.05 | 1.03 | 1.01,  1.05 | 1.03 | 1.01,  1.05 | 1.03 | 1.01,  1.05 |
| Biomarker |  |  |  |  |  |  |  |  |  |  |
| *GGT* | 1.04 | 1.02,  1.06 |  |  |  |  |  |  |  |  |
| *MCV* |  |  | 0.99 | 0.94,  1.03 |  |  |  |  |  |  |
| *ALT* |  |  |  |  | 1.01 | 0.94,  1.08 |  |  |  |  |
| *AST* |  |  |  |  |  |  | 1.01 | 0.91,  1.12 |  |  |
| *CDT* |  |  |  |  |  |  |  |  | 0.94 | 0.81,  1.10 |

*Notes:* Models with education include 2010 female participants. Model 5b-5e are adjusted for age, sex, education, marital status, smoking, BMI, alcohol use and for one of the biomarkers in each case. Values of GGT, ALT and AST were divided by 10 and values of MCV and CDT were divided by two, so that the estimates now represent changes per 10-unit or 2-unit increase in the biomarker level. HR = hazard ratio; CI = confidence interval; BMI = body mass index; GGT = gamma-glutamyltransferase; ALT = alanine transaminase; AST = aspartate transaminase; MCV = mean corpuscular volume; CDT = carbohydrate-deficient transferrin.

**Table L**

*Risk of death according to education in cox regression models 5b-5e for women.*

|  | Model 5b |  | Model 5c |  | Model 5d |  | Model 5e |  |
| --- | --- | --- | --- | --- | --- | --- | --- | --- |
|  | HR | 95% CI | HR | 95% CI | HR | 95% CI | HR | 95% CI |
| Age (t = 1) | 1.13 | 1.10,  1.15 | 1.13 | 1.10,  1.15 | 1.13 | 1.10,  1.15 | 1.13 | 1.10,  1.15 |
| Age (t = 2) | 1.00 | 0.97,  1.03 | 1.00 | 0.97,  1.03 | 1.00 | 0.97,  1.03 | 1.00 | 0.97,  1.03 |
| Age (t = 3) | 1.00 | 0.97,  1.03 | 1.00 | 0.97,  1.03 | 1.00 | 0.97,  1.03 | 1.00 | 0.97,  1.03 |
| Education |  |  |  |  |  |  |  |  |
| < 10 years | 1.59 | 1.02,  2.48 | 1.58 | 1.02,  2.47 | 1.58 | 1.02,  2.46 | 1.58 | 1.02,  2.46 |
| 10 years | 1.33 | 0.82,  2.14 | 1.32 | 0.82,  2.13 | 1.32 | 0.82,  2.13 | 1.33 | 0.82,  2.14 |
| > 10 years | reference |  | reference |  | reference |  | reference |  |
| Marital status |  |  |  |  |  |  |  |  |
| *Married* | reference |  | reference |  | reference |  | reference |  |
| *Divorced* | 0.93 | 0.65,  1.33 | 0.93 | 0.65,  1.33 | 0.93 | 0.64,  1.33 | 0.93 | 0.65,  1.33 |
| *Single* | 1.29 | 0.90,  1.86 | 1.29 | 0.89,  1.86 | 1.29 | 0.89,  1.86 | 1.29 | 0.89,  1.86 |
| *Widowed* | 1.05 | 0.82,  1.34 | 1.05 | 0.82,  1.34 | 1.05 | 0.82,  1.34 | 1.05 | 0.82,  1.34 |
| Smoking |  |  |  |  |  |  |  |  |
| *Never smoker* | reference |  | reference |  | reference |  | reference |  |
| *Former smoker* | 1.17 | 0.92,  1.50 | 1.17 | 0.92,  1.50 | 1.17 | 0.92,  1.50 | 1.17 | 0.92,  1.50 |
| *Current smoker* | 1.67 | 1.24,  2.24 | 1.66 | 1.23,  2.22 | 1.66 | 1.23,  2.23 | 1.65 | 1.23,  2.21 |
| BMI (kg/m^2^) | 1.02 | 1.00,  1.04 | 1.02 | 1.00,  1.04 | 1.02 | 1.00,  1.04 | 1.02 | 1.00,  1.04 |
| Alcohol use |  |  |  |  |  |  |  |  |
| *Lifetime abstainers* | 1.14 | 0.76,  1.71 | 1.15 | 0.76,  1.72 | 1.15 | 0.76,  1.72 | 1.14 | 0.76,  1.71 |
| *Current abstainers* | 1.47 | 1.08,  2.00 | 1.48 | 1.09,  2.02 | 1.48 | 1.09,  2.02 | 1.47 | 1.08,  2.00 |
| *Low intake* | reference |  | reference |  | reference |  | reference |  |
| *Moderate intake* | 0.73 | 0.42,  1.26 | 0.72 | 0.42,  1.25 | 0.72 | 0.42,  1.25 | 0.73 | 0.42,  1.26 |
| *High intake* | 1.47 | 0.77,  2.79 | 1.45 | 0.77,  2.76 | 1.45 | 0.77,  2.76 | 1.46 | 0.77,  2.78 |
| Biomarker |  |  |  |  |  |  |  |  |
| *MCV* | 0.99 | 0.94,  1.04 |  |  |  |  |  |  |
| *ALT* |  |  | 1.01 | 0.94,  1.09 |  |  |  |  |
| *AST* |  |  |  |  | 1.02 | 0.92,  1.13 |  |  |
| *CDT* |  |  |  |  |  |  | 0.96 | 0.83,  1.12 |

*Notes:* Models with education include 2010 female participants. Model 5b-5e are adjusted for age, sex, education, marital status, smoking, BMI, alcohol use and for one of the biomarkers in each case. Values of ALT and AST were divided by 10 and values of MCV and CDT were divided by two, so that the estimates now represent changes per 10-unit or 2-unit increase in the biomarker level. HR = hazard ratio; CI = confidence interval; BMI = body mass index; MCV = mean corpuscular volume; ALT = alanine transaminase; AST = aspartate transaminase; CDT = carbohydrate-deficient transferrin.

**Table M**

*Risk of death according to education in cox regression models 1, 2, 3, and 5a for men.*

|  | Model 1 |  | Model 2 |  | Model 3 |  | Model 5a |  |
| --- | --- | --- | --- | --- | --- | --- | --- | --- |
|  | HR | 95% CI | HR | 95% CI | HR | 95% CI | HR | 95% CI |
| Age (t = 1) | 1.08 | 1.07,  1.10 | 1.10 | 1.09,  1.12 | 1.10 | 1.09,  1.12 | 1.11 | 1.09,  1.12 |
| Age (t = 2) | 1.02 | 1.00,  1.03 | 1.02 | 1.00,  1.04 | 1.02 | 1.00,  1.04 | 1.02 | 1.00,  1.03 |
| Age (t = 3) | 1.02 | 1.00,  1.05 | 1.03 | 1.01,  1.05 | 1.03 | 1.01,  1.05 | 1.03 | 1.00,  1.05 |
| Education |  |  |  |  |  |  |  |  |
| < 10 years | 1.55 | 1.22,  1.97 | 1.42 | 1.12,  1.80 | 1.39 | 1.09,  1.77 | 1.38 | 1.09,  1.76 |
| 10 years | 1.19 | 0.90,  1.58 | 1.15 | 0.87,  1.53 | 1.15 | 0.87,  1.53 | 1.13 | 0.85,  1.50 |
| > 10 years | reference |  | reference |  | reference |  | reference |  |
| Marital status |  |  |  |  |  |  |  |  |
| *Married* | reference |  | reference |  | reference |  | reference |  |
| *Divorced* |  |  | 2.00 | 1.43,  2.82 | 1.96 | 1.39,  2.75 | 1.97 | 1.40,  2.77 |
| *Single* |  |  | 2.54 | 1.78,  3.63 | 2.41 | 1.68,  3.45 | 2.31 | 1.62,  3.31 |
| *Widowed* |  |  | 1.27 | 0.97,  1.66 | 1.24 | 0.95,  1.63 | 1.20 | 0.91,  1.57 |
| Smoking |  |  |  |  |  |  |  |  |
| *Never smoker* |  |  | reference |  | reference |  | reference |  |
| *Former smoker* |  |  | 1.10 | 0.89,  1.37 | 1.10 | 0.89,  1.37 | 1.11 | 0.89,  1.38 |
| *Current smoker* |  |  | 2.40 | 1.86,  3.10 | 2.38 | 1.84,  3.09 | 2.34 | 1.80,  3.03 |
| BMI (kg/m^2^) |  |  | 1.03 | 1.01,  1.05 | 1.03 | 1.01,  1.05 | 1.03 | 1.01,  1.05 |
| Alcohol use |  |  |  |  |  |  |  |  |
| *Lifetime abstainers* |  |  |  |  | 0.98 | 0.48,  1.99 | 1.00 | 0.49,  2.02 |
| *Current abstainers* |  |  |  |  | 1.42 | 1.10,  1.84 | 1.44 | 1.11,  1.87 |
| *Low intake* |  |  |  |  | reference |  | reference |  |
| *Moderate intake* |  |  |  |  | 0.86 | 0.71,  1.05 | 0.85 | 0.70,  1.04 |
| *High intake* |  |  |  |  | 1.27 | 0.98,  1.64 | 1.18 | 0.91,  1.53 |
| Biomarker |  |  |  |  |  |  |  |  |
| *GGT* |  |  |  |  |  |  | 1.01 | 1.01,  1.02 |

*Notes:* Models with education include 1929 male participants. Model 1 is adjusted for age, sex, and education; model 2 is model 1 + adjustment for marital status, smoking and BMI; model 3 is model 2 + adjustment for alcohol use; model 5a is model 3 + adjustment for GGT. Values of GGT were divided by 10, so that the estimates now represent changes per 10-unit increase. HR = hazard ratio; CI = confidence interval; BMI = body mass index; GGT = gamma-glutamyltransferase.

**Table N**

*Risk of death according to education in cox regression models 4a-4e for men.*

|  | Model 4a |  | Model 4b |  | Model 4c |  | Model 4d |  | Model 4e |  |
| --- | --- | --- | --- | --- | --- | --- | --- | --- | --- | --- |
|  | HR | 95% CI | HR | 95% CI | HR | 95% CI | HR | 95% CI | HR | 95% CI |
| Age (t = 1) | 1.11 | 1.09,  1.12 | 1.10 | 1.09,  1.12 | 1.11 | 1.09,  1.12 | 1.11 | 1.09,  1.12 | 1.11 | 1.09,  1.12 |
| Age (t = 2) | 1.02 | 1.00,  1.03 | 1.02 | 1.00,  1.04 | 1.02 | 1.00,  1.04 | 1.02 | 1.00,  1.03 | 1.02 | 1.00,  1.04 |
| Age (t = 3) | 1.02 | 1.00,  1.05 | 1.03 | 1.01,  1.05 | 1.03 | 1.01,  1.05 | 1.02 | 1.00,  1.05 | 1.03 | 1.00,  1.05 |
| Education |  |  |  |  |  |  |  |  |  |  |
| < 10 years | 1.42 | 1.12,  1.80 | 1.42 | 1.12,  1.81 | 1.42 | 1.12,  1.80 | 1.42 | 1.12,  1.80 | 1.41 | 1.11,  1.80 |
| 10 years | 1.13 | 0.86,  1.51 | 1.19 | 0.89,  1.58 | 1.15 | 0.87,  1.53 | 1.13 | 0.85,  1.51 | 1.16 | 0.88,  1.54 |
| > 10 years | reference |  | reference |  | reference |  | reference |  |  |  |
| Marital status |  |  |  |  |  |  |  |  |  |  |
| *Married* | reference |  | reference |  | reference |  | reference |  | reference |  |
| *Divorced* | 2.03 | 1.44,  2.85 | 2.09 | 1.49,  2.94 | 2.01 | 1.43,  2.82 | 2.03 | 1.45,  2.86 | 2.00 | 1.42,  2.81 |
| *Single* | 2.46 | 1.72,  3.51 | 2.56 | 1.79,  3.65 | 2.55 | 1.78,  3.64 | 2.57 | 1.80,  3.67 | 2.53 | 1.77,  3.62 |
| *Widowed* | 1.21 | 0.93,  1.59 | 1.21 | 0.92,  1.58 | 1.26 | 0.96,  1.65 | 1.21 | 0.93,  1.59 | 1.27 | 0.97,  1.66 |
| Smoking |  |  |  |  |  |  |  |  |  |  |
| *Never smoker* | reference |  | reference |  | reference |  | reference |  | reference |  |
| *Former smoker* | 1.10 | 0.89,  1.37 | 1.12 | 0.90,  1.39 | 1.10 | 0.89,  1.37 | 1.09 | 0.88,  1.36 | 1.11 | 0.89,  1.38 |
| *Current smoker* | 2.33 | 1.81,  3.02 | 2.25 | 1.74,  2.91 | 2.41 | 1.86,  3.11 | 2.39 | 1.85,  3.09 | 2.38 | 1.84,  3.08 |
| BMI (kg/m^2^) | 1.03 | 1.01,  1.05 | 1.03 | 1.01,  1.05 | 1.03 | 1.01,  1.05 | 1.03 | 1.01,  1.05 | 1.04 | 1.01,  1.06 |
| Biomarker |  |  |  |  |  |  |  |  |  |  |
| *GGT* | 1.01 | 1.01,  1.02 |  |  |  |  |  |  |  |  |
| *MCV* |  |  | 1.08 | 1.04,  1.12 |  |  |  |  |  |  |
| *ALT* |  |  |  |  | 1.02 | 0.97,  1.07 |  |  |  |  |
| *AST* |  |  |  |  |  |  | 1.10 | 1.06,  1.15 |  |  |
| *CDT* |  |  |  |  |  |  |  |  | 1.11 | 1.01,  1.22 |

*Notes:* Models with education include 1929 male participants. Model 4a-4e are adjusted for age, sex, education, marital status, smoking, BMI and for one of the biomarkers in each case. Values of GGT, ALT and AST were divided by 10 and values of MCV and CDT were divided by two, so that the estimates now represent changes per 10-unit or 2-unit increase in the biomarker level. HR = hazard ratio; CI = confidence interval; BMI = body mass index; GGT = gamma-glutamyltransferase; ALT = alanine transaminase; AST = aspartate transaminase; MCV = mean corpuscular volume; CDT = carbohydrate-deficient transferrin.

**Table O**

*Risk of death according to education in cox regression models 5b-5e for men.*

|  | Model 5b | | Model 5c | | Model 5d | | Model 5e | |
| --- | --- | --- | --- | --- | --- | --- | --- | --- |
|  | HR | 95% CI | HR | 95% CI | HR | 95% CI | HR | 95% CI |
| Age (t = 1) | 1.10 | 1.09,  1.12 | 1.10 | 1.09,  1.12 | 1.11 | 1.09,  1.12 | 1.10 | 1.09,  1.12 |
| Age (t = 2) | 1.02 | 1.00,  1.04 | 1.02 | 1.00,  1.04 | 1.02 | 1.00,  1.03 | 1.02 | 1.00,  1.04 |
| Age (t = 3) | 1.03 | 1.01,  1.05 | 1.03 | 1.01,  1.05 | 1.03 | 1.01,  1.05 | 1.03 | 1.01,  1.05 |
| Education |  |  |  |  |  |  |  |  |
| < 10 years | 1.39 | 1.09,  1.77 | 1.39 | 1.09,  1.77 | 1.38 | 1.09,  1.76 | 1.38 | 1.08,  1.75 |
| 10 years | 1.18 | 0.89,  1.57 | 1.15 | 0.87,  1.52 | 1.13 | 0.85,  1.49 | 1.16 | 0.87,  1.54 |
| > 10 years | reference |  | reference |  | reference |  | reference |  |
| Marital status |  |  |  |  |  |  |  |  |
| *Married* | reference |  | reference |  | reference |  | reference |  |
| *Divorced* | 2.03 | 1.44,  2.86 | 1.96 | 1.39,  2.75 | 1.98 | 1.41,  2.78 | 1.95 | 1.38,  2.74 |
| *Single* | 2.41 | 1.69,  3.45 | 2.41 | 1.69,  3.45 | 2.43 | 1.70,  3.47 | 2.38 | 1.66,  3.40 |
| *Widowed* | 1.19 | 0.91,  1.56 | 1.24 | 0.94,  1.62 | 1.19 | 0.91,  1.57 | 1.25 | 0.95,  1.63 |
| Smoking |  |  |  |  |  |  |  |  |
| *Never smoker* | reference |  | reference |  | reference |  | reference |  |
| *Former smoker* | 1.12 | 0.90,  1.39 | 1.10 | 0.88,  1.37 | 1.10 | 0.88,  1.37 | 1.12 | 0.90,  1.39 |
| *Current smoker* | 2.26 | 1.74,  2.94 | 2.39 | 1.84,  3.10 | 2.40 | 1.85,  3.11 | 2.37 | 1.83,  3.08 |
| BMI (kg/m^2^) | 1.03 | 1.01,  1.05 | 1.03 | 1.01,  1.05 | 1.03 | 1.01,  1.05 | 1.04 | 1.01,  1.06 |
| Alcohol use |  |  |  |  |  |  |  |  |
| *Lifetime abstainers* | 1.01 | 0.50,  2.06 | 0.98 | 0.48,  1.98 | 0.98 | 0.48,  1.98 | 1.00 | 0.49,  2.04 |
| *Current abstainers* | 1.35 | 1.04,  1.76 | 1.41 | 1.09,  1.84 | 1.41 | 1.09,  1.83 | 1.47 | 1.13,  1.91 |
| *Low intake* | reference |  | reference |  | reference |  | reference |  |
| *Moderate intake* | 0.82 | 0.68,  1.00 | 0.86 | 0.71,  1.04 | 0.84 | 0.69,  1.02 | 0.85 | 0.70,  1.03 |
| *High intake* | 1.16 | 0.89,  1.51 | 1.26 | 0.97,  1.63 | 1.16 | 0.89,  1.50 | 1.20 | 0.92,  1.56 |
| Biomarker |  |  |  |  |  |  |  |  |
| *MCV* | 1.07 | 1.03,  1.12 |  |  |  |  |  |  |
| *ALT* |  |  | 1.01 | 0.96,  1.06 |  |  |  |  |
| *AST* |  |  |  |  | 1.10 | 1.06,  1.15 |  |  |
| *CDT* |  |  |  |  |  |  | 1.12 | 1.02,  1.23 |

*Notes:* Models with education include 1929 male participants. Model 5b-5e are adjusted for age, sex, education, marital status, smoking, BMI, alcohol use and for one of the biomarkers in each case. Values of ALT and AST were divided by 10 and values of MCV and CDT were divided by two, so that the estimates now represent changes per 10-unit or 2-unit increase in the biomarker level. HR = hazard ratio; CI = confidence interval; BMI = body mass index; MCV = mean corpuscular volume; ALT = alanine transaminase; AST = aspartate transaminase; CDT = carbohydrate-deficient transferrin.
